# Feasibility of a Co-Designed Personalized Cognitive Reablement Program Delivered via Digital Voice Assistants for Older Adults Living With Early-Stage Dementia or Mild Cognitive Impairment: Pilot Randomized Controlled Trial

**DOI:** 10.2196/96922

**Published:** 2026-10-23

**Authors:** Tracy Yiannis, Helen Macpherson, David Scott, Michele Callisaya, Celeste Chapple, Costas Glavas, Caitlyn Gourlay, Ralph Maddison, Eugene Gvozdenko, Paul Jansons

**Affiliations:** 1 Deakin Institute for Physical Activity and Nutrition, Deakin University, 221 Deakin Hwy, Burwood, Victoria, 3125, Australia, 61 415485495; 2 School of Clinical Sciences at Monash Health, Monash University, Melbourne, Victoria, Australia; 3 Menzies Institute for Medical Research, University of Tasmania, Hobart, Tasmania, Australia; 4 Peninsula Clinical School, School of Translational Medicine, Monash University, Melbourne, Victoria, Australia; 5 Great Australian Pty Ltd, Keysborough, Australia

**Keywords:** digital voice assistants, reablement, assistive technology, cognitive rehabilitation, dementia, mild cognitive impairment, digital health, feasibility, Amazon Alexa, eHealth, older adults

## Abstract

**Background:**

Home-based reablement programs have improved everyday functioning for people living with early-stage dementia or mild cognitive impairment (MCI), but the substantial time commitment required for delivery by health professionals has created a barrier to widespread implementation. Digital technology could facilitate delivery, but traditional devices such as smartphones and tablets present usability challenges for this population. We have co-designed a cognitive reablement program, VOICE-COG, that can deliver personalized interventions broadcast via voice-controlled digital voice assistants (DVAs).

**Objective:**

This pilot nonblinded randomized controlled trial examined the feasibility and potential effectiveness of VOICE-COG for older adults living with dementia or MCI to guide the design of a full-scale trial.

**Methods:**

Older adults (aged 60‐85 years) with a self-reported diagnosis of dementia or MCI (N=30) were recruited via Facebook or the StepUp-for Dementia Research database and randomized to either a DVA intervention (n=15) or control (n=15) group for 12 weeks. Each participant randomized to the intervention group and their care partner met with a psychologist via Zoom to select 2 or 3 personalized goals from the VOICE-COG program. The personalized program was input into the software (BuddyLink) by researchers for broadcast via a DVA (Amazon Alexa Show 8). Participants in the control group received enhanced usual care, consisting of treatment as usual, plus weekly informational emails. Primary feasibility outcomes were recruitment, retention, device-recorded adherence, and the System Usability Scale (SUS). Secondary outcomes were self-reported by the participant and care partner via web-based Qualtrics forms and included the Goal Attainment Scale–Light (GAS), changes in participant mood, cognition, and everyday functioning, and carer burden.

**Results:**

The recruitment rate was 86% (30/35) and the retention rate was 97% (29/30), with 1 participant in the intervention group lost to follow-up. Adherence to the VOICE-COG program ranged from 29% to 100% (mean 70%, SD 17%). The VOICE-COG program was rated as marginally acceptable (mean SUS score 56.3, SD 18.1). After 12 weeks, 60% (9/15) of the intervention group reported having met or exceeded their goals (GAS-Light t-score ≥50; mean 57.6, SD 15.4). The intervention group had a significantly higher increase in Multifactorial Memory Questionnaire–Strategy subscale score compared with control, mean difference 5.0 (95% CI 0.03-10.1, *P*=.049), representing a medium effect size (*d*=0.55). There were no other significant differences between groups.

**Conclusions:**

This co-designed VOICE-COG program, delivered via DVAs, was feasible for older adults living with early-stage dementia or MCI, resulting in most participants meeting their reablement goals and increases in the use of memory strategies. System usability issues should be addressed in future adequately powered trials.

## Introduction

Dementia is a growing public health concern worldwide due to its increasing prevalence and the associated burden of disability and dependency [1]. By 2030, the number of people living with dementia is predicted to reach 85 million, driven by the aging population and higher incidence rates in older adults [2]. As loss of cognitive functioning progresses, people living with dementia become increasingly dependent on informal and formal care, which was estimated to cost US $1.3 trillion globally in 2019 [3,4]. With limited pharmacological options to manage symptoms, studies have focused on nonpharmacological interventions to maintain independence at home and reduce the associated cost of care [5,6].

Previous research in reablement, also referred to as cognitive rehabilitation, has found that people living with dementia experience significant improvements in goal attainment related to daily functioning [7-9], while care partners reported enhanced well-being and coping outcomes [10,11]. Evidence from these studies suggests people living with mild or early-stage dementia experience greater benefits, with dementia severity a barrier to the collaborative goal-setting process [8,12]. However, the substantial time commitment for weekly home-based delivery from a qualified health professional (typically a nurse, occupational therapist, or psychologist), together with travel time and specialized training, has presented significant barriers to widespread implementation [13,14]. Technology could facilitate the remote delivery of reablement programs to overcome these barriers, increasing the availability of goal-oriented reablement programs for people living with dementia [15].

Recent studies have found telehealth delivery of reablement interventions just as effective as face-to-face home-based programs, eliminating the travel time requirement for health professionals [16,17]. This approach, however, still required a substantial time commitment from health professionals, as observed in the adapted Care of People with dementia in their Environments (COPE) [11] trial, which required 2 days training plus eight 1-hour sessions per person [17]. To date, no studies have determined if technology could improve support further by offering reablement self-management and remote monitoring from health professionals. A major issue in digital health research for this population concerns device usability, which is limited by the cognitive burden of remembering passwords and program navigation, in addition to age-related dexterity and vision impairments [18].

Digital voice assistants (DVAs) with embedded speakers and display screens that can broadcast personalized programs and interpret voice commands for navigation may offer a possible solution [19,20]. Existing evidence on DVA use for people living with dementia is confined to exploratory qualitative designs or technical usability evaluations [21-24]. These studies have suffered from methodological limitations such as case study designs, small sample sizes, and laboratory settings, limiting generalization to community-dwelling older adults living with dementia. Furthermore, while these studies provide evidence of daily activity reminders, cognitive stimulation, and social interaction, no studies have evaluated the use of DVAs to deliver a holistic, goal-based reablement program.

To address this need, we developed VOICE-COG, a cognitive reablement program delivered via DVA following a remote goal-setting meeting with a health professional. VOICE-COG is delivered to each participant’s DVA in their homes using BuddyLink (Great Australian Pty Ltd) proprietary software [25] that broadcasts personalized health programs via audio, images, text, and video. Previous pilot studies using BuddyLink have demonstrated the feasibility of DVA to deliver personalized exercise and nutrition programs for cognitively healthy older adults [26-28]. The development and testing of VOICE-COG was expanded to include older adults living with mild cognitive impairment (MCI), given its characterization as a predementia stage with similar cognitive symptoms. Co-designed with older adults living with dementia/MCI, their care partners, and health professionals, VOICE-COG is intended to support reablement for regular leisure and ad hoc appointments, emotional regulation exercises, reminiscence activities, self-care adherence, and passive cognitive stimulation [29]. The primary aim of this pilot randomized controlled trial (RCT) was to determine the feasibility and usability of VOICE-COG for older adults living with dementia to inform a full-scale trial. Secondary aims were to assess potential efficacy on goal attainment, cognition, emotional well-being, daily activity functioning, and care partner burden.

## Methods

### Study Design and Randomization

This 12-week nonblinded pilot RCT examined the feasibility of VOICE-COG, a co-designed cognitive reablement program broadcast via DVA [29], with reporting guided by the Consolidated Standards of Reporting Trials of Electronic and Mobile Health Applications and Online Telehealth (CONSORT-EHEALTH) [30] (Checklist 1). Participants were recruited using convenience sampling, with eligible participants enrolled by the primary project lead. Following completion of baseline assessments, participants were block randomized (1:1), stratified by sex, to either the intervention (VOICE-COG program) or control group. The random allocation sequence was generated in advance using a computer-based randomization method in Microsoft Excel by a research assistant, who was independent of intervention delivery. The research assistant assigned participants according to the pregenerated sequence and notified the project lead of group allocation. Participants were not blinded to group allocation due to the provision of a DVA for the intervention group. Blinding was not possible for the lead researcher, who conducted a midtrial check-in with participants as required for this feasibility pilot study.

### Participants and Research Setting

Eligibility criteria included older adults (aged 60 to 85 y) with a self-reported, recent (≤6 mo) clinical diagnosis of dementia and/or MCI, living anywhere in Australia with access to a residential Wi-Fi network. To guarantee an optimal internet connection for the Amazon Alexa DVA, the use of mobile data connections was not supported. Participants could be community-dwelling or in residential aged care, provided they had a care partner with at least 50% close contact during the 12-week trial and had access to a smartphone capable of making and receiving calls on an Australian network. Participants were required to be fluent in English. Digital literacy was not considered an inclusion criterion, as previous research has found prior experience does not influence adaptation for people living with dementia [31], and the voice navigation function of the DVA was expected to reduce this barrier. However, to reduce potential bias, all intervention participants were emailed personalized instructions on how to access their program via the DVA. Participants were excluded if they (1) were deemed to have difficulty communicating with a DVA due to speech or hearing problems, (2) were unwilling to be randomized, (3) planned to be away for ≥4 weeks during the 12-week intervention period, or (4) had any self-reported cognitive or physical impairment that, in the opinion of the research team, would result in the participant having difficulty interacting with the DVA or performing the VOICE-COG program safely. This included a self-reported clinical diagnosis of schizophrenia, bipolar disorder, or other psychotic disorders.

Participants were recruited via 2 methods. First, through paid targeted Facebook (Meta Platforms, Inc) advertisements, potential participants clicked an embedded hyperlink to read the plain language statement and completed an expression of interest form via Qualtrics. Second, eligible volunteers matching the study’s inclusion criteria from the StepUp for Dementia Research database were contacted via phone call or email [32]. Individuals in this database have previously provided consent to be contacted for research. Interested participants were screened for eligibility via telephone and then emailed a link to the plain language and consent form on Qualtrics to provide written or electronic informed consent. Participants enrolled in the study provided details of the clinician responsible for their diagnosis, who was notified of the participant’s eligibility and was given the opportunity to raise any concerns regarding participation. No further diagnostic criteria or cognitive assessment tools were used to determine eligibility.

### Intervention

As detailed elsewhere [29], the development of VOICE-COG was guided by the IDEAS (Integrate, Design, Assess, and Share) framework [33], which combines co-design practice with identification of health behavior change strategies for digital interventions. The initial health professional facilitated goal-setting meeting used a modified, dementia-specific SMART (Specific, Measurable, Achievable, Relevant, and Time-bound) framework [34,35] that establishes current functioning and targeted goal attainment, aligned with previous hybrid digital health interventions [36,37]. The subsequent VOICE-COG program, broadcast via DVA, included several evidence-based behavior change techniques [36,38], such as motivational messages and preferences for social reward [39], individual tailoring with personalized audio and text prompts, and visual cues [40], in addition to instructional videos [41].

Intervention group participants selected goals from the VOICE-COG program that were personalized and broadcast via a DVA in their homes. Participants randomized to the intervention group were mailed a preregistered Amazon Echo Show 8 device (“Alexa”) with instructions on how to connect the device to their home Wi-Fi. Each participant and their care partner were emailed a 1-page summary of the VOICE-COG program (Multimedia Appendix 1) in advance of meeting with a psychologist (qualified to a minimum Doctorate in Clinical Psychology with experience in motivational interviewing) via Zoom. Guided by the goal-setting reablement guide for people living with dementia [35], the psychologist assisted the participant in collaboratively setting 2 or 3 modified SMART-goals with their care partner that could be supported by the VOICE-COG program. Examples of participant goals can be found in Multimedia Appendix 2. These included (1) activities and appointments (attending regular weekly community activities and remembering appointments); (2) routine reminders (adherence to daily medication, hydration, and nutrition); (3) family and friends (writing their life story and maintaining relationships); (4) mood monitor (providing coping activities such as music and mindfulness); (5) active minds (providing cognitive stimulation through audiobooks, podcasts, news, and short videos); (6) household manager (remembering when and how to do home chores); and (7) kitchen helper (support for cooking favorite recipes). Further descriptions of how participants interact with the VOICE-COG modules can be found in Multimedia Appendix 3.

VOICE-COG used BuddyLink software to broadcast each participant’s personalized program via an Alexa Skill on their Amazon Show 8 DVAs, similar to an app on a smartphone. Personalized goal-related information and preferred program broadcast timings were entered into an online Qualtrics form by the psychologist during the goal-setting meeting. Each participant’s goal-related content was uploaded remotely to the BuddyLink software [32] by a research team member, who also configured program broadcast routines using deidentified individual Alexa accounts. A voice-initiated routine was added, “Alexa, Open Mind Buddy,” for participants to access their program outside these times. Following the meeting, the intervention group was emailed a personalized 2-page instruction summary for the selected VOICE-COG goals. Participants who elected to write their life story (family and friends goal) received weekly emails with a link to an online Qualtrics form for data collection. Throughout the trial, all intervention participants received fortnightly emails requesting notification of any technical errors via completion of an online Qualtrics form. An optional midtrial check-in by a research team member was offered to participants to address any concerns regarding their program.

### Control

Control group participants received usual care from their medical provider or aged care service, plus weekly emails with Dementia Australia factsheets (available from their website) [42]. These were provided for ethical reasons, to ensure participants received some benefit while minimizing the potential impact on study outcomes.

### Primary Outcome Measures

#### Feasibility

Participant recruitment, retention, and adherence rates were used to examine the feasibility of the VOICE-COG program to inform a future RCT. Recruitment rate (%) was defined as the total number of participants who provided consent at baseline divided by the total number eligible. Retention rate (%) was specified as the number of participants who completed the 12-week postintervention data collection divided by the number of participants randomized. The customized nature of the intervention allowed participants to have substantial variation in the type and number of weekly goal-related prompts. As such, adherence was defined as at least one weekly interaction with a personalized VOICE-COG goal via the DVA, as measured by a voice response to the initial dialog prompt recorded by the BuddyLink system (excluding “no” or “stop” responses). Adherence (%) was calculated as the proportion of weekly goal interactions recorded over the 12 weeks (24 or 36 possible goal interactions for participants selecting 2 or 3 goals, respectively). This measure, together with the predefined targets of ≥70% retention and ≥66% adherence to the VOICE-COG program, was based on previous evidence from DVA health intervention studies [26-28].

#### System Usability

Perceived usability of the VOICE-COG skill was assessed by DVA participants using the System Usability Scale (SUS) [43] form administered online via Qualtrics. The SUS uses 10 Likert-scale questions to determine subjective usability, providing a score from 0 to 100.

### Secondary Outcome Measures

#### Goal Attainment

All data for baseline and 12-week outcomes were collected remotely via Qualtrics (unless stated otherwise). Demographic data were also collated at baseline. Participants and care partners were emailed a link to the survey including the questionnaires and informed they could complete the survey at any time throughout the week. They were advised that they could save their progress and return to it later if necessary.

DVA participants’ goal attainment was measured using Goal Attainment Scale (GAS)–Light, a valid and reliable scoring system recommended for use with people living with dementia/MCI in reablement programs [35,44,45]. The intervention used a co-designed reablement program specifically developed for DVA delivery, thereby enabling administration by health professionals with minimal reablement training. Given the primary aim of this study was to establish feasibility, the control group did not receive a goal-setting-only intervention that would have required a reablement-trained health professional. The baseline goal attainment score was entered by the psychologist into the online Qualtrics goal-setting form during the goal-setting meeting (–2: no function or –1: some function). After 12 weeks, participants were emailed their personal goals and, together with their care partner, reported if they had achieved their goal and to what level: yes (+2 a lot more; +1 a little more; 0 as expected) or no (–0.5 partially achieved; –1 no change; –2 got worse). Results were entered into the GAS-Light spreadsheet [46] to calculate each participant’s GAS-Light t-score, where t≥50 is interpreted as met or exceeded expectations.

#### Participant Questionnaires

Secondary outcome measures included differences in the following participant questionnaires (measuring mood, memory, and cognition) completed via online Qualtrics forms at baseline and at 12 weeks. The Geriatric Depression Scale (GDS) consists of 15 items rated “yes” or “no” to screen for depression in older adults and has been validated for use in older adults with mild to moderate dementia [47]. Scores of >5 suggest depression may be present; >10 is indicative of major depression. The Multifactorial Memory Questionnaire (MMQ) is a memory evaluation tool with 57 items covering 3 subscales—memory satisfaction, ability, and strategy use [48]. Participants’ subjective memory experiences from the previous 2 weeks are rated using a 5-point Likert scale from 0 to 4. If there were <4 missing responses for any scale, scores were calculated using the prorated formula; for ≥4 missing items, participant scores were excluded from the analysis [48]. The Illness Cognition Questionnaire (ICQ) is an 18-item questionnaire using a 4-point Likert scale from 1 (disagree) to 4 (strongly agree) to measure the respondents' perception of their own illness with 3 subscales of acceptance, disease benefit, and helplessness [49]. For participants with 1 missing item in a subscale, the missing item was imputed using the mean of the other completed items within that subscale (provided at least 4 of 6 items were present).

#### Care Partner Questionnaires

At baseline and 12 weeks, care partners completed the following surveys: the Bayer Activities of Daily Living Scale (B-ADL) is a 25-item questionnaire assessing the difficulty that the person living with dementia/MCI has in performing daily activities, using a Likert scale of 0 (never) to 10 (always) [50]. The B-ADL score sums all items, excluding those rated “not applicable” or “unknown” divided by the number of items rated. The Everyday Cognition (ECog) Scale includes 39-items across 6 domains (everyday memory, language, visuospatial abilities, planning, organization, and divided attention) [51]. The Zarit Caregiver Burden 22-item questionnaire asked respondents to rate the level of burden caused using a Likert scale of 1 (“none”) to 5 (“nearly always”), giving a score range of no burden (22 to 46), mild burden (47 to 55) or severe burden (56-110) [52].

### Data Analysis

We recruited 30 participants (n=15 in each arm), consistent with sample size guidelines for pilot feasibility trials [53] and sufficient to provide preliminary estimates of medium effect sizes (*d*=0.5) to inform the design of a fully powered RCT [54].

Descriptive data for feasibility and secondary outcomes are presented as either mean and SD or frequency (%). Differences in GAS-Light t-scores for the intervention group were compared using a 2-tailed paired-sample *t* test. SUS scores were entered into the web-based analysis toolkit [55] to provide a box plot for visual comparison with the adjective quartile SUS score [56]. Differences in participant and care partner questionnaires were compared between groups using analysis of covariance adjusted for baseline values, and standardized effect sizes calculated using Cohen *d*. Missing data within scales was replaced by the mean value of item responses for that subscale. Effect estimates are reported with 95% CIs and *P* values set at *P* <.05, but as a pilot feasibility trial, this study was not powered to detect any significant changes. Shapiro-Wilk tests assessed assumptions of normality and homogeneity of variance, and natural logarithmic transformations were applied where required before conducting analysis of covariance analysis. All analyses were conducted using Stata Standard Edition (version 18.0; StataCorp).

### Ethical Considerations

This study was approved by the Deakin University Human Research Ethics Committee (HREC 2022-043) and registered with the ANZCTR (Australian New Zealand Clinical Trials Registry: ACTRN12623000898651). Intervention participants received an Alexa Show 8 device, which they could retain after the trial to compensate them for their time. Control participants received an Alexa Show 8 device after the 12-week trial was completed.

## Results

### Overview

Participant flow is presented in Figure 1. In total, 145 expressions of interest were received, with 99 potential participants successfully contacted and screened between September 2023 and June 2024. A total of 30 participants completed baseline assessments and were randomly allocated to the DVA group (n=15) or the control group (n=15).

**Figure 1.**
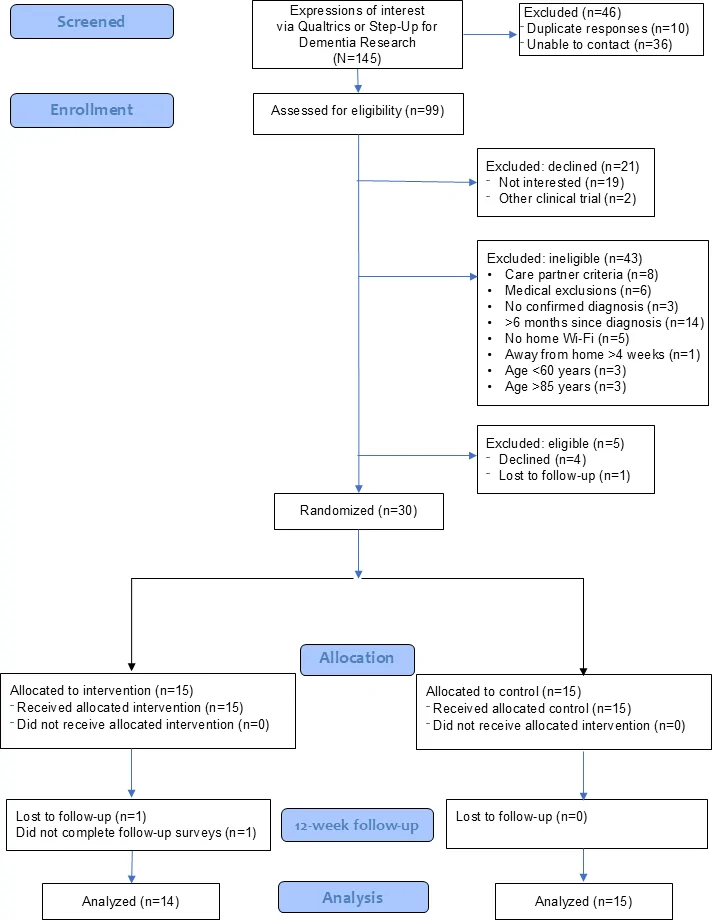
Consolidated Standards of Reporting Trials (CONSORT) diagram showing participant flow through the 12-week VOICE-COG pilot feasibility randomized controlled trial.

### Participant Demographics

Table 1 summarizes baseline demographics of the 30 participants. Participants’ ages ranged from 60 to 85 years (mean 74.7, SD 5.2 y), and the majority (n=19, 63%) were women. Over half (n=18, 60%) were educated to university level, and while most (n=24, 80%) participants were retired and 13% (n=4) receiving a pension, 7% (n=2) were still employed. The vast majority (n=28, 93%) reported having at least one chronic health condition in addition to dementia or MCI, with over half (n=17, 57%) having hypertension.

**Table 1.** Baseline participant characteristics of the DVA^a^ intervention and enhanced usual care control groups (N=30).

Demographics	DVA group (n=15)	Control group (n=15)
Age (y), mean (SD)	72.9 (5.1)	76.5 (4.9)
Sex (female), n (%)	10 (67)	9 (60)
Parent’s country of birth, n (%)		
Australia	11 (73)	10 (67)
Other	4 (27)	5 (33)
Highest level of education, n (%)		
Secondary or high school	3 (20)	4 (27)
Technical or further education institution	3 (20)	2 (13)
University or other higher education institution	9 (60)	9 (60)
Marital status, n (%)		
Married or de facto	13 (87)	14 (93)
Divorced	2 (13)	1 (7)
Current employment status, n (%)		
Employed or self-employed part-time	2 (13)	0 (0)
Pension (including disability or sole pension)	2 (13)	2 (13)
Retired	11 (73)	13 (87)
Medical conditions, n (%)		
Any form of cancer	2 (13)	2 (13)
Asthma	2 (13)	1 (7)
Cardiovascular disease^b^	5 (33)	4 (27)
Hypercholesterolemia (high cholesterol)	6 (40)	7 (47)
Hypertension (high blood pressure)	7 (47)	10 (67)
Osteoarthritis	2 (13)	5 (33)
Thrombosis (blood clot)	0 (0)	1 (7)
Rheumatoid arthritis	1 (7)	1 (7)
Diabetes	2 (13)	2 (13)
Other major illness^c^	7 (47)	6 (40)
Reported at least one chronic health condition	13 (87)	15 (100)

a DVA: digital voice assistant.

b Cardiovascular disease included angina, stroke, peripheral vascular disease, and heart attack.

c Parkinson disease, multiple sclerosis, Crohn disease, kidney disease, liver disease, transplant, congenital deafness, vertigo, Sjögren syndrome, dumping syndrome, granulomatous disease, sleep apnea, hypoparathyroidism, hypothyroidism, hypoglycemia, melanoma, dystonic tremor, Marfan syndrome, depression, ankylosing spondylitis, and grand mal epilepsy.

### Primary Outcomes

#### Feasibility

Primary outcome measures are summarized in Table 2. The recruitment rate was 86%; that is, of the 35 eligible participants, 30 people living with dementia/MCI and their care partners provided consent to participate. The overall retention rate was 97% (n=29). Retention was 100% (15/15) for the control group and 93% (14/15) for the intervention group, with 1 participant lost to follow-up (reason unknown). Adherence to the VOICE-COG program ranged from 29% to 100%, with a mean of 70% (SD 17%), exceeding the target adherence of 66%, with no adverse events. As shown in Figure 2, mean participant adherence met or exceeded the target for all VOICE-COG program goals except Activities and Appointments. Participants nominated between 7 and 52 VOICE-COG broadcasts per week, with a mean of 12 (SD 12) weekly voice interactions recorded by the DVA, as detailed in Multimedia Appendix 4.

**Table 2.** Primary outcomes of feasibility and usability.

Outcome	Time point	DVA group (n=15)	Control group (n=15)
Recruitment, n/N (%)	N/A^a^	30/35 (86)	30/35 (86)
Retention^b^, n/N (%)	12 weeks	14/15 (93)	15/15 (100)
Adherence (%), mean (SD)^c^	Overall	70 (17)	N/A
System Usability Scale (SUS), mean (SD)^d^	12 weeks	56.3 (18.1)	N/A

a N/A: not applicable.

b Retention was defined as participants who completed all postintervention assessments at 12 weeks.

c Adherence was defined as ≥1 positive response to the program goal per week (out of the number of weekly goals broadcast over 12 weeks), reported as the percentage completed.

d Calculated as a score from 1 to 100.

**Figure 2.**
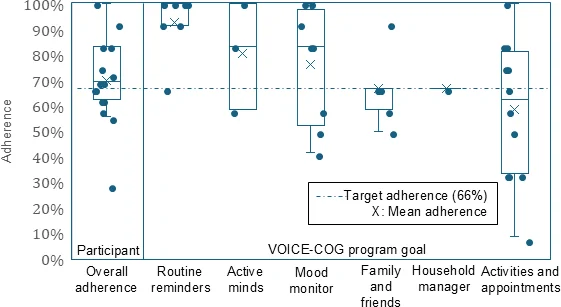
Box plots showing percentage adherence to the digital voice assistant–delivered VOICE-COG cognitive reablement program over 12 weeks, overall and by program goal.

#### System Usability

At 12 weeks, the SUS scores for DVA intervention participants indicated marginal acceptability of the VOICE-COG Program (mean 56.3, SD 18.1; range 28‐79) [56]. The distribution of scores and responses to specific SUS questions can be found in Multimedia Appendix 5. Among the 15 participants, (n=11, 73%) agreed they would like to use the system frequently, and two-thirds (n=10, 67%) agreed the system was easy to use and that most people would learn to use the system very quickly. Just over half (n=8, 53%) believed the various functions in the system were well integrated and disagreed that the system was very cumbersome to use. However, just over half (n=8, 53%) felt they needed to learn a lot of things before they could get going with this system.

### Secondary Outcomes

#### Goal Attainment

Among the 15 DVA participants, a mean of 2.6 (SD 0.5) goals was selected, with the majority (n=9, 60%) selecting 3 goals. DVA participants reported a significant increase in goal attainment, from a mean GAS-Light t-score of 36.8 (SD 0.7) at baseline to 57.6 (SD 15.4) at 12 weeks (*P*<.001). Most participants (n=9, 60%) met or exceeded their goals, as determined by a GAS-Light t-score of ≥50.

Thirty-nine VOICE-COG goals were set by participants overall, with 37 assessed at 12 weeks (1 participant who selected 2 goals was lost to follow-up). Further details, including example goals and attainment levels by VOICE-COG program goal, can be found in Multimedia Appendix 2. Of the 15 intervention group participants, 12 (80%) selected the activities and appointments assistant goal, 8 (53%) selected mood monitor, 7 (47%) selected family and friends, 6 (40%) selected routine reminders, 4 (27%) selected active minds, and just 1 (6.6%) participant selected household manager as a reablement goal. The kitchen helper program goal had to be withdrawn after 2 weeks due to technical issues with device response timing and voice command difficulties with navigating recipes. Over two-thirds of VOICE-COG program goals (25/37, 68%) were attained at the expected level or above, with over a third (13/37, 35%) achieved “a lot more” than expected. Only 1 participant reported “getting worse” on a goal related to writing their life story (family and friends). Dichotomized goal attainment (scores of 0, +1, +2=goal met; scores of –0.5, –1, or –2=goal unmet) by VOICE-COG goal is shown in Figure 3.

**Figure 3.**
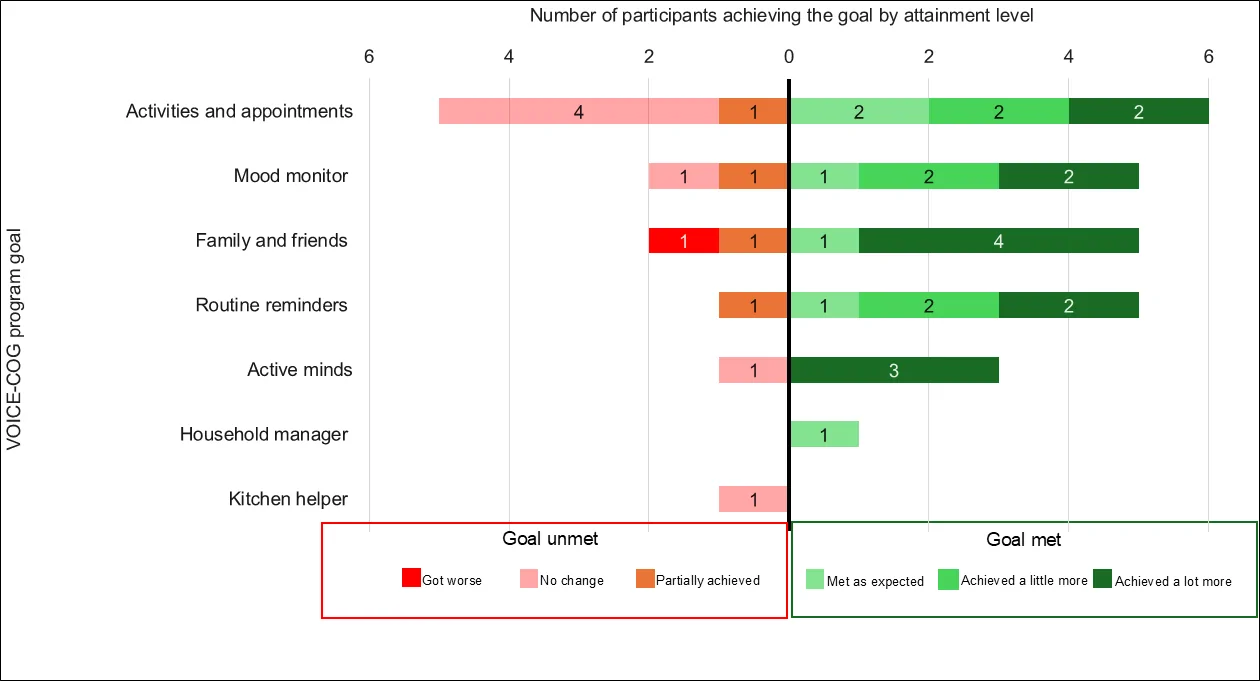
Number of digital voice assistant intervention group participants achieving each goal attainment level after 12 weeks by VOICE-COG program goal.

#### Participant and Care Partner Questionnaires

Table 3 summarizes changes in participant and care partner questionnaire scores for both the intervention and control groups. After 12 weeks, the intervention group participants reported a significant increase in the MMQ-Strategy score (mean difference 5.0, 95% CI 0.03-10.1; *P*=.049) compared to control. This represented a medium size effect (*d*=+0.5). There were no other significant differences between groups after 12 weeks.

**Table 3.** Mean scores for participant and care partner questionnaires at baseline and 12 weeks by DVA intervention and enhanced usual care control groups, with estimated between-group effect sizes (95% CI).

Outcome measure	Control^a^, mean (SD)		DVA^b^, mean (SD)		Estimated difference between groups		Effect size
	Baseline (n=15)	12 weeks (n=15)	Baseline (n=15)	12 weeks (n=14)	Effect size (95% CI)^c^	*P* value	Cohen *d*
Self-reported questionnaires							
GDS-SF^d^	5.5 (3.3)	6.7 (4.4)	3.3 (3.3)	3.5 (2.9)	–1.4 (–3.6 to 0.7)	.19	–0.39
Multifactorial Memory Questionnaire (MMQ)							
Satisfaction	28.9 (16.2)	28.1 (13.5)	29.9 (8.9)	32.2 (9.8)	3.4 (–2.4 to 9.2)	.24	0.29
Ability	34.6 (10.5)	34.1 (14.7)	42.1 (9.3)	42.2 (11.4)	1 (–6.5 to 8.5)	.79	0.08
Strategies	35.1 (8.3)	33.5 (10.1)	37.4 (13.2)	39.9 (8.3)	5 (0.03 to 10.1)	.049^e^	0.55
Illness Cognition Questionnaire (ICQ)							
Helplessness	13.2 (4.4)	12.9 (4.3)	11.1 (2.3)	11 (2.6)	–0.2 (–2 to 1.5)	.77	–0.07
Acceptance	14.4 (3.3)	13.6 (3.9)	15.9 (4)	15.5 (3.2)	0.8 (–1.1 to 2.7)	.41	0.22
Perceived benefits	13.7 (3.4)	13.4 (2.8)	16.1 (4)	14.9 (5.1)	–0.7 (–2.6 to 1.2)	.46	–0.17
Care-partner reported questionnaires							
B-ADL^f^	3.3 (2.3)	3.9 (2.2)	3.3 (2.4)	4.2 (2.3)	0.2 (–1.1 to 1.6)	.71	0.11
ECog^g^ questionnaire							
ECog	2.4 (0.7)	2.6 (0.6)	2.2 (0.6)	2.5 (0.8)	–0.0 (–0.4 to 0.4)	.99	0.00
Memory^h^	3 (0.7)	3 (0.6)	2.7 (0.7)	3 (0.8)	0.2 (–0.2 to 0.6)	.27	0.29
Language	2.3 (0.9)	2.5 (0.8)	2.2 (0.8)	2.3 (0.9)	–0.1 (–0.6 to 0.4)	.76	–0.09
Visuospatial	1.8 (0.8)	2.1 (0.8)	1.8 (0.6)	2.1 (0.8)	–0.1 (–0.6 to 0.4)	.61	–0.16
Planning	2.1 (0.9)	2.3 (0.9)	1.9 (0.8)	2.4 (0.9)	0.3 (–0.3 to 0.8)	.31	0.31
Organization	2.5 (0.9)	2.6 (0.8)	1.9 (0.7)	2.1 (0.7)	–0.1 (–0.5 to 0.4)	.79	−0.07
Attention	2.5 (0.8)	2.8 (1)	2.2 (0.9)	2.5 (0.9)	–0.1 (–0.6 to 0.5)	.78	−0.08
Zarit Burden Scale	28.1 (17.7)	28.9 (19.2)	26.2 (14.2)	27 (17.7)	0.3 (–6.6 to 7.3)	.92	0.02

a Control=usual care+weekly emails on cognition and memory.

b DVA=reablement goal-setting+personalized program via the Amazon Alexa Show 8.

c Adjusted for baseline.

d GDS: Geriatric Depression Scale.

e Indicates a significant difference in change between groups (*P*<.05).

f B-ADL: Bayer Activities of Daily Living.

g ECog: Everyday Cognition scale.

h Analysis was conducted on natural log–transformed data due to nonnormal distribution.

## Discussion

### Principal Findings

Findings from this pilot RCT demonstrate the feasibility of a co-designed cognitive reablement program broadcast via DVAs to support older adults living with dementia/MCI. The target sample was successfully recruited, and a high retention rate was achieved. Adherence, assessed as weekly interaction with program goals, exceeded target levels (66%), and the mean SUS score indicated marginal acceptability. Secondary outcomes showed the intervention group participants achieved a significant improvement in the attainment of personalized reablement goals. While most outcomes did not differ between groups, memory strategy use was higher after 12 weeks in the intervention participants compared to the control group. Given that this feasibility study was not designed or powered to evaluate efficacy, a full-scale trial is required to evaluate the impact for people living with dementia/MCI.

### Feasibility

Limited evidence on digital health applications to remotely deliver cognitive reablement programs for people living with dementia/MCI restricts comparisons of our study results [57-59]. Previous studies using smartphones or tablet-based apps for cognitive stimulation or training in people living with dementia have reported recruitment challenges related to device incompatibility [60,61]. The provision of the low-cost DVA, currently retailing at Aus $349 (Aus $1=US $0.70 as of Oct 8, 2026), as an additional device may, in part, explain our higher (30/35, 86%) recruitment rate. The intervention group retention rate of 93% (14/15) is higher than in previous telehealth reablement studies that observed retention rates of 60% [16] and 83% [17]. Careful interpretation is required given the significant difference in participant time burden for the virtual face-to-face studies. The 70% adherence rate was lower than 80% [28] and 85% [27] rates recorded in previous BuddyLink studies; however, this result should be interpreted with caution given personalization of VOICE-COG resulted in participants nominating between 7 to 52 weekly prompts compared to 1 or 2 in previous programs. A qualitative study with care partners of people living with dementia (n=5) explored the feasibility of DVA use for individual cognitive stimulation therapy (iCST), finding device accessibility, training provision, and visual stimulation could increase engagement [24]. The moderately high adherence rate in our study may have been due to the co-designed nature of the VOICE-COG program, which improved these functions and features through iterative feedback. Together, our feasibility findings provide support for a future large-scale trial.

### System Usability

The VOICE-COG program’s usability was rated as marginally acceptable (mean SUS 56.3, SD 18.1). It is possible this result was limited by the SUS outcome measure, which has not been fully validated for use in this population or with the DVA modality. The SUS is commonly used in technology evaluations by people living with dementia/MCI [62]; however, a modified version with simplified language has been proposed [63]. Furthermore, a recent review highlighted that usability assessments designed for graphical user interfaces need to be revised and validated for application with DVA systems [64]. The Voice Usability Scale could be considered as an outcome measure for future trials, having been tested as an alternative to the SUS on the Amazon Alexa [65]. Finally, in alignment with the IDEAS framework, a subsequent qualitative analysis of user experiences will be conducted to inform further development of VOICE-COG for evaluation in large-scale RCT. Future research should consider exploratory analyses examining associations between usability and other outcome measures to determine whether differences in usability for each VOICE-COG program goal influence effectiveness.

### Goal Attainment

Goal attainment significantly increased among participants who received the VOICE-COG reablement program via their DVAs, with 60% (9/15) reporting having met or exceeded their goals. It is possible these results were influenced by the goal-setting behavior change process alone. While GAS-Light has recently been recommended for goal setting in reablement programs for people living with dementia [35], the heterogeneity of goal outcome measures used in previous studies limits comparability [66,67]. Furthermore, there is a lack of standardization in the application of the GAS with differences in scales and/or allocation of baseline scores [66]. A study using a touchscreen prompter to support household tasks (only) in people living with dementia (n=8) found 64% (14/22) of goals were met after 4 weeks [68], consistent with our finding of 68% (25/37) of goals achieved. More frequent use of the touchscreen prompter was associated with successful achievement of goals [69], highlighting future research should explore the relationship between adherence and goal attainment. The virtual telehealth study reported a higher percentage of goals were achieved (7/9, 78%) in the telehealth intervention group; however, the smaller sample size (n=3) limits comparison [16].

### Cognitive Outcomes

Intervention participants in our study reported significantly higher use of memory strategies after 12 weeks compared with the control group. Despite this promising result, longer duration and adequately powered evaluation trials are required to determine if the increased use of DVA memory strategies could have a positive influence on memory performance and satisfaction over time [70]. It could be argued that this positive result was due to the integrated DVA reminder functions, which include alarms, timers, lists, calendars, and routines, which have been reported to support older adults with memory [71]. Future research should explore the effect of the VOICE-COG program that includes prompts and training support for the integrated functions compared to standalone DVA use. There is limited evidence on the effects of assistive technology for memory support in people living with dementia, with 0 studies included in a 2017 Cochrane review [72]. A recent smartphone study found people living with dementia (n=52) who used a voice recorder or reminder app reported improvements in prospective memory after 4 weeks, with higher levels of app engagement associated with a significant (*P*=.049) improvement in Instrument of Activities of Daily Living (IADL) [60]. Given the borderline statistical significance in both studies, additional research is needed to determine if there are meaningful effects on cognition and functioning for people living with dementia/MCI longer term.

### Implications for Future Research

Feasibility results from this study support consideration of a full-scale trial to evaluate the impact of VOICE-COG, relative to usual care for people living with dementia/MCI. The effect sizes found in this study may be used to inform decision-making regarding adjustments, such as sample size for a future trial [73]. Recruitment via clinically facilitated partnerships, such as memory clinics, should be considered to reduce selection bias from web-based advertisements. VOICE-COG used co-design to adapt a practical cognitive reablement framework for people living with dementia [74] and a theory-based goal-focused approach [75,76], drawing on various evidence of empirically supported strategies. Future research should examine reasons for lack of goal attainment unrelated to usability and ensure the latest reablement strategies for older adults with dementia are incorporated. This should include increasing accessibility of VOICE-COG to a more diverse population, including minority ethnic groups, or lower socioeconomic status, and those in rural areas. Digital competency measures should be collected at baseline in future trials to investigate potential bias and determine if this influences intervention engagement. Given the high prevalence of depression and anxiety in this population, consideration should be given to the use of positive psychology outcome measures currently in development [77,78]. An important issue for future research is consensus on an objective validated measure of adherence or engagement for digitally delivered reablement support programs and DVA interventions. Finally, the cost-effectiveness of VOICE-COG should be evaluated in comparison to a telehealth virtual reablement program before implementation at scale.

### Limitations

Recruitment methods in this study used web-based applications that required a level of digital literacy, which could limit generalization of these findings to all people living with dementia/MCI. This may have led to an unintended bias of a highly educated sample, potentially limiting generalization to all people living with dementia/MCI. Since participants self-reported their diagnosis of dementia/MCI, cognitive impairment stage could not be determined. Therefore, the inclusion of an objective, validated cognitive assessment should be considered for future studies. Goal attainment measures were self-reported and could have been positively influenced by social desirability bias. Furthermore, control group participants did not set reablement goals, so we could not ascertain if goal attainment differences were associated with the intervention or the goal-setting process. Participants had not previously owned an Amazon Alexa Show 8 DVA, so SUS responses may have been influenced by the DVA’s integrated features and functionality. Questionnaires were administered online, but several people living with dementia shared email addresses with their care partners, so responses could have been misrepresented due to proxy reporting bias. Secondary outcomes should be interpreted with caution as our sample size was not powered to detect effects. Consistent with the exploratory aims of feasibility studies, we did not correct for multiple comparisons in hypothesis testing, which may have led to false-positive findings. Finally, as a pilot feasibility trial of a minimum viable product, additional human support elements included researcher input during program configuration. Future trials should consider an integrated configuration interface, enabling direct transfer of data from web-based forms into the BuddyLink software for real-world implementation.

### Conclusions

This co-designed VOICE-COG cognitive reablement program was feasible to deliver to older adults living with dementia/MCI via DVA, with high levels of retention and adherence. Larger trials with longer-term follow-up and cost-effectiveness evaluation are needed to determine how this remotely delivered and monitored program can support older adults living with early-stage dementia or MCI to meet their reablement goals.

## Supplementary material

10.2196/96922 Multimedia Appendix 1 VOICE-COG program information for participants.

10.2196/96922 Multimedia Appendix 2 Example VOICE-COG program goals.

10.2196/96922 Multimedia Appendix 3 VOICE-COG program detail.

10.2196/96922 Multimedia Appendix 4 Weekly VOICE-COG broadcasts and interactions by participant.

10.2196/96922 Multimedia Appendix 5 System Usability Scale.

10.2196/96922 Checklist 1 CONSORT-EHEALTH (V 1.6.1) checklist.

## Abbreviations

B-ADL: Bayer Activities of Daily Living Scale
CONSORT-EHEALTH: Consolidated Standards of Reporting Trials of Electronic and Mobile Health Applications and Online Telehealth
COPE: Care of People with dementia in their Environments
DVA: digital voice assistant
ECog: Everyday Cognition
GAS: Goal Attainment Scale
GDS: Geriatric Depression Scale
IADL: Instrument of Activities of Daily Living
ICQ: Illness Cognition Questionnaire
iCST: individual cognitive stimulation therapy
IDEAS: Integrate, Design, Assess, and Share
MCI: mild cognitive impairment
MMQ: Multifactorial Memory Questionnaire
RCT: randomized controlled trial
SMART: Specific, Measurable, Achievable, Relevant, and Time-bound
SUS: System Usability Scale
