# Supplementary material for: Feasibility of a Co-Designed Personalized Cognitive Reablement Program Delivered via Digital Voice Assistants for Older Adults Living With Early-Stage Dementia or Mild Cognitive Impairment: Pilot Randomized Controlled Trial
Source: JMIR Aging. 2026 Oct 23;9:e96922. doi: 10.2196/96922 (PMC13693962; doi:10.2196/96922)
Supplement: Supplementary file 1 [file aging-v9-e96922-s001.pdf]

VOICE-COG COGNITIVE REABLEMENT PROGRAM

Please find attached details of the Co-designed Cognitive Reablement Program that will be personalised for you. Participants will develop two or three meaningful goals from the program. Using our Buddylink skill, Alexa will broadcast information and instructions based on the personalised goals.

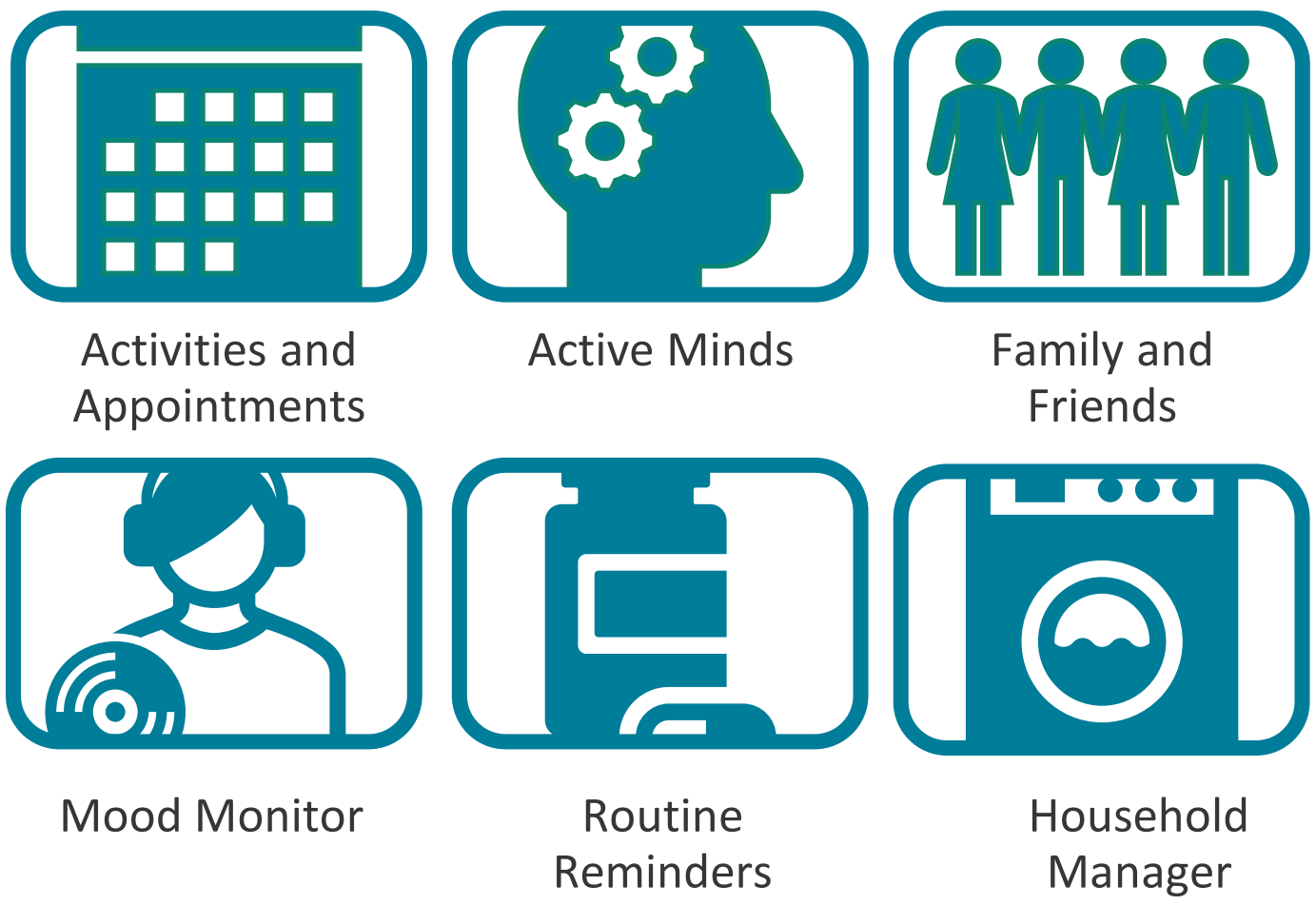

Goal 1. Routine Reminders – to support management of your daily routine, Alexa will provide audio and written prompts for self-care activities such as taking medication, drinking water, and eating meals/snacks.

Goal 2. Mood Monitor - will check-in on how you're feeling and ask you to select from activities to be broadcast on the Alexa including music, completing a breathing/mindfulness exercise, listening to nature sounds or hearing a joke.

Goal 3. Family and Friends Facilitator - will remind you to call family or friends using your voice to make video or mobile phone calls on the Alexa, as well as display online photo albums of family and friends. There will also be an option to complete your Life Story over the 12-weeks which can then be broadcast to on your Alexa device.

Goal 4. Household Manager - will remind you when it's time to do daily or weekly chores including personalised step-by-step prompts on how to use appliances or remind you where items are located.

Goal 5. Activities and Appointment Assistant will use audio and text to announce your calendar appointments daily as well as reminding you when it's time to get ready and when it's time to leave. You can also ask Alexa to read out your Checklists of items to remember to take with you.

Goal 6. Active Minds Adviser - will remind you to engage in one of four audio and/or visual activities on the Alexa each day, from watching the news, listening to an audio book, listening to a podcast, or watching a short documentary video.
