# Supplementary material for: Feasibility of a Co-Designed Personalized Cognitive Reablement Program Delivered via Digital Voice Assistants for Older Adults Living With Early-Stage Dementia or Mild Cognitive Impairment: Pilot Randomized Controlled Trial
Source: JMIR Aging. 2026 Oct 23;9:e96922. doi: 10.2196/96922 (PMC13693962; doi:10.2196/96922)
Supplement: Supplementary file 2 [file aging-v9-e96922-s002.pdf]

Appendix 3: Examples of Personalized VOICECOG Program Goals

VOICECOG Goals Set (N=39)	Participant Examples:	
	Current Functioning	Personalized Goal
Activities & Appointments (n=12)	Missing medical appointments despite looking at calendar<br>Stopped attending lawn bowls due to injuries and social issues.	With the support of VOICE-COG, I will return to lawn bowls every Saturday remembering items I need to take with me.<br>VOICE-COG will also remind me when and how to use Alexa's voice-controlled calendar function to add or change appointments – with reminders to attend all my medical appointments.
Mood Monitor (n=8)	Participant's anxiety has returned because of being in aged care as it can be distressing hearing other participants with advanced dementia in the evening.	VOICE-COG will broadcast 'mood monitor' reminders every morning to help me reduce my anxiety and stress by doing a mindfulness exercise or listening to country and western music from my childhood.
Family and Friends (n=7)	Starting to have difficulty remembering early life and young adult memories. The participant has difficulty remembering grandchildren and great grandchildren's names.	VOICE-COG reminders will help remind me to write a chapter of my life story each week and then teach me how to watch my life story on Alexa to also share with family and friends.
Routine Reminders (n=6)	The participant is unable to remember which medication to take when and is reliant on care partner.	VOICE-COG will remind me when to take my medication with personalized images announcing the day and time before each reminder.
Active Minds (n=4)	Due to Posterior Cortical Atrophy dementia symptoms, participant struggles to read.	With VOICE-COGs reminder prompts on when and how to use Alexa's integrated Audible function, I will listen to an audiobook 4 evenings a week for enjoyment and relaxation.
Household Manager (n=1)	Participant relies on their care partner to remind them when to do the laundry and when to recharge phones.	VOICE-COG reminders will help me remember to do the laundry every week and ensure my mobile phone is recharged every day.

Kitchen Helper<br>(n=1)	The participant has difficulty recalling ingredients, including the amounts required and remembering if they've been added.	I will use VOICE-COG to help me bake some of my favorite recipes again, e.g. Christmas Cookies.
