# Supplementary material for: Feasibility of a Co-Designed Personalized Cognitive Reablement Program Delivered via Digital Voice Assistants for Older Adults Living With Early-Stage Dementia or Mild Cognitive Impairment: Pilot Randomized Controlled Trial
Source: JMIR Aging. 2026 Oct 23;9:e96922. doi: 10.2196/96922 (PMC13693962; doi:10.2196/96922)
Supplement: Supplementary file 3 [file aging-v9-e96922-s003.pdf]

Active Minds

Example Goal: I will use the Alexa Active Minds Program to do one of the following each day: i) listen to the news; ii) listen to a podcast; iii) listen to an audiobook; iv) watch a short video ….*(related to my interest in gardening/travel/art/history/other)*.

Good Morning [CLIENTFIRSTNAME],
This is your Active Minds Adviser.
What activity can I help you with today?
Please say,
I want an audiobook, or ,
I want a podcast, or,
I want the News, or,
I want a Video

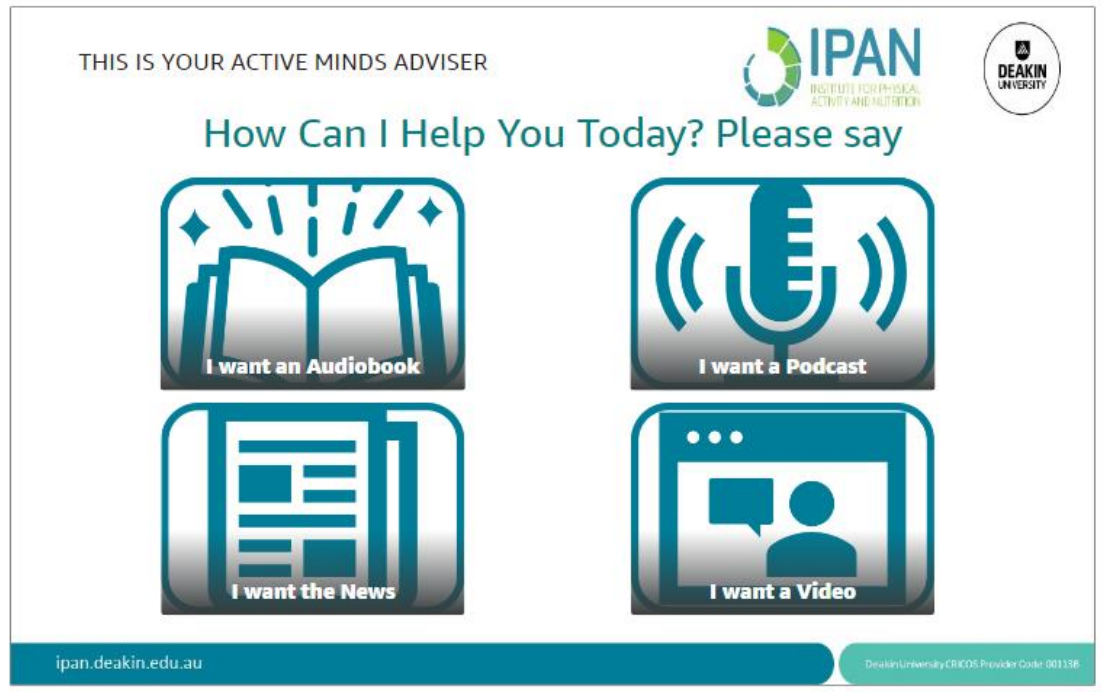

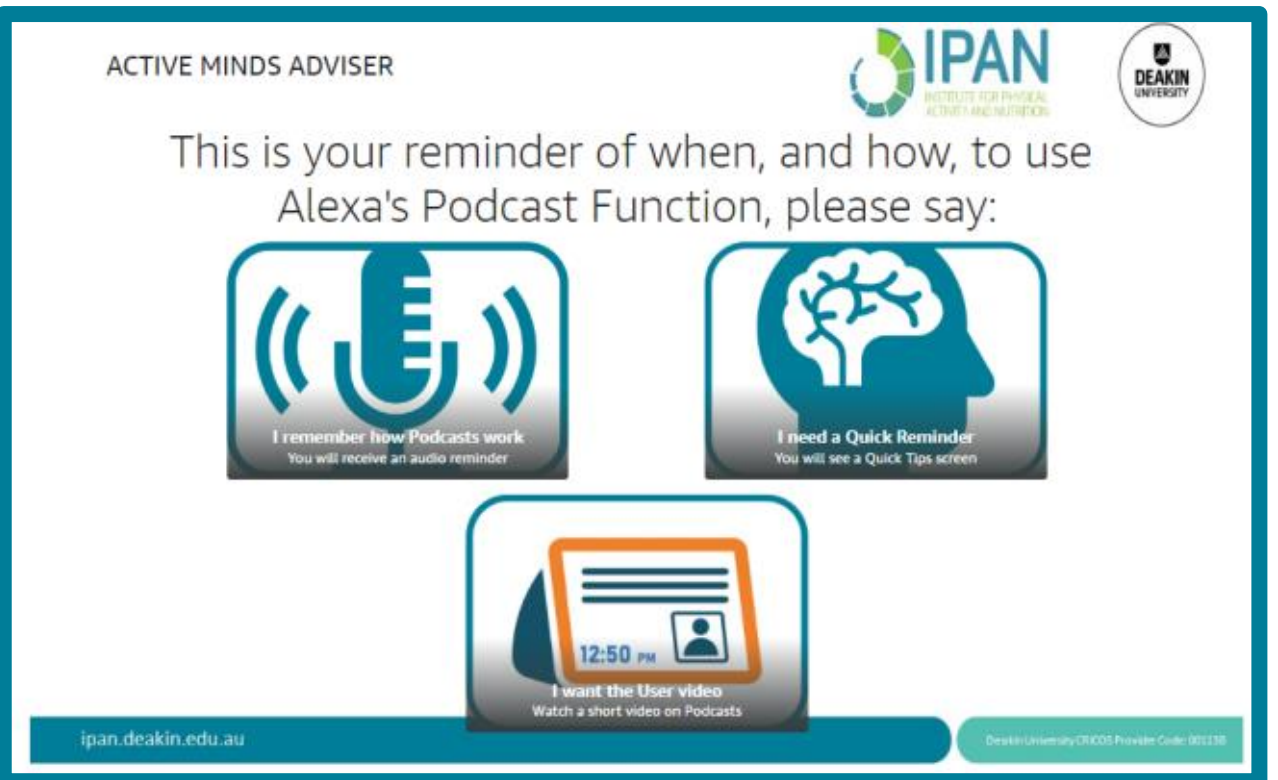

Reminds users how to use audiobooks

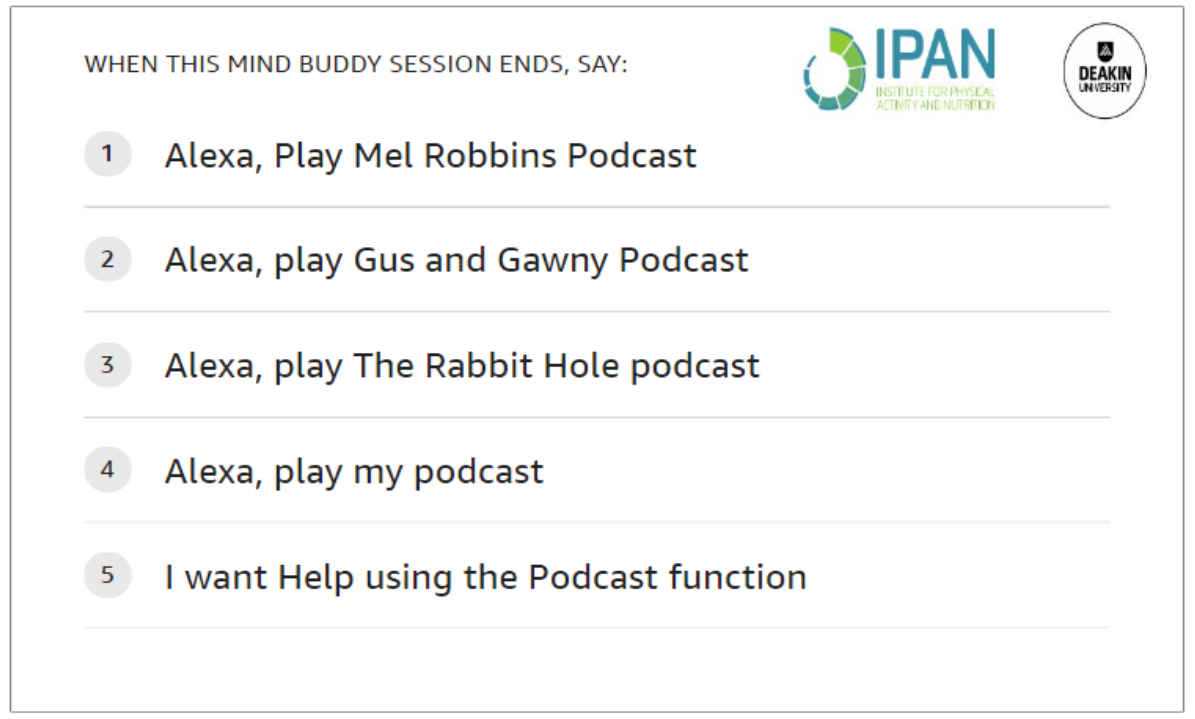

Podcasts (and prompts) personalised

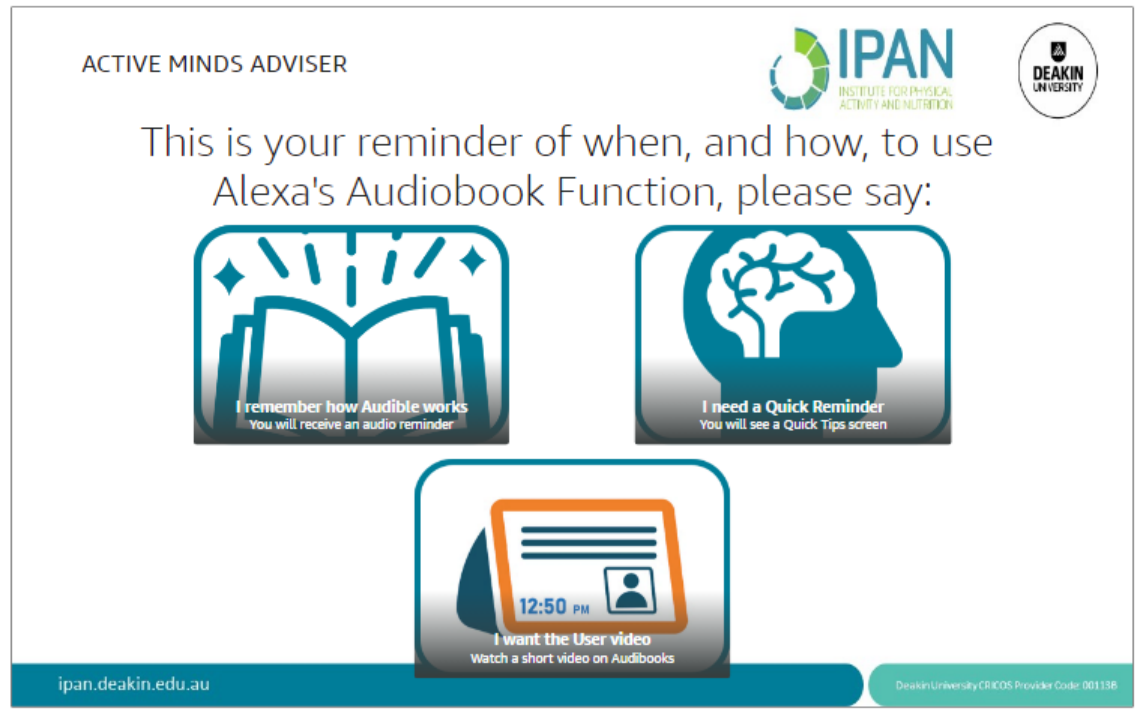

Reminds users *how* to listen to podcasts

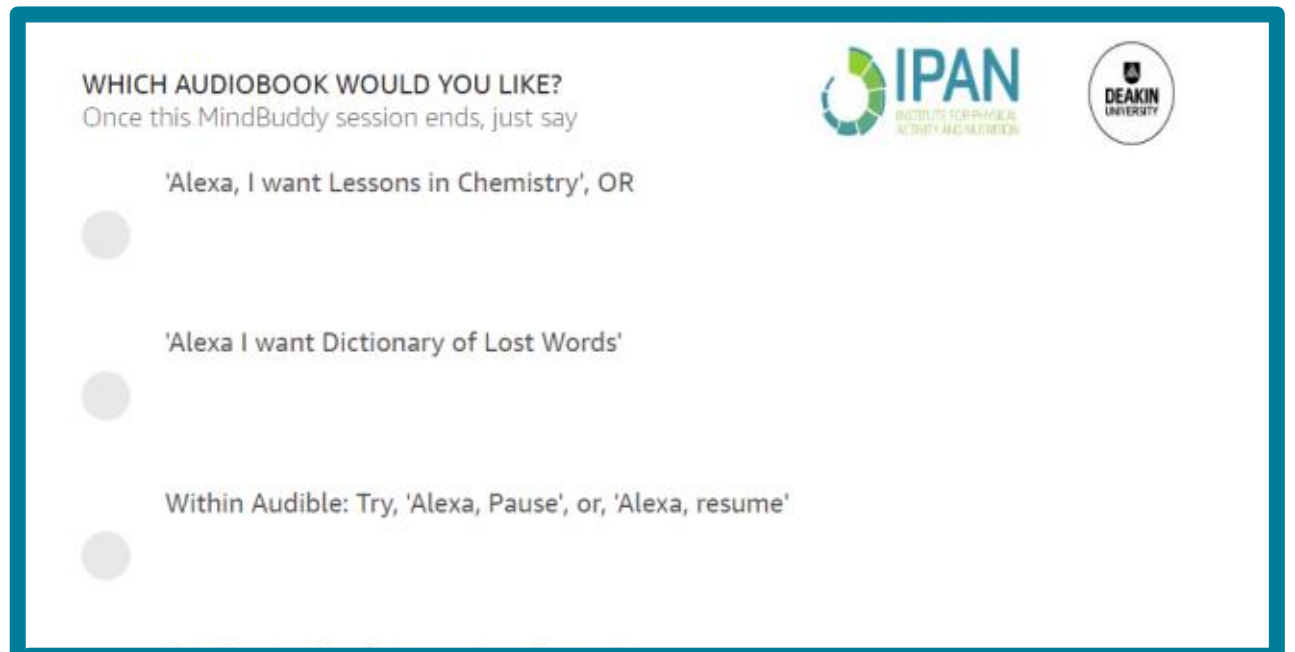

Audiobooks (and prompts) personalised

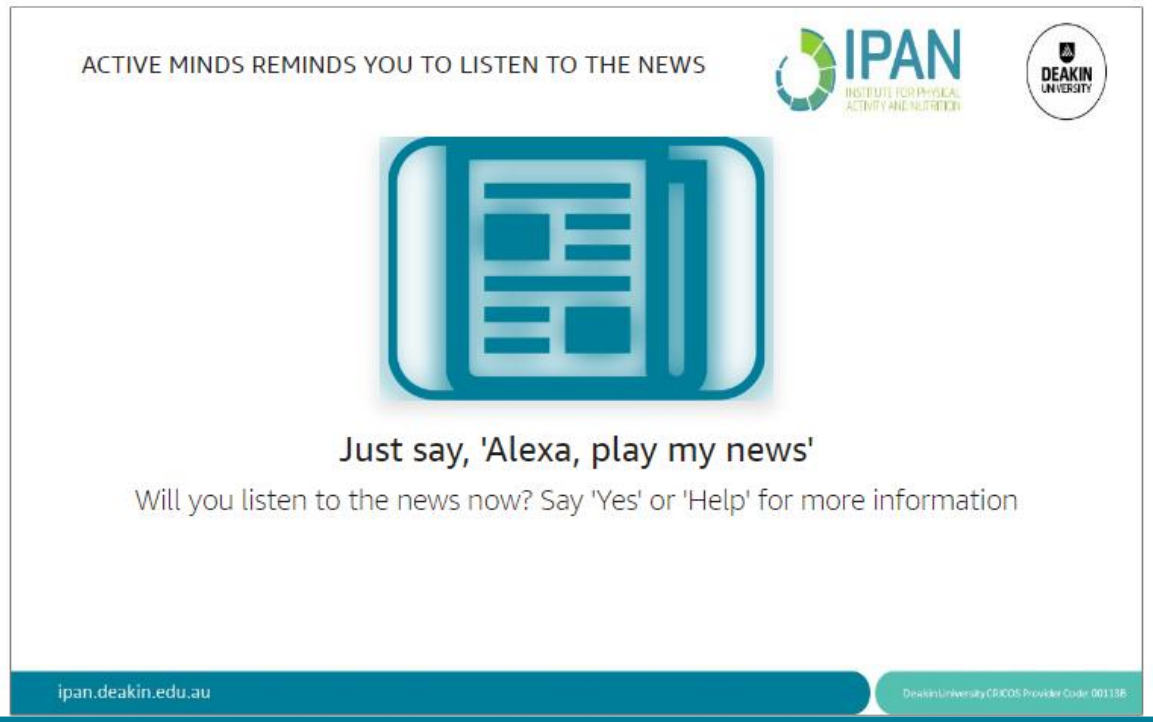

Preferred News Channel set-up

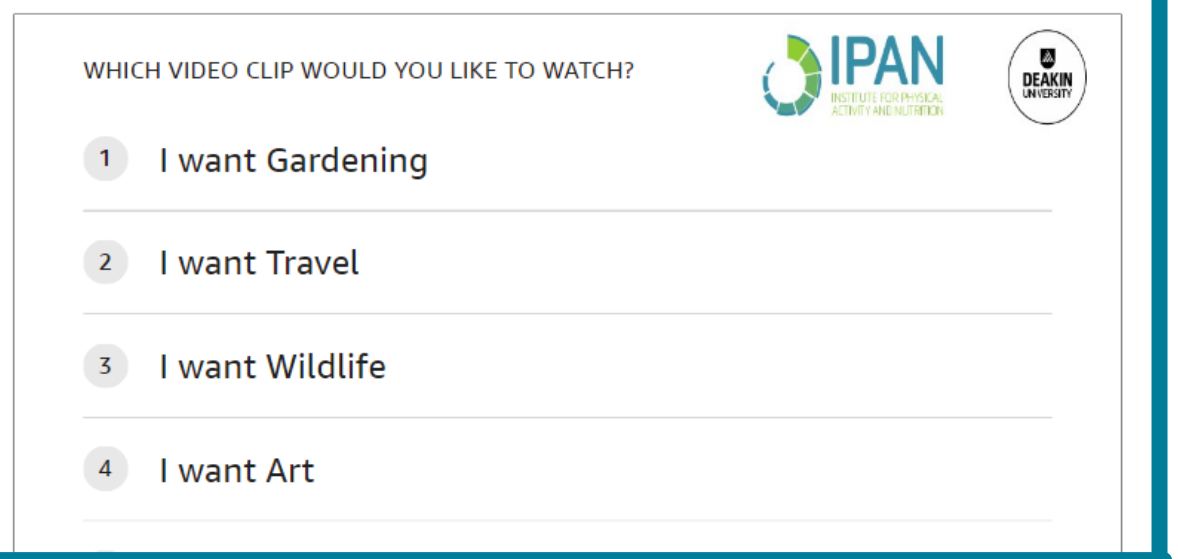

Participants re-directed to a different video each week

Redirects to a pre-selected short online video

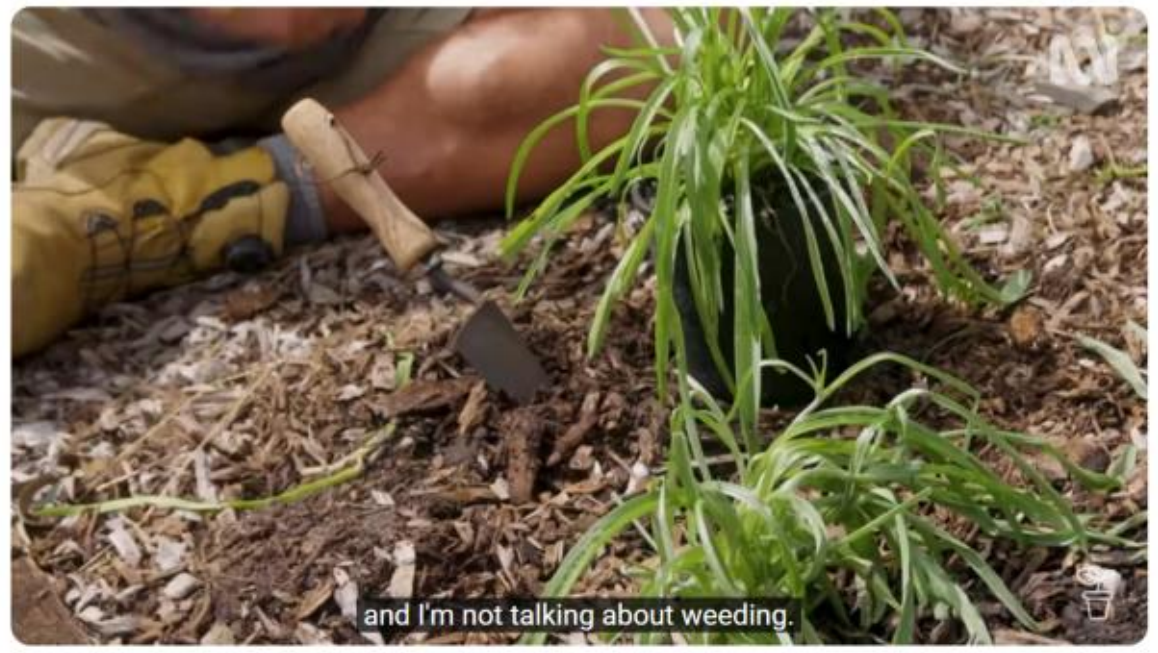

Mood Monitor

Example Goal: I will use the Mood Monitor daily to relax by choosing to i)listen to music, or ii) practising a mindfulness exercise, or iii) watch a nature scene or iv) listen (and maybe learn!) a joke

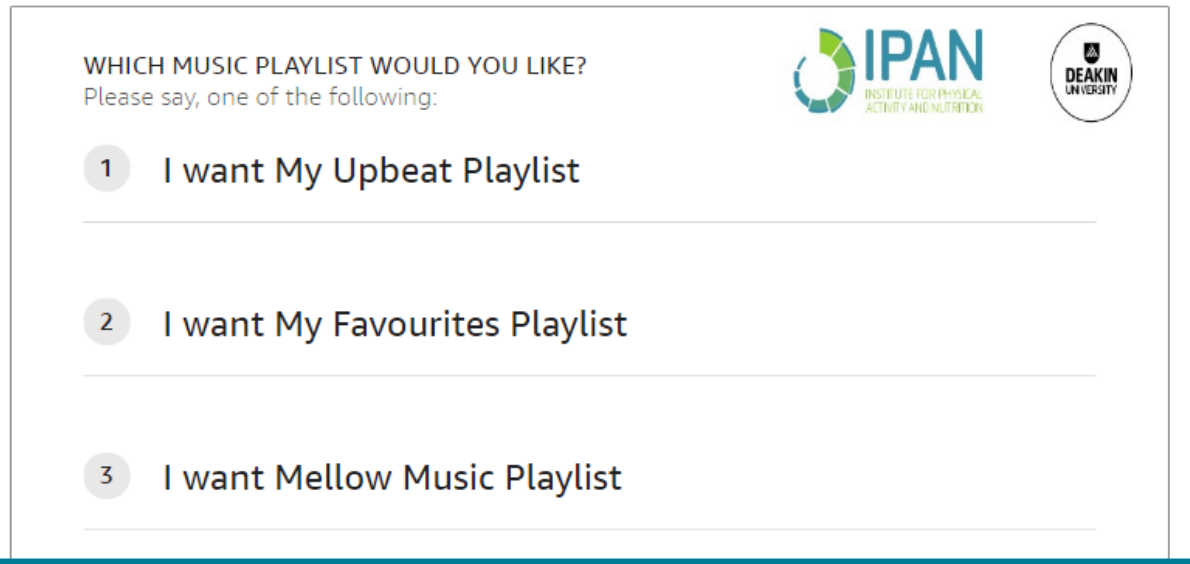

Music playlists will be personalised to participants preferences

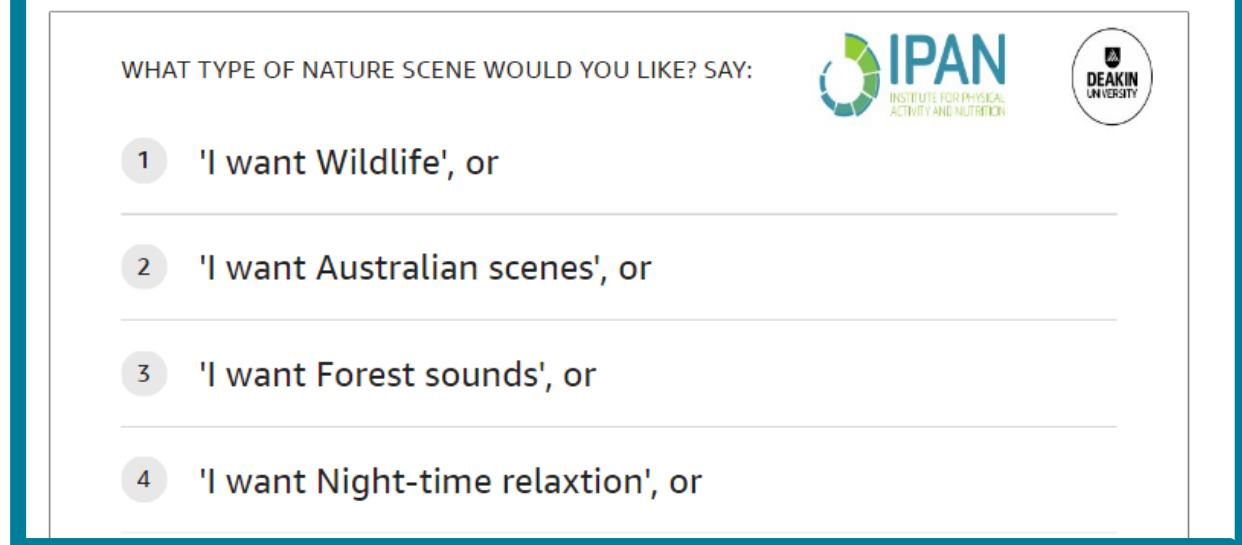

Participants can select relaxing online nature videos

Redirects to an online video

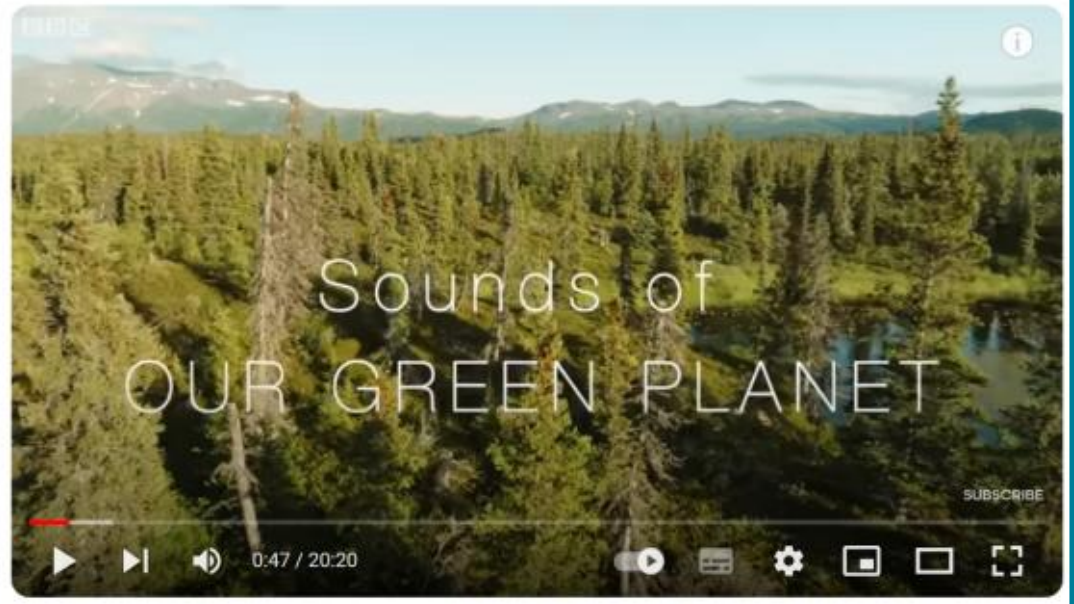

Random selection from pre-defined online videos

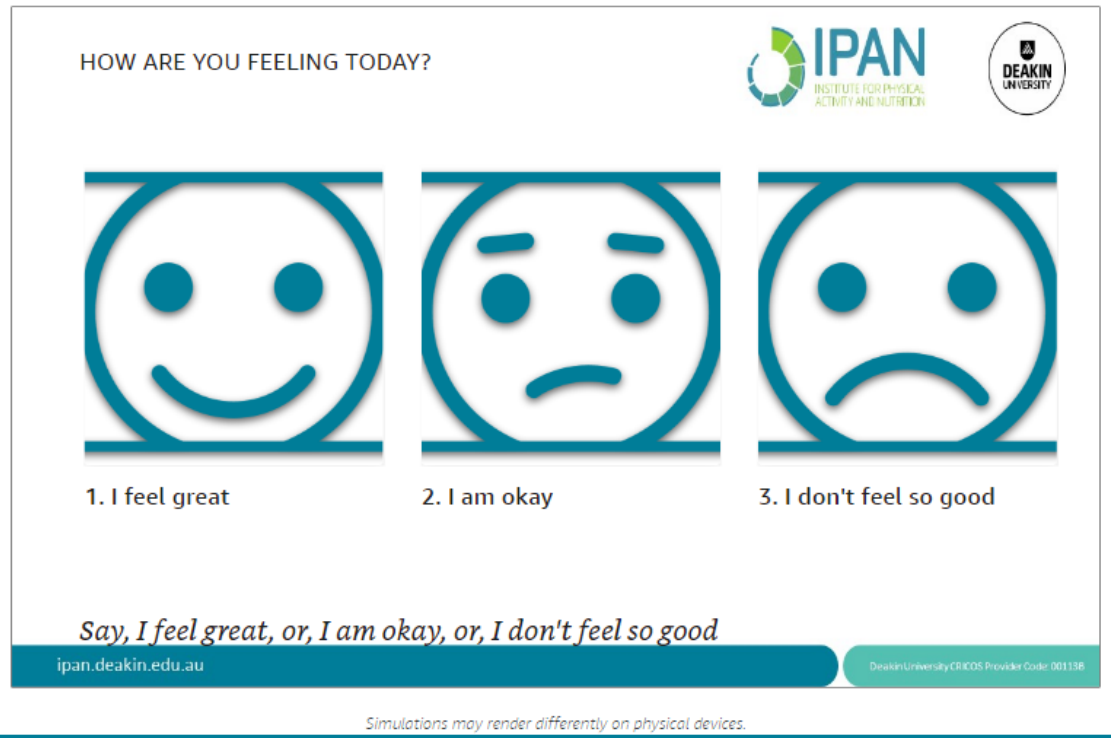

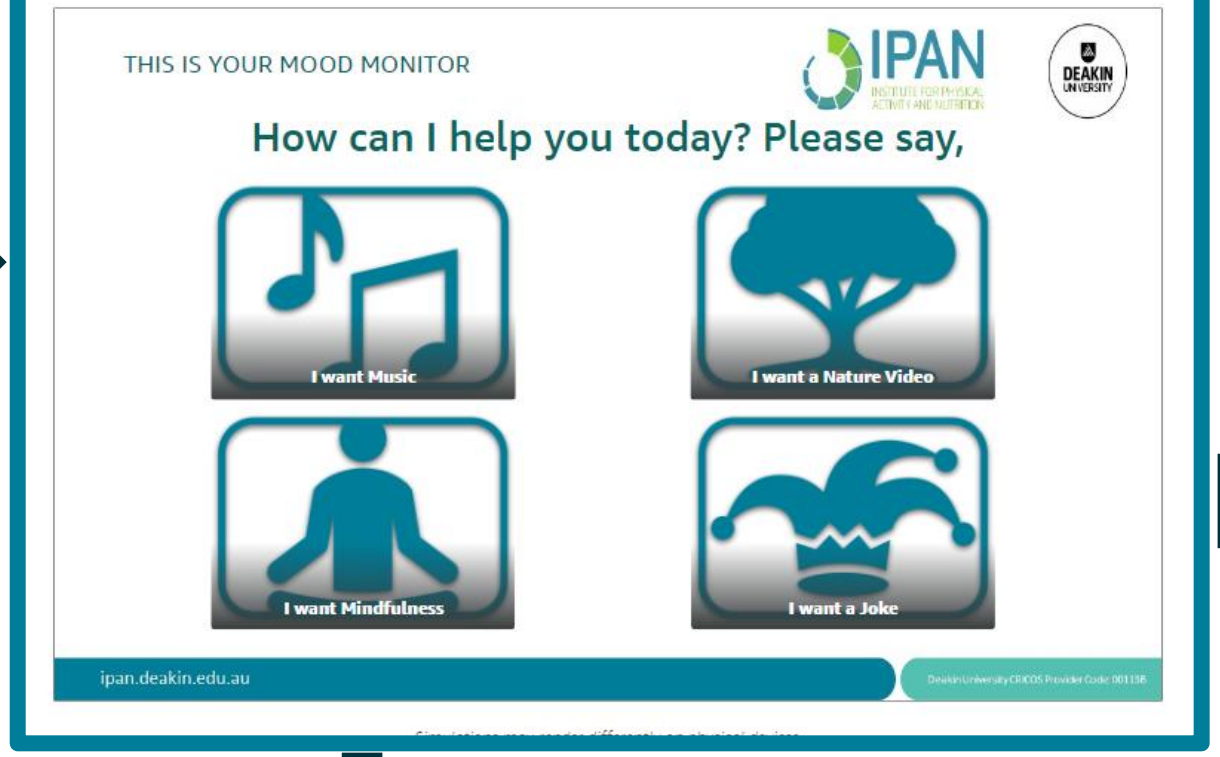

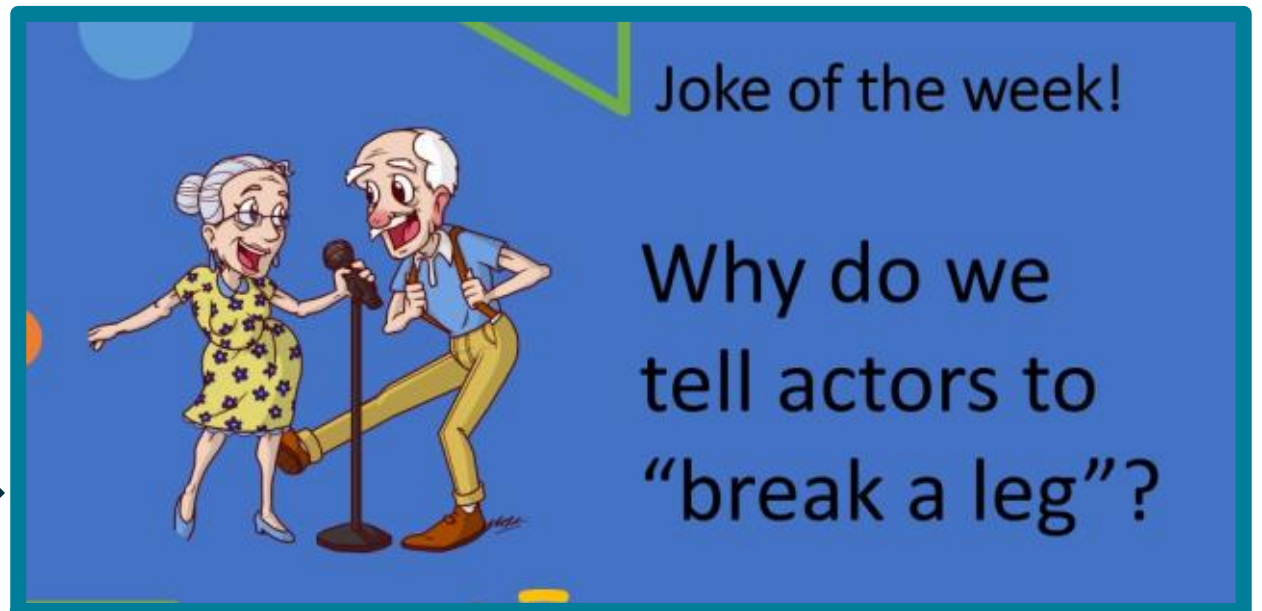

Participants hear the "Joke of the Week"

Good Morning [CLIENTFIRSTNAME],
This is your Mood Monitor.
How are you feeling today?
Please say,
I feel great, or,
I am ok, or ,
I don't feel so good

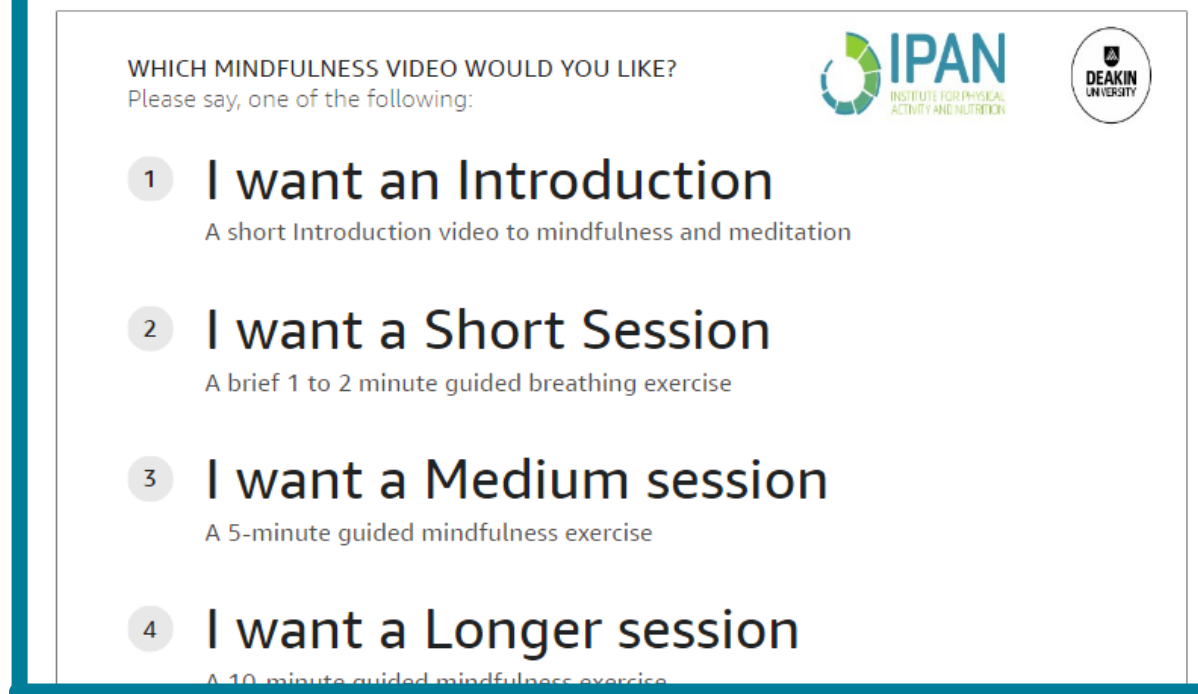

Participants select mindfulness session

Redirects to an online video

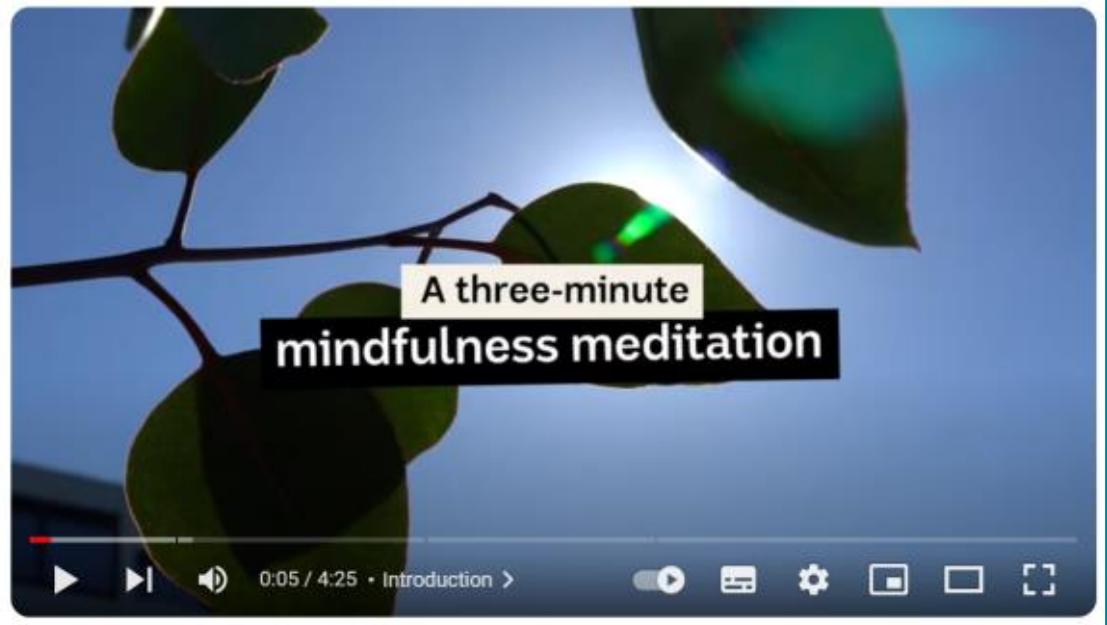

Random selection from pre-defined list of online videos

Routine Reminders

Example Goal: I will take my medicine every day and/or drink 6 glasses of water each day and/or have a healthy snack each morning and afternoon such as fruit and nuts.

Participants will complete details of goals related to self-care activities, such as medication or hydration.

Which morning activities you would like to be reminded to complete?

	Activity	What can Alexa remind you of? (e.g. location; suggestions; special instructions)		
	Activity	Note 1	Note 2	Note 3
Medication	☐			
Breakfast	☐			
Drink	☐			
Other	☐			

Participants can program reminders to be broadcast up to 6 times per day.

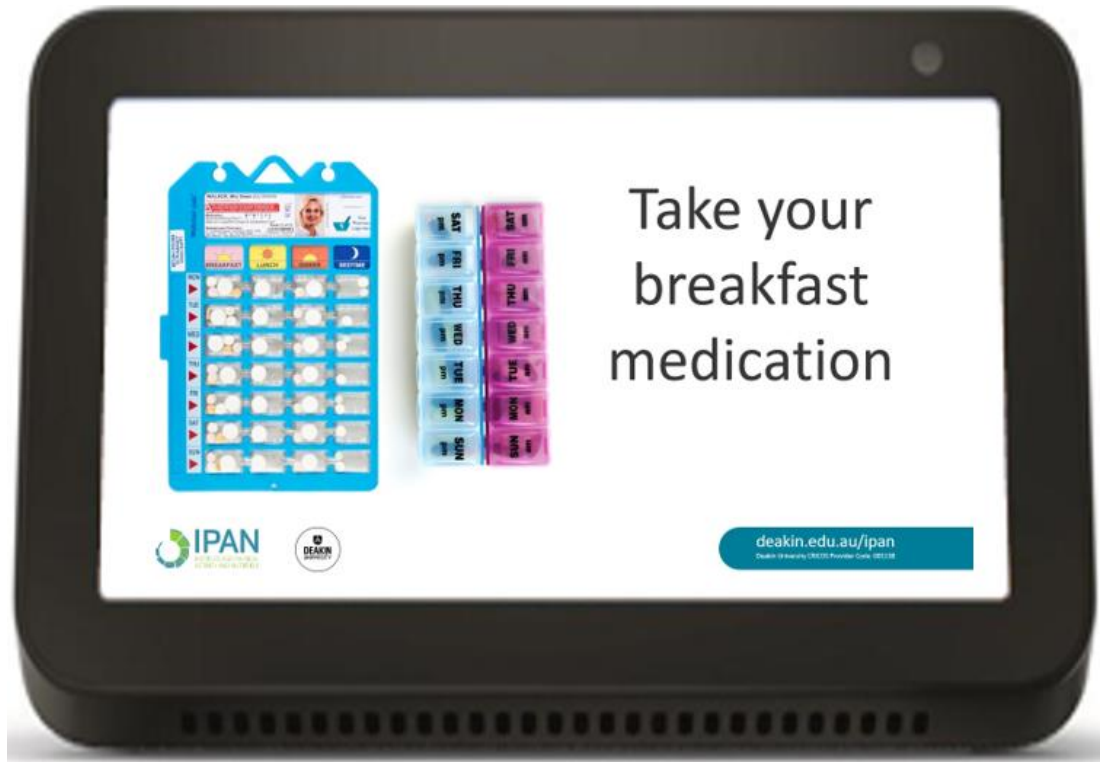

Participants' personal images, or stock images will accompany audio and text reminders.

Glass of Water

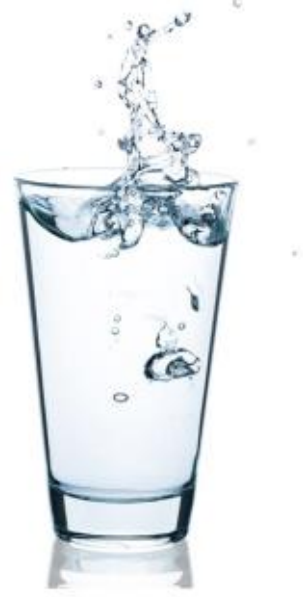

Hello, CLIENTFIRSTNAME,
Did you know regularly drinking water can lubricate your joints and help to prevent pain?
Will you have a glass of water now?

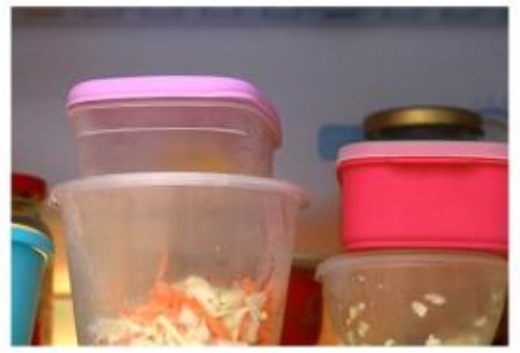

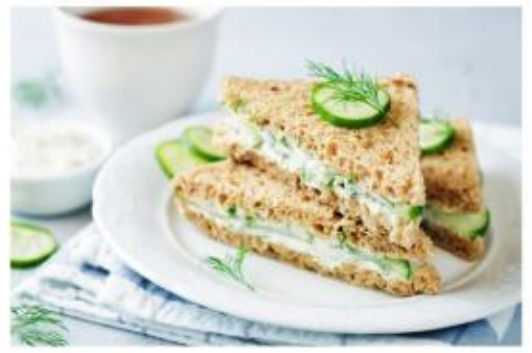

Leftovers

Sandwich

G'day, CLIENTFIRSTNAME,
This is your lunchtime reminder.
Don't forget to check your fridge for leftovers before you make a sandwich!
Will you get your lunch now?

Barriers and facilitators to achieving these goals will be identified with a psychologist. Personalised prompts will then be added to the dialog charts.

Activities and Appointments Assistant

Example Goal: I will attend my exercise class once a week, ensuring I remember my resistance band and water bottle and/or I will attend all my medical appointments and remember to take my glasses, wallet and mobile phone with me.

1) Weekly Activity: Participants will complete details of a weekly leisure or recreation activity goal(s).

Activity 1 Details

	Location	Day	Time (Hour)	Time (Minutes)	AM or PM	When would you like your reminder to get ready?	When would you like your reminder to leave?
Activity 1 details							

Activity 1 Checklist: Please list items to remember before you leave the house (e.g. keys, travel card etc)

Item 1
Item 2
Item 3
Item 4
Item 5

Activity 1: Please upload any photos or short videos related to your activity that would help you when leaving the house

a) Reminder to Get Ready

This is your reminder to get ready for golf.

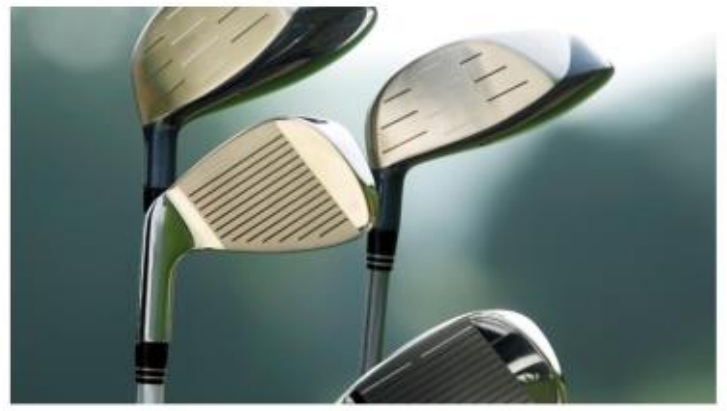

Hello, CLIENTFIRSTNAME,
This is your reminder to get ready for your weekly golf activity.
Are you getting ready for golf now?

b) Reminder to Leave

Resistance Band

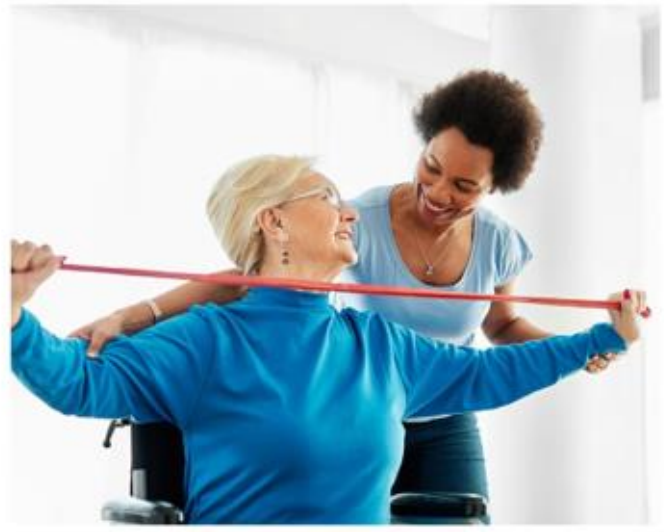

G'day, CLIENTFIRSTNAME,
This is your reminder to leave for you exercise class.
Remember to take your Resistance Band.
Are you leaving for your Exercise now?

2) Ad-hoc appointments: Participants will complete details of appointments.

Please provide us with detail of this week's appointments to add to your calendar.

	Appointment Name	Location	Date	Time (hours)	Time (mins)	Time (AM or PM)
Appointment 1				○	○	○
Appointment 2				○	○	○
Appointment 3				○	○	○
Appointment 4				○	○	○
Appointment 5				○	○	○

Option to link to current digital calendar

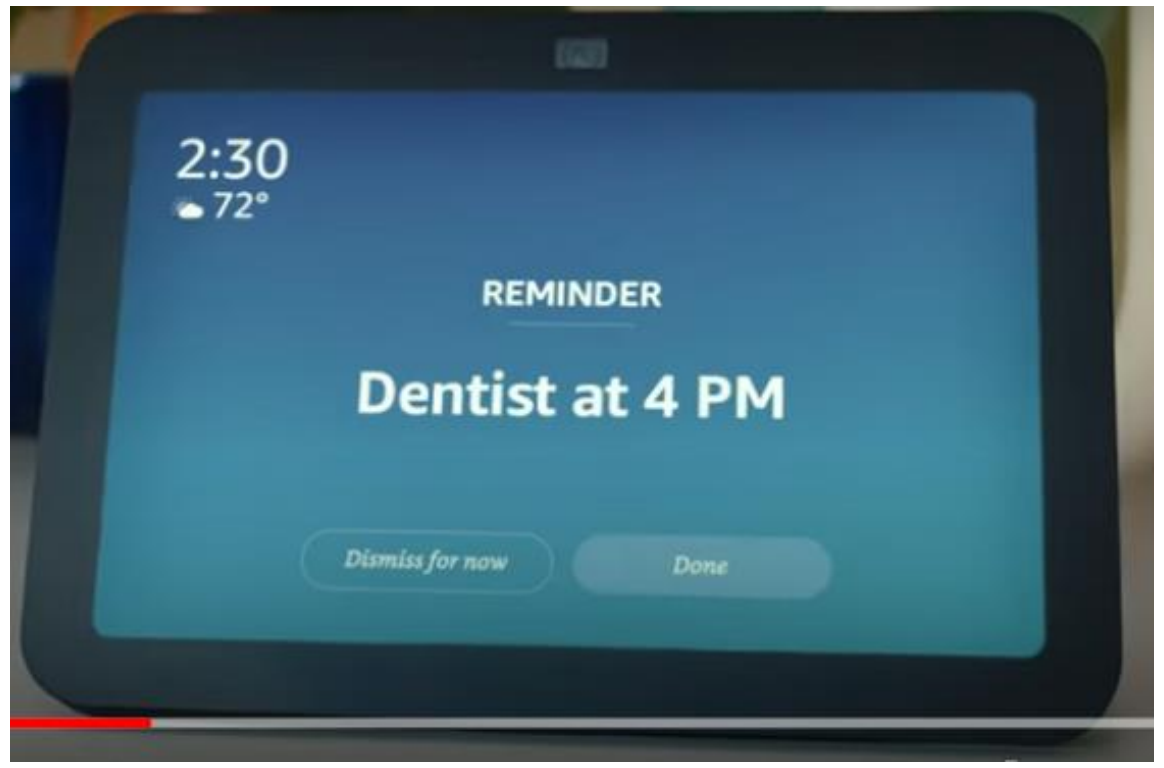

Automated audio and text reminder

Friends and Family Facilitator

Example Goal: I will use Family and Friends to either call a friend or family member every day and/or look at my photo albums and/or view my Life Story.

1

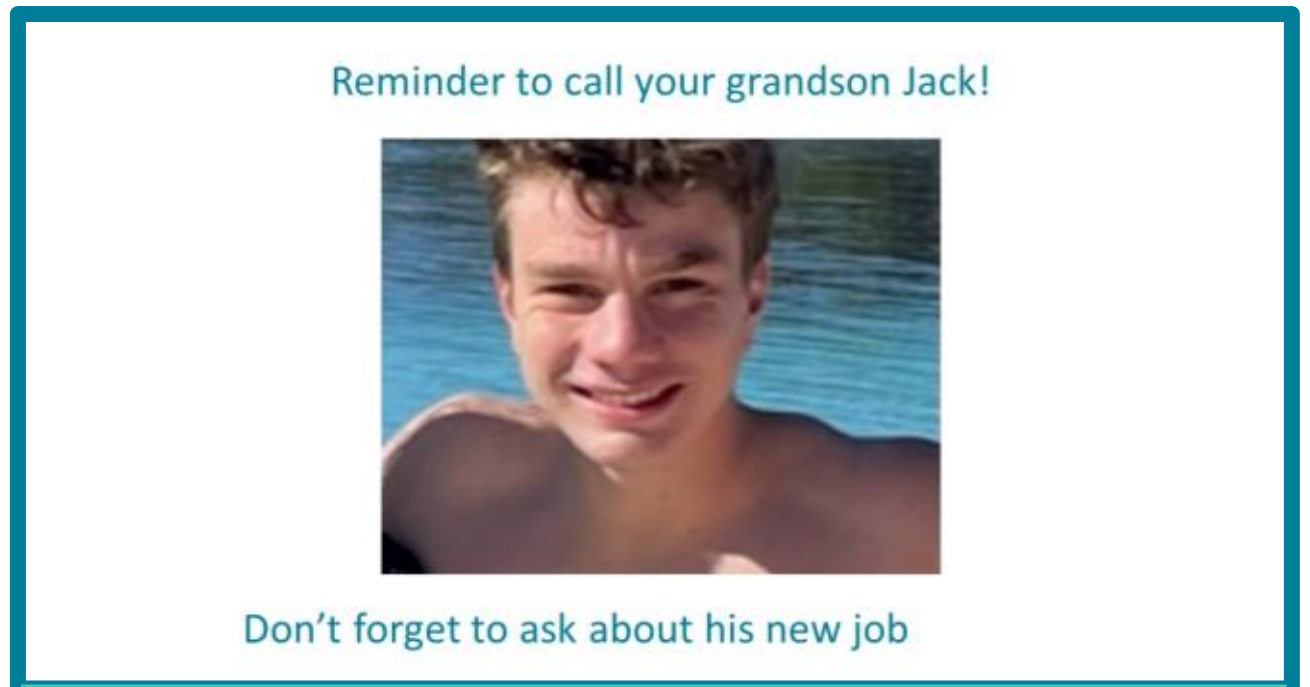

Phone family reminders

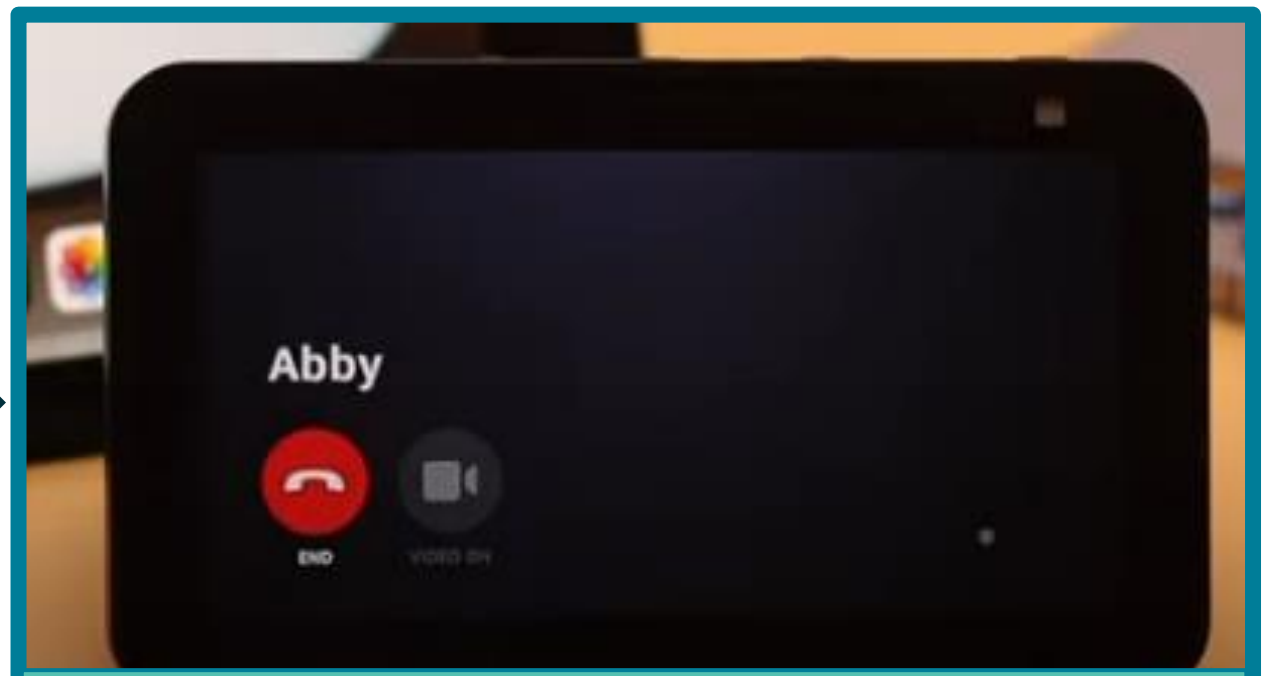

How to make voice-initiated calls

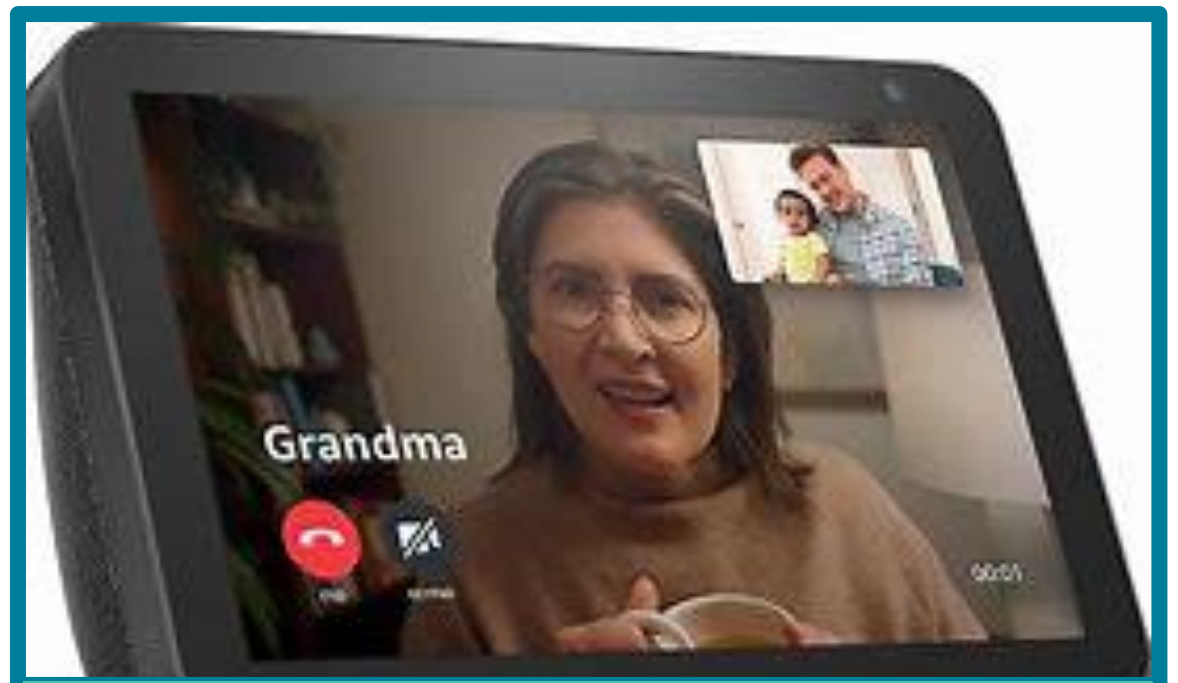

Option for hands-free video calls

2

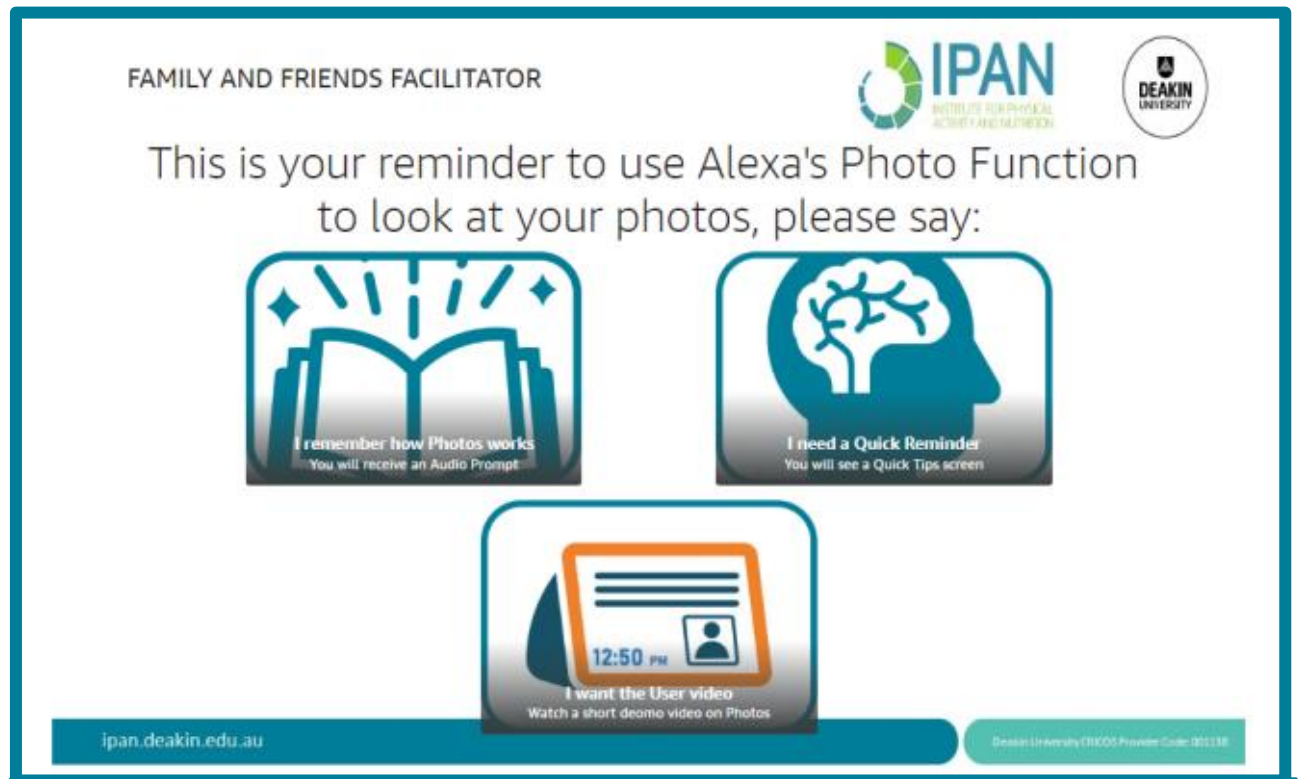

Reminders to browse photos

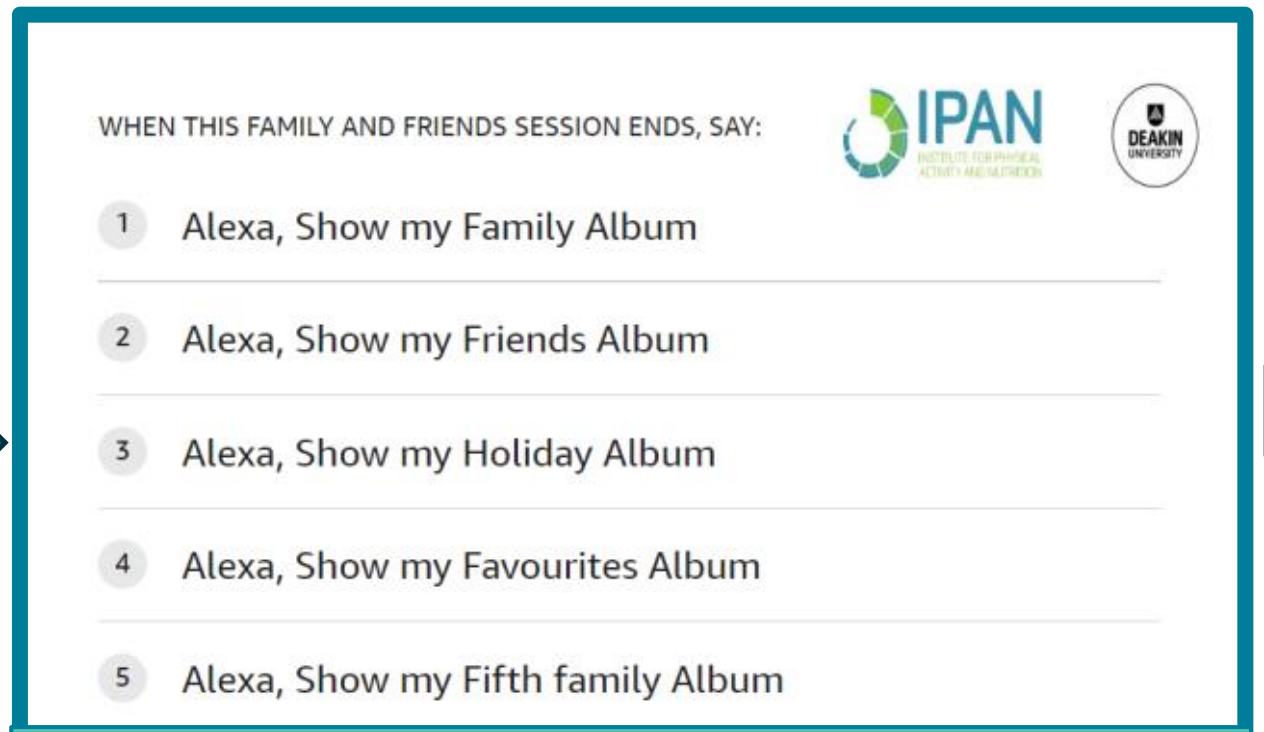

How to view photos

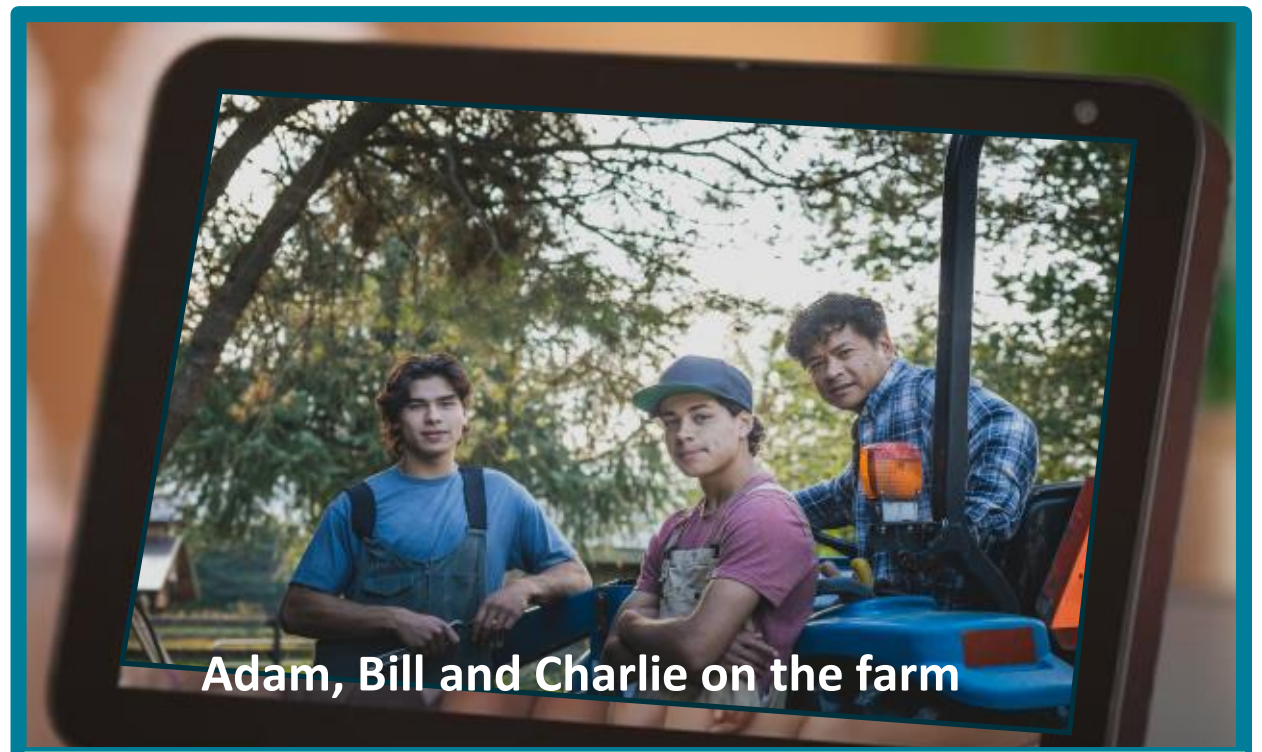

Participant photos with personalised text

3

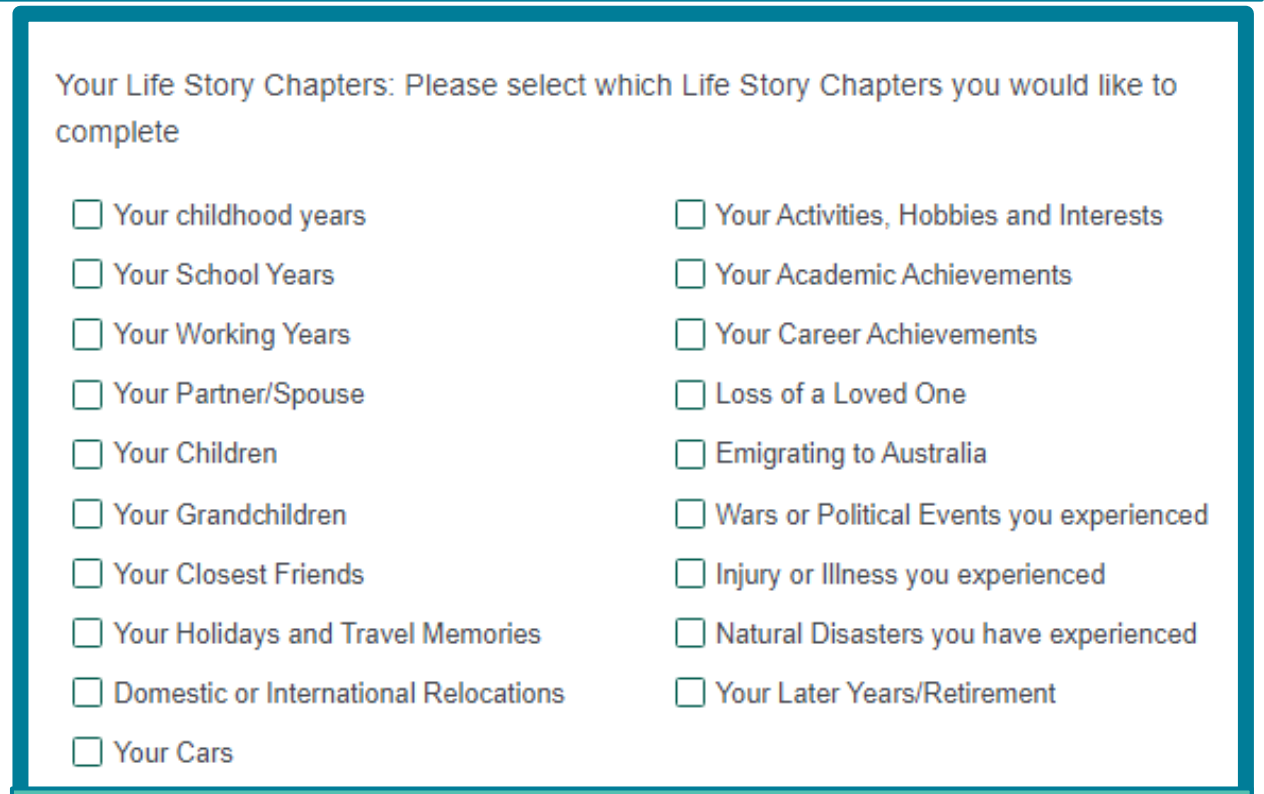

Life Story Template

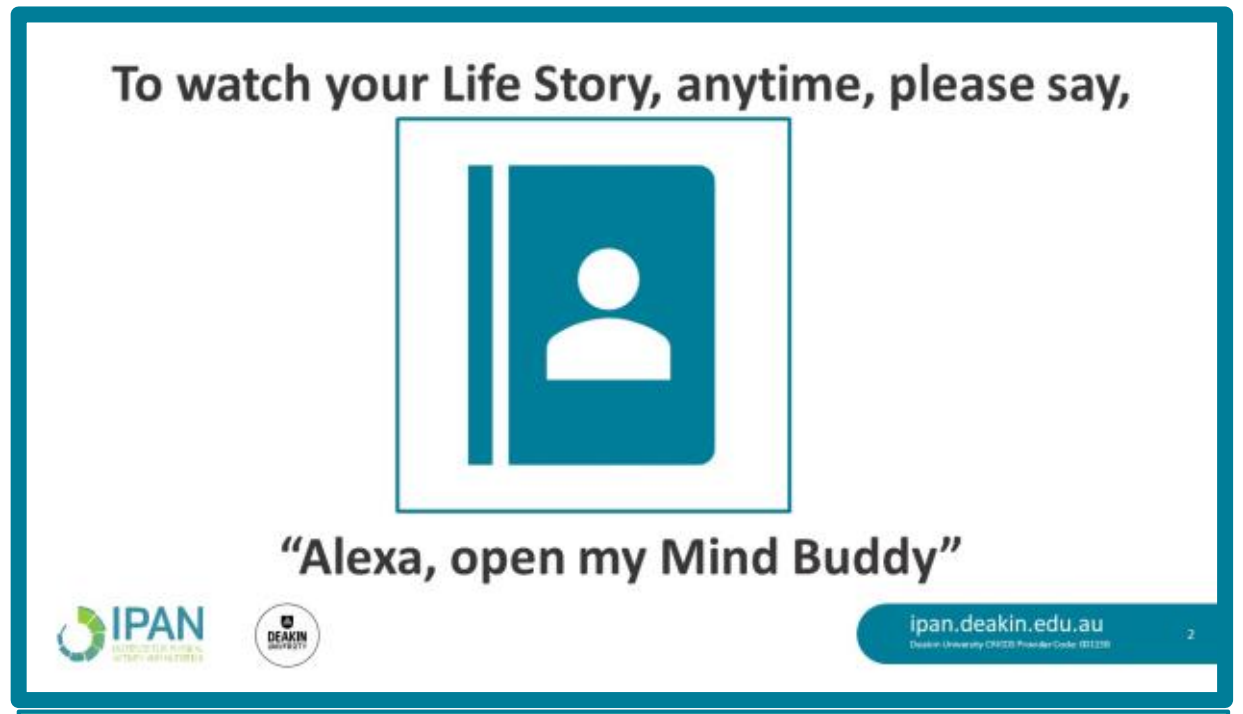

Prompts to watch their life story

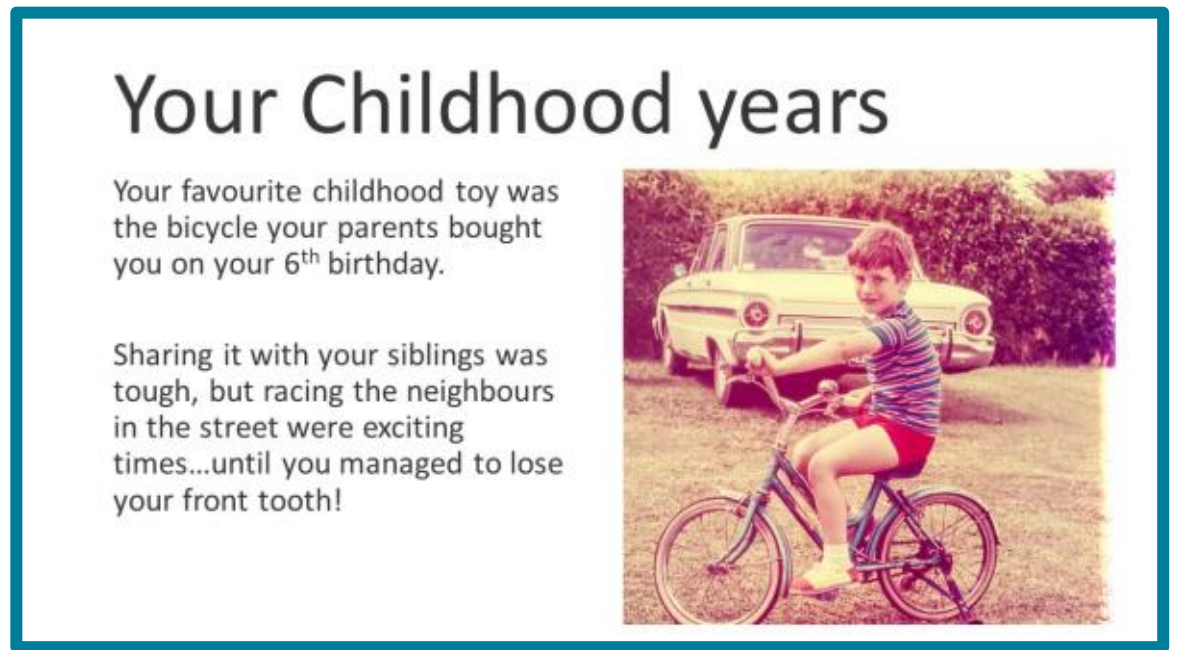

Participants view their Life Story movie

Household Manager

Example Goal: I will learn to use the Alexa to help me with household chores to remember to do a/b/c each day/week. I will use the Alexa to remind me how to use a and b.

Which of the following household activities would you like help with? Please select up to 5 household activities:

- ☐ Making the bed
- ☐ Using the TV Remote
- ☐ Locking the door(s)
- ☐ Cleaning the Bathroom
- ☐ Emptying the dishwasher
- ☐ Taking out the rubbish
- ☐ Mopping
- ☐ Vaccuming
- ☐ Taking out the recycling
- ☐ Feeding the pet
- ☐ Doing the laundry
- ☐ Other

Participants can select to work on Goal(s) associated with Everyday Activities such as Housework

Please complete the form with your details

	Would you like a daily or weekly reminder?	What time would you like to be reminded?	AM or PM	Note 1	Note 2	Note 3	Note 4
				1	1	1	1
Using the TV remote							

Would you like to upload any photo (s) or videos related to using the TV remote?

Drop files or click here to upload

Barriers and facilitators to achieving this goal are identified with a psychologist so personalised prompts can be added to the dialog charts.

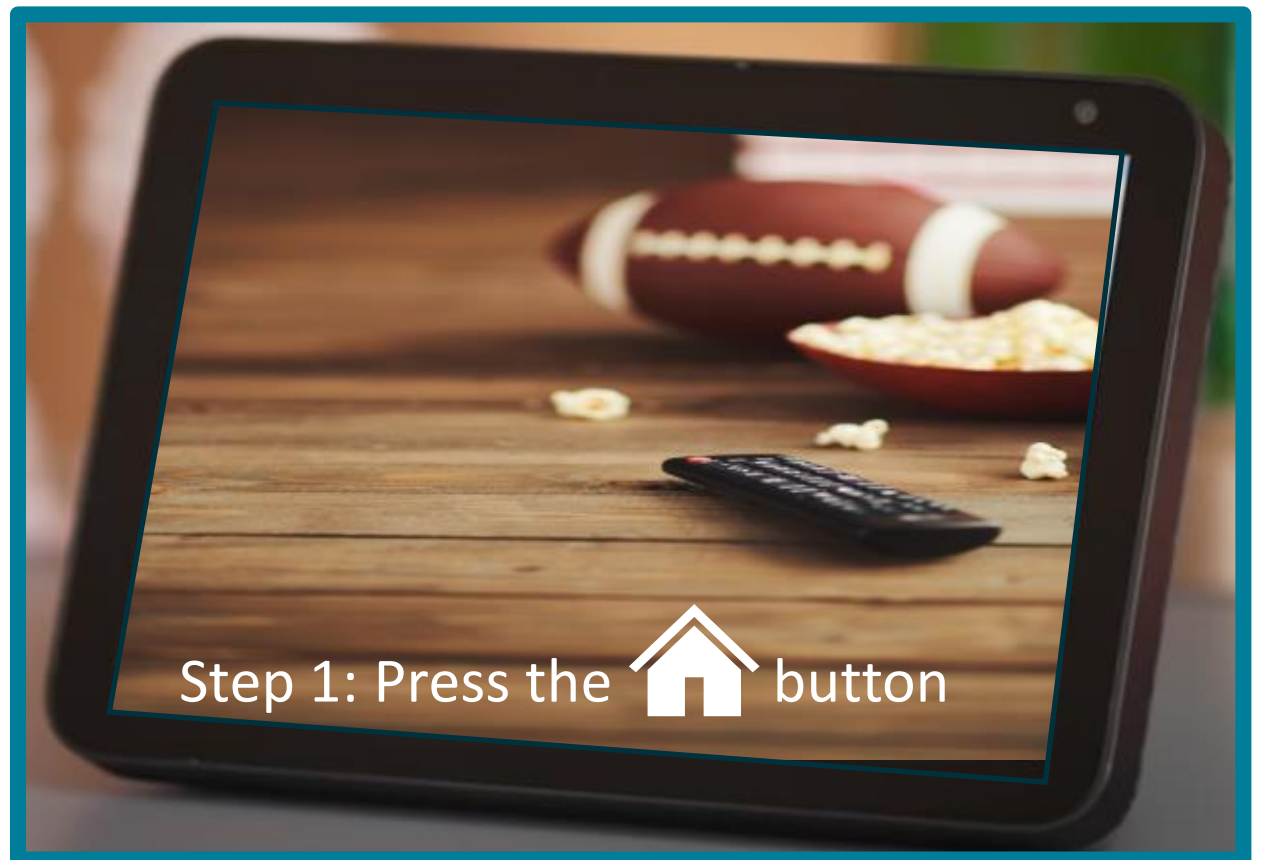

Option for step-by-step instructions using audio, images (personal or stock), and text

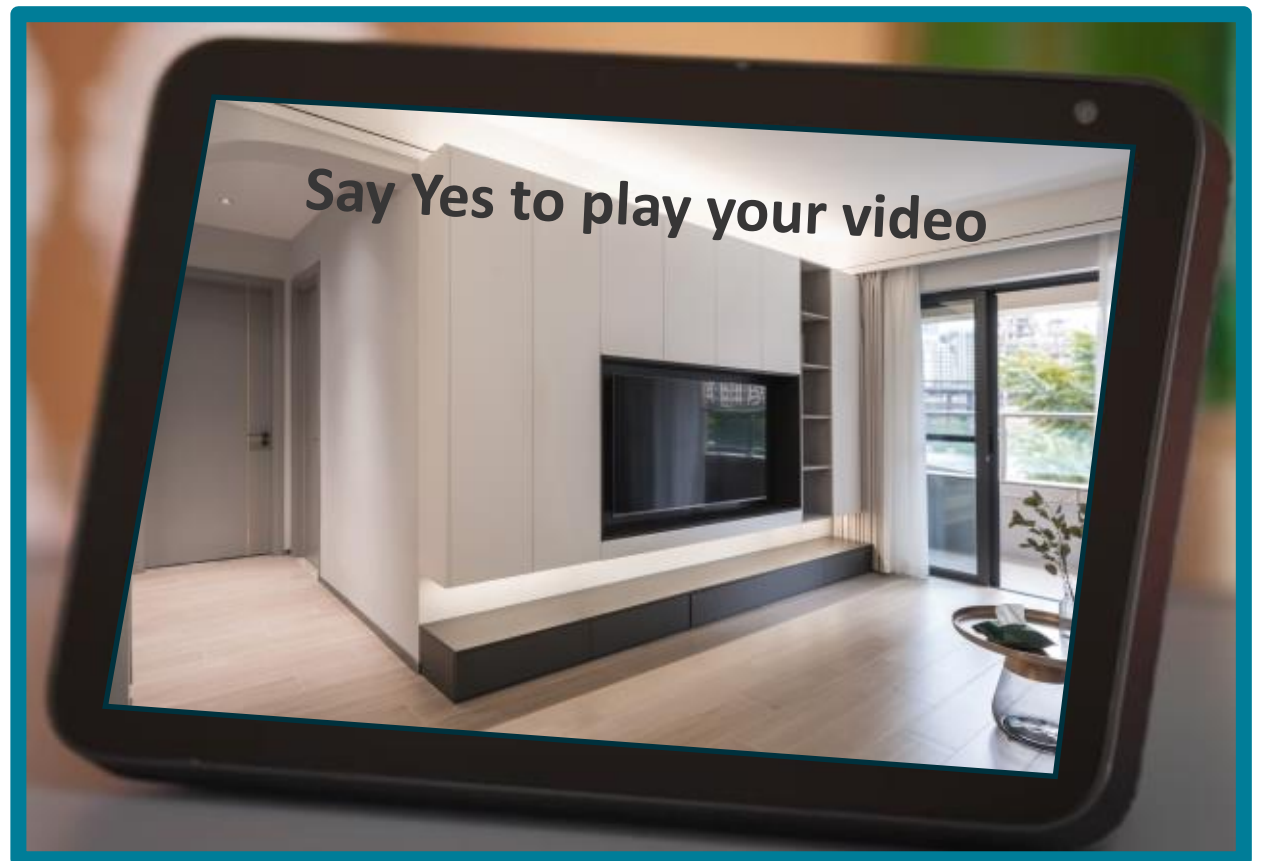

Care partners can upload personal videos that participants can request to watch (or be prompted to watch)

Kitchen Helper

Example Goal: I will use the Kitchen Helper once a week to bake a favourite recipe and use the Alexa to make my shopping list to remember all the ingredients.

WELCOME TO YOUR KITCHEN HELPER

What can I help you with? Please say,

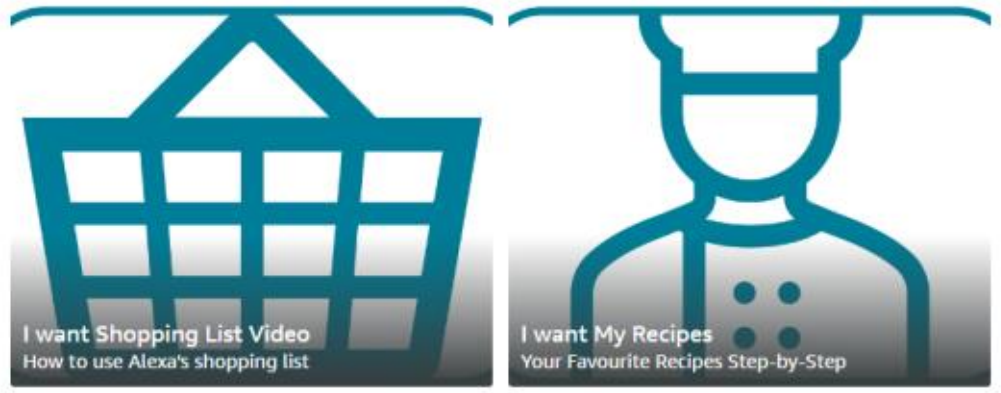

ipan.deakin.edu.au

Pparticipants can initiate or be prompted to open their Kitchen Helper Program

Please write the recipe titles you would like below. You can save the form and come back to it at a later date.

Recipe 1: Zucchini Fritters
Recipe 2: All-in-one Chocolate Cake

What day and time would you like your kitchen helper reminders?

	Day of Week	Hour	AM or PM
Reminder to complete Recipe Form	Monday	10	AM
Reminder to make your recipe	Friday	10	AM

Participants upload personal (reminiscent) recipes

WHICH RECIPE WOULD YOU LIKE TO COOK TODAY?

1. I want Recipe 1
2. I want Recipe 2
3. I want Recipe 3
4. I want Recipe 4
5. I want Recipe 5

Participants select their recipe

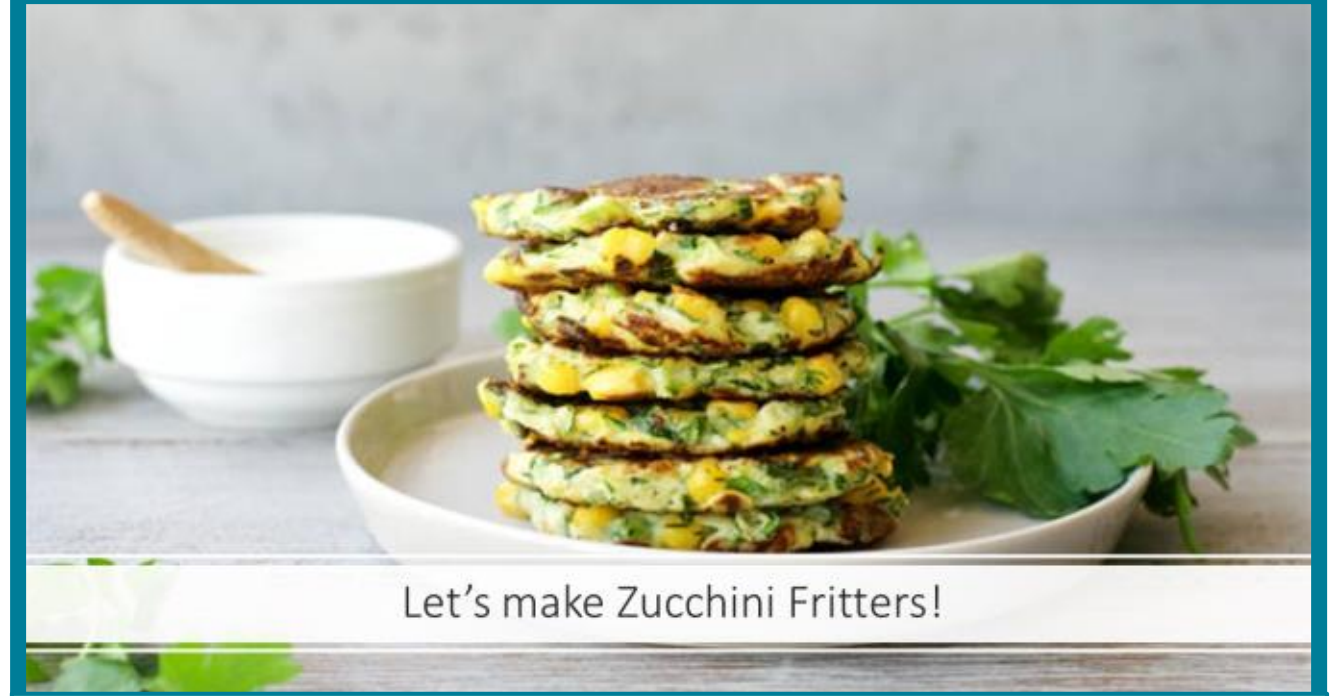

Recipes broadcast using audio, images (personal or stock), and text

INGREDIENTS

- 3 zucchini
- ½ cup self-raising flour
- 2 spring onions
- ½ cup mint
- 1 cup grated cheese
- 1 or 2 eggs depending on size of zucchini
- Olive oil spray

OR

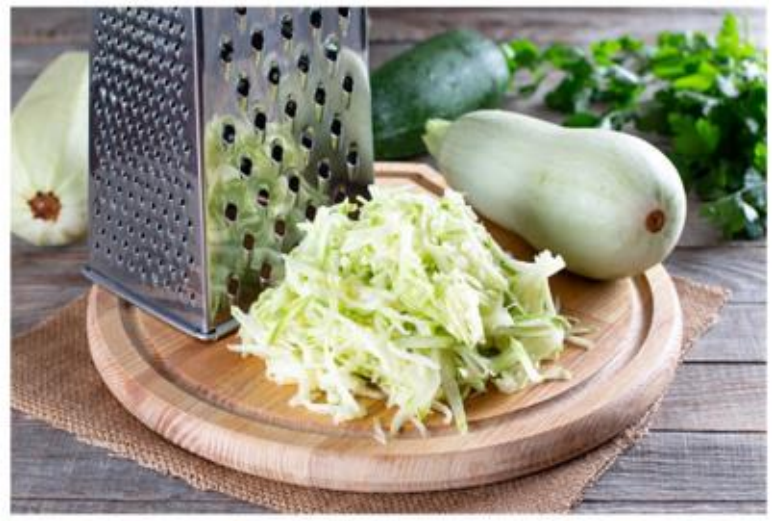

Step 1: Grate the zucchini

Participants follow their recipe, step-by-step, using voice control
