# Supplementary material for: Feasibility of a Co-Designed Personalized Cognitive Reablement Program Delivered via Digital Voice Assistants for Older Adults Living With Early-Stage Dementia or Mild Cognitive Impairment: Pilot Randomized Controlled Trial
Source: JMIR Aging. 2026 Oct 23;9:e96922. doi: 10.2196/96922 (PMC13693962; doi:10.2196/96922)

Appendix 5: Weekly VOICE COG Program Session Broadcasts and mean weekly voice-recorded interactions.

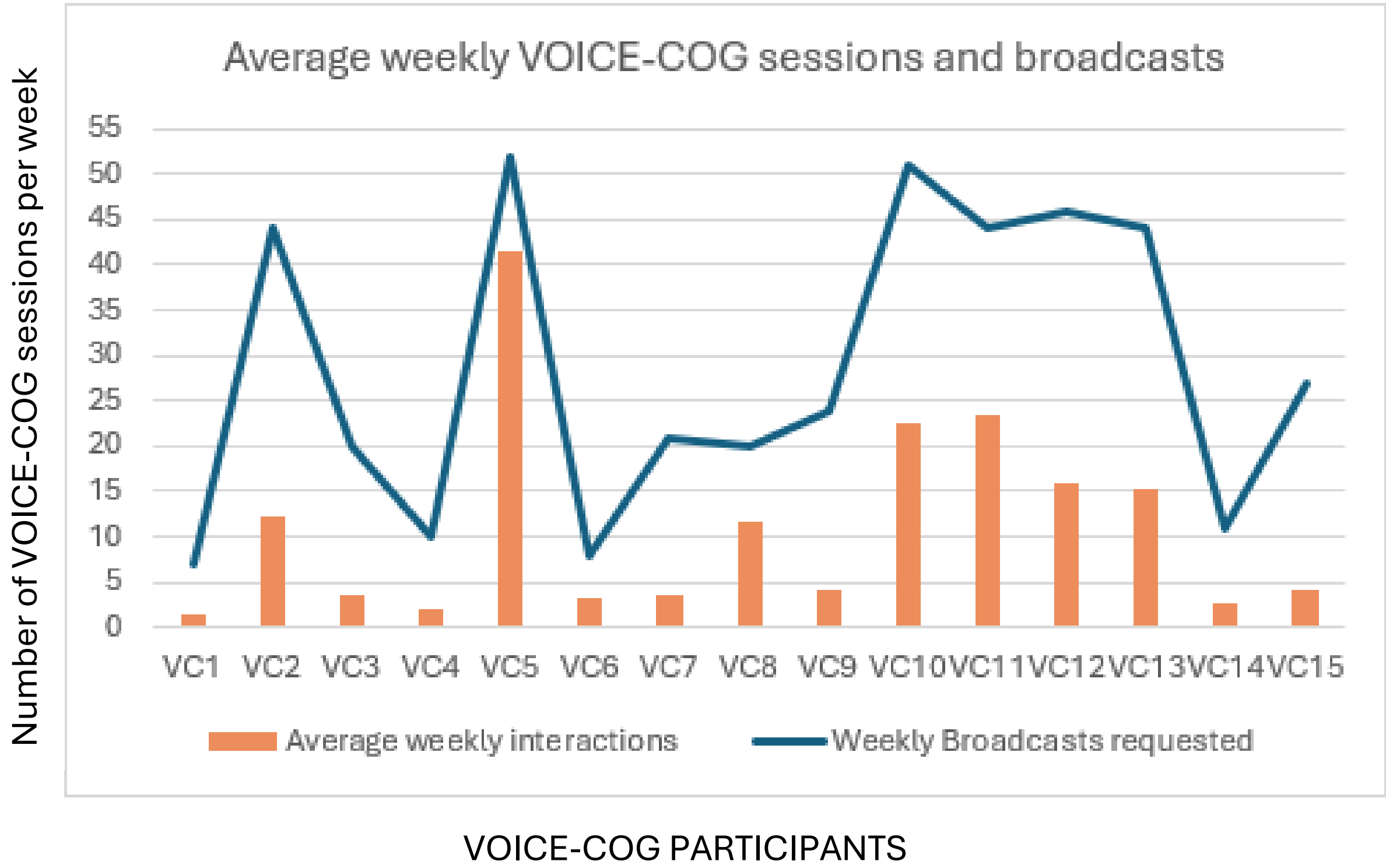

Supplement: Supplementary file 4 [file aging-v9-e96922-s004.pdf]
