# Supplementary material for: Feasibility of a Co-Designed Personalized Cognitive Reablement Program Delivered via Digital Voice Assistants for Older Adults Living With Early-Stage Dementia or Mild Cognitive Impairment: Pilot Randomized Controlled Trial
Source: JMIR Aging. 2026 Oct 23;9:e96922. doi: 10.2196/96922 (PMC13693962; doi:10.2196/96922)
Supplement: Supplementary file 5 [file aging-v9-e96922-s005.pdf]

Appendix 6: System Usability Scale Scores

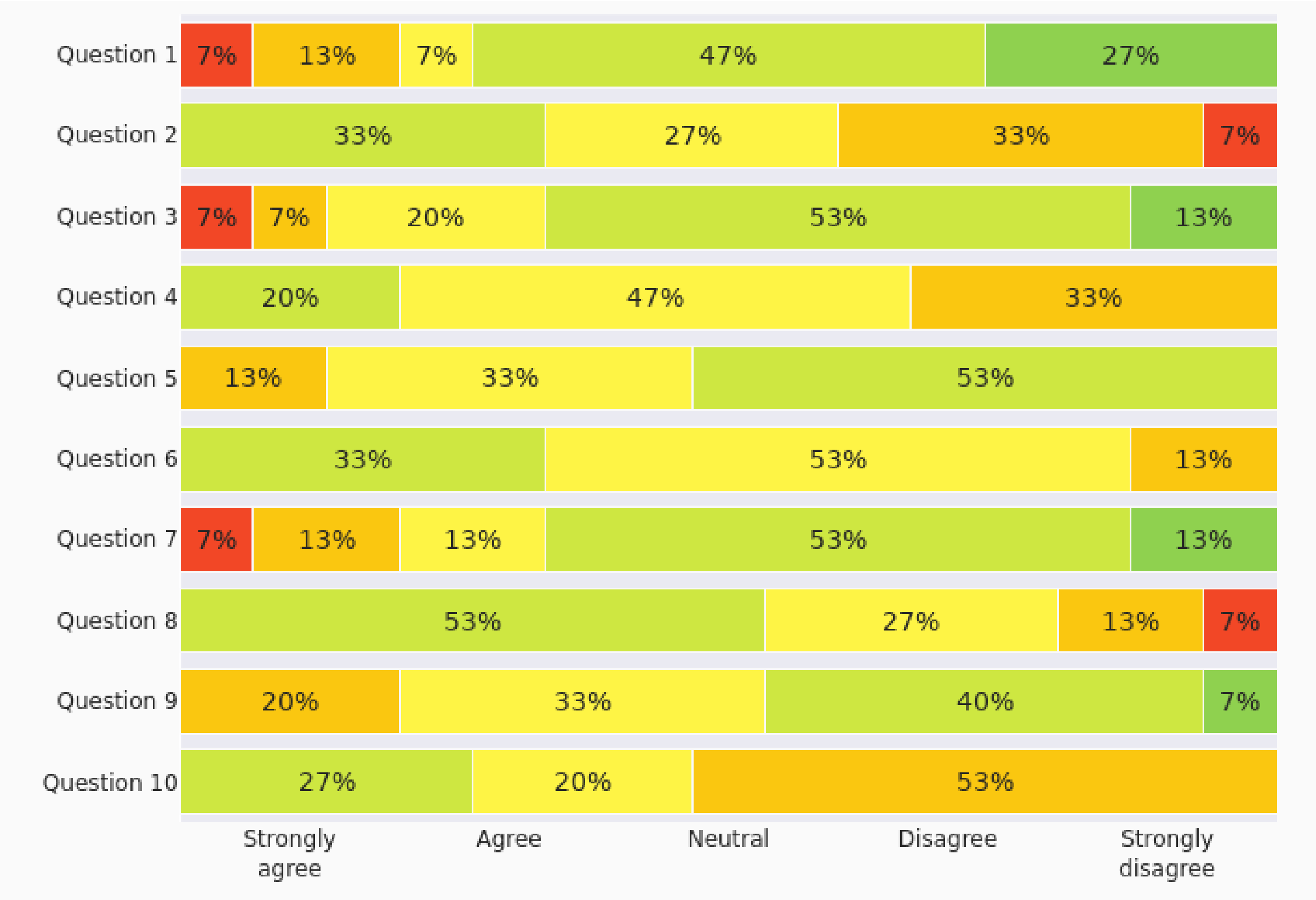

Source: A Web-Based Analysis Toolkit for the System Usability Scale. Blattgerste J, Behrends J, Pfeiffer T. Association for Computing Machinery. 2022 doi: 10.1145/3529190.3529216

Note: Odd-numbered questions are positively worded; Even-numbered questions are negatively worded.

- Strongly agree with positive statements
- Agreement with positive statements or disagreement with negative statements
- Neither agree nor disagree
- Disagreement with positive questions or agreement with negative questions
- Strong disagreement with positive statements or strong agreement with negative statements

Question 1	I think that I would like to use this system frequently
Question 2	I found the system to be unnecessarily complex
Question 3	I thought the system was easy to use
Question 4	I think that I would need to support of a technical person to be able to use the system
Question 5	I found the various functions in the system were well integrated
Question 6	I thought there was too much inconsistency in the system
Question 7	I would imagine that most people would learn to use this system very quickly
Question 8	I found the system very cumbersome to use
Question 9	I felt very confident using the system
Question 10	I needed to learn a lot of things before I could get going with this system
