# Supplementary material for: Feasibility of a Co-Designed Personalized Cognitive Reablement Program Delivered via Digital Voice Assistants for Older Adults Living With Early-Stage Dementia or Mild Cognitive Impairment: Pilot Randomized Controlled Trial
Source: JMIR Aging. 2026 Oct 23;9:e96922. doi: 10.2196/96922 (PMC13693962; doi:10.2196/96922)
Supplement: Supplementary file 6 [file aging-v9-e96922-s006.pdf]

CONSORT-EHEALTH (V 1.6.1) - Submission/Publication Form

The CONSORT-EHEALTH checklist is intended for authors of randomized trials evaluating web-based and Internet-based applications/interventions, including mobile interventions, electronic games (incl multiplayer games), social media, certain telehealth applications, and other interactive and/or networked electronic applications. Some of the items (e.g. all subitems under item 5 - description of the intervention) may also be applicable for other study designs.

The goal of the CONSORT EHEALTH checklist and guideline is to be
a) a guide for reporting for authors of RCTs,
b) to form a basis for appraisal of an ehealth trial (in terms of validity)

CONSORT-EHEALTH items/subitems are MANDATORY reporting items for studies published in the Journal of Medical Internet Research and other journals / scientific societies endorsing the checklist.

Items numbered 1., 2., 3., 4a., 4b etc are original CONSORT or CONSORT-NPT (non-pharmacologic treatment) items.
Items with Roman numerals (i., ii, iii, iv etc.) are CONSORT-EHEALTH extensions/clarifications.

As the CONSORT-EHEALTH checklist is still considered in a formative stage, we would ask that you also RATE ON A SCALE OF 1-5 how important/useful you feel each item is FOR THE PURPOSE OF THE CHECKLIST and reporting guideline (optional).

Mandatory reporting items are marked with a red *.
In the textboxes, either copy & paste the relevant sections from your manuscript into this form - please include any quotes from your manuscript in QUOTATION MARKS,
or answer directly by providing additional information not in the manuscript, or elaborating on why the item was not relevant for this study.

YOUR ANSWERS WILL BE PUBLISHED AS A SUPPLEMENTARY FILE TO YOUR PUBLICATION IN JMIR AND ARE CONSIDERED PART OF YOUR PUBLICATION (IF ACCEPTED).
Please fill in these questions diligently. Information will not be copyedited, so please use proper spelling and grammar, use correct capitalization, and avoid abbreviations.

DO NOT FORGET TO SAVE AS PDF _AND_ CLICK THE SUBMIT BUTTON SO YOUR ANSWERS ARE IN OUR DATABASE !!!

Citation Suggestion (if you append the pdf as Appendix we suggest to cite this paper in the caption):
Eysenbach G, CONSORT-EHEALTH Group
CONSORT-EHEALTH: Improving and Standardizing Evaluation Reports of Web-based and Mobile Health Interventions
J Med Internet Res 2011;13(4):e126

URL: http://www.jmir.org/2011/4/e126/
doi: 10.2196/jmir.1923
PMID: 22209829

Sign in to Google to save your progress. Learn more

* Indicates required question

Your name *

First Last

Tracy Yiannis

Primary Affiliation (short), City, Country *

University of Toronto, Toronto, Canada

Deakin University, Burwood, Australia

Your e-mail address *

abc@gmail.com

tyiannis@deakin.edu.au

Title of your manuscript *

Provide the (draft) title of your manuscript.

Feasibility of a Co-designed Personalized Cognitive Reablement Program Delivered via Digital Voice Assistants for Older Adults Living with Early-Stage Dementia or Mild Cognitive Impairment: A Pilot Randomized Controlled Trial

Name of your App/Software/Intervention *

If there is a short and a long/alternate name, write the short name first and add the long name in brackets.

Buddylink/Amazon Alexa/Voice-Cog

Evaluated Version (if any)

e.g. "V1", "Release 2017-03-01", "Version 2.0.27913"

Your answer

Language(s) *

What language is the intervention/app in? If multiple languages are available, separate by comma (e.g. "English, French")

English

URL of your Intervention Website or App

e.g. a direct link to the mobile app on app in appstore (itunes, Google Play), or URL of the website. If the intervention is a DVD or hardware, you can also link to an Amazon page.

Your answer

URL of an image/screenshot (optional)

Your answer

Accessibility *

Can an enduser access the intervention presently?

- ○ access is free and open
- ○ access only for special usergroups, not open
- ○ access is open to everyone, but requires payment/subscription/in-app purchases
- ◉ app/intervention no longer accessible
- ○ Other:

Primary Medical Indication/Disease/Condition *

e.g. "Stress", "Diabetes", or define the target group in brackets after the condition, e.g. "Autism (Parents of children with)", "Alzheimers (Informal Caregivers of)"

Early-Stage Dementia or Mild Cognitive Impairı

Primary Outcomes measured in trial *

comma-separated list of primary outcomes reported in the trial

Recruitment, Retention, Adherence

Secondary/other outcomes

Are there any other outcomes the intervention is expected to affect?

Goal Attainment, System Usability, Cognitive, Emotional, Functional and Carer Burden

Recommended "Dose" *

What do the instructions for users say on how often the app should be used?

- ( ) Approximately Daily
- ( ) Approximately Weekly
- ( ) Approximately Monthly
- ( ) Approximately Yearly
- ( ) "as needed"
- (•) Other: As per participant (minimum weekly)

Approx. Percentage of Users (starters) still using the app as recommended after 3 months *

- (•) unknown / not evaluated
- ( ) 0-10%
- ( ) 11-20%
- ( ) 21-30%
- ( ) 31-40%
- ( ) 41-50%
- ( ) 51-60%
- ( ) 61-70%
- ( ) 71%-80%
- ( ) 81-90%
- ( ) 91-100%
- ( ) Other:

Overall, was the app/intervention effective? *

- ( ) yes: all primary outcomes were significantly better in intervention group vs control
- ( ) partly: SOME primary outcomes were significantly better in intervention group vs control
- ( ) no statistically significant difference between control and intervention
- ( ) potentially harmful: control was significantly better than intervention in one or more outcomes
- ( ) inconclusive: more research is needed
- (•) Other: Feasibility trial not powered for effects

Article Preparation Status/Stage *

At which stage in your article preparation are you currently (at the time you fill in this form)

- ( ) not submitted yet - in early draft status
- (•) not submitted yet - in late draft status, just before submission
- ( ) submitted to a journal but not reviewed yet
- ( ) submitted to a journal and after receiving initial reviewer comments
- ( ) submitted to a journal and accepted, but not published yet
- ( ) published
- ( ) Other:

Journal *

If you already know where you will submit this paper (or if it is already submitted), please provide the journal name (if it is not JMIR, provide the journal name under "other")

- ○ not submitted yet / unclear where I will submit this
- ○ Journal of Medical Internet Research (JMIR)
- ○ JMIR mHealth and UHealth
- ○ JMIR Serious Games
- ◉ JMIR Mental Health
- ○ JMIR Public Health
- ○ JMIR Formative Research
- ○ Other JMIR sister journal
- ○ Other:

Is this a full powered effectiveness trial or a pilot/feasibility trial? *

- ◉ Pilot/feasibility
- ○ Fully powered

Manuscript tracking number *

If this is a JMIR submission, please provide the manuscript tracking number under "other" (The ms tracking number can be found in the submission acknowledgement email, or when you login as author in JMIR. If the paper is already published in JMIR, then the ms tracking number is the four-digit number at the end of the DOI, to be found at the bottom of each published article in JMIR)

- ◉ no ms number (yet) / not (yet) submitted to / published in JMIR
- ○ Other:

TITLE AND ABSTRACT

1a) TITLE: Identification as a randomized trial in the title

1a) Does your paper address CONSORT item 1a? *

I.e does the title contain the phrase "Randomized Controlled Trial"? (if not, explain the reason under "other")

- (●) yes
- ( ) Other:

1a-i) Identify the mode of delivery in the title

Identify the mode of delivery. Preferably use “web-based” and/or “mobile” and/or “electronic game” in the title. Avoid ambiguous terms like “online”, “virtual”, “interactive”. Use “Internet-based” only if Intervention includes non-web-based Internet components (e.g. email), use “computer-based” or “electronic” only if offline products are used. Use “virtual” only in the context of “virtual reality” (3-D worlds). Use “online” only in the context of “online support groups”. Complement or substitute product names with broader terms for the class of products (such as “mobile” or “smart phone” instead of “iphone”), especially if the application runs on different platforms.

	1	2	3	4	5	
subitem not at all important	( )	( )	( )	( )	(●)	essential

Clear selection

Does your paper address subitem 1a-i? *

Copy and paste relevant sections from manuscript title (include quotes in quotation marks "like this" to indicate direct quotes from your manuscript), or elaborate on this item by providing additional information not in the ms, or briefly explain why the item is not applicable/relevant for your study

Delivered via "Digital Voice Assistants"

1a-ii) Non-web-based components or important co-interventions in title

Mention non-web-based components or important co-interventions in title, if any (e.g., “with telephone support”).

	1	2	3	4	5	
subitem not at all important	○	○	○	◉	○	essential

Clear selection

Does your paper address subitem 1a-ii?

Copy and paste relevant sections from manuscript title (include quotes in quotation marks "like this" to indicate direct quotes from your manuscript), or elaborate on this item by providing additional information not in the ms, or briefly explain why the item is not applicable/relevant for your study

Not included due to space and readability constraints but described in manuscript Methods

1a-iii) Primary condition or target group in the title

Mention primary condition or target group in the title, if any (e.g., “for children with Type I Diabetes”) Example: A Web-based and Mobile Intervention with Telephone Support for Children with Type I Diabetes: Randomized Controlled Trial

	1	2	3	4	5	
subitem not at all important	○	○	○	○	◉	essential

Clear selection

Does your paper address subitem 1a-iii? *

Copy and paste relevant sections from manuscript title (include quotes in quotation marks "like this" to indicate direct quotes from your manuscript), or elaborate on this item by providing additional information not in the ms, or briefly explain why the item is not applicable/relevant for your study

"Early-Stage Dementia or Mild Cognitive Impairment"

1b) ABSTRACT: Structured summary of trial design, methods, results, and conclusions

NPT extension: Description of experimental treatment, comparator, care providers, centers, and blinding status.

1b-i) Key features/functionalities/components of the intervention and comparator in the METHODS section of the ABSTRACT

Mention key features/functionalities/components of the intervention and comparator in the abstract. If possible, also mention theories and principles used for designing the site. Keep in mind the needs of systematic reviewers and indexers by including important synonyms. (Note: Only report in the abstract what the main paper is reporting. If this information is missing from the main body of text, consider adding it)

	1	2	3	4	5	
subitem not at all important	○	○	○	○	◉	essential

Clear selection

Does your paper address subitem 1b-i? *

Copy and paste relevant sections from the manuscript abstract (include quotes in quotation marks "like this" to indicate direct quotes from your manuscript), or elaborate on this item by providing additional information not in the ms, or briefly explain why the item is not applicable/relevant for your study

"Each participant randomized to the intervention group, and their care partner, met with a psychologist via Zoom to select two or three personalized goals from the VOICE-COG program which included i) Activities and Appointments, ii) Routine Reminders, iii) Family and Friends, iv) Mood Monitor, v) Active Minds and vi) Household Manager. The personalized program was input into software (“Buddy Link”: Great Australian Pty Ltd) by researchers for broadcast via a DVA (Amazon Alexa Show 8). Participants in the control group received enhanced usual care, consisting of treatment as usual plus weekly informational emails."

1b-ii) Level of human involvement in the METHODS section of the ABSTRACT

Clarify the level of human involvement in the abstract, e.g., use phrases like “fully automated” vs. “therapist/nurse/care provider/physician-assisted” (mention number and expertise of providers involved, if any). (Note: Only report in the abstract what the main paper is reporting. If this information is missing from the main body of text, consider adding it)

	1	2	3	4	5	
subitem not at all important	○	○	○	◉	○	essential

Clear selection

Does your paper address subitem 1b-ii?

Copy and paste relevant sections from the manuscript abstract (include quotes in quotation marks "like this" to indicate direct quotes from your manuscript), or elaborate on this item by providing additional information not in the ms, or briefly explain why the item is not applicable/relevant for your study

i) "Each participant randomized to the intervention group, and their care partner, met with a psychologist via Zoom to select two or three personalized goals from the VOICE-COG program"; ii) "The personalized program was input into software (“Buddy Link”: Great Australian Pty Ltd) by researchers"

1b-iii) Open vs. closed, web-based (self-assessment) vs. face-to-face assessments in the METHODS section of the ABSTRACT

Mention how participants were recruited (online vs. offline), e.g., from an open access website or from a clinic or a closed online user group (closed usergroup trial), and clarify if this was a purely web-based trial, or there were face-to-face components (as part of the intervention or for assessment). Clearly say if outcomes were self-assessed through questionnaires (as common in web-based trials). Note: In traditional offline trials, an open trial (open-label trial) is a type of clinical trial in which both the researchers and participants know which treatment is being administered. To avoid confusion, use “blinded” or “unblinded” to indicated the level of blinding instead of “open”, as “open” in web-based trials usually refers to “open access” (i.e. participants can self-enrol). (Note: Only report in the abstract what the main paper is reporting. If this information is missing from the main body of text, consider adding it)

	1	2	3	4	5	
subitem not at all important	○	○	○	◉	○	essential

Clear selection

Does your paper address subitem 1b-iii?

Copy and paste relevant sections from the manuscript abstract (include quotes in quotation marks "like this" to indicate direct quotes from your manuscript), or elaborate on this item by providing additional information not in the ms, or briefly explain why the item is not applicable/relevant for your study

Adults aged 60-85 years living with dementia or MCI (N=30) were "recruited via Facebook or the Step-Up-for Dementia Research database"

1b-iv) RESULTS section in abstract must contain use data

Report number of participants enrolled/assessed in each group, the use/uptake of the intervention (e.g., attrition/adherence metrics, use over time, number of logins etc.), in addition to primary/secondary outcomes. (Note: Only report in the abstract what the main paper is reporting. If this information is missing from the main body of text, consider adding it)

	1	2	3	4	5	
subitem not at all important	○	○	○	○	◉	essential

Clear selection

Does your paper address subitem 1b-iv?

Copy and paste relevant sections from the manuscript abstract (include quotes in quotation marks "like this" to indicate direct quotes from your manuscript), or elaborate on this item by providing additional information not in the ms, or briefly explain why the item is not applicable/relevant for your study

i) Methods: "Adults aged 60-85 years living with dementia or MCI (N=30) were recruited via Facebook or the Step-Up-for Dementia Research database and randomized to either a DVA intervention (n=15) or control (n=15) group for 12-weeks."; ii) Results: "Recruitment rate was 86% (30/35) and retention rate 97% (29/30) with one participant in the intervention group lost to follow-up. Adherence to the VOICE-COG program ranged from 29% to 100% (mean 70%, SD 17%)."

1b-v) CONCLUSIONS/DISCUSSION in abstract for negative trials

Conclusions/Discussions in abstract for negative trials: Discuss the primary outcome - if the trial is negative (primary outcome not changed), and the intervention was not used, discuss whether negative results are attributable to lack of uptake and discuss reasons. (Note: Only report in the abstract what the main paper is reporting. If this information is missing from the main body of text, consider adding it)

	1	2	3	4	5	
subitem not at all important	○	○	○	○	◉	essential

Clear selection

Does your paper address subitem 1b-v?

Copy and paste relevant sections from the manuscript abstract (include quotes in quotation marks "like this" to indicate direct quotes from your manuscript), or elaborate on this item by providing additional information not in the ms, or briefly explain why the item is not applicable/relevant for your study

Conclusion: "System usability issues should be addressed for future adequately powered trials"

INTRODUCTION

2a) In INTRODUCTION: Scientific background and explanation of rationale

2a-i) Problem and the type of system/solution

Describe the problem and the type of system/solution that is object of the study: intended as stand-alone intervention vs. incorporated in broader health care program? Intended for a particular patient population? Goals of the intervention, e.g., being more cost-effective to other interventions, replace or complement other solutions? (Note: Details about the intervention are provided in “Methods” under 5)

	1	2	3	4	5	
subitem not at all important	○	○	○	○	◉	essential

Clear selection

Does your paper address subitem 2a-i? *

Copy and paste relevant sections from the manuscript (include quotes in quotation marks "like this" to indicate direct quotes from your manuscript), or elaborate on this item by providing additional information not in the ms, or briefly explain why the item is not applicable/relevant for your study

Introduction: "To date, there has been little agreement on the optimal intervention duration and intensity, with variations in intervals and program durations ranging from 4-weeks to 4-months [10, 12, 14]. Furthermore, health professionals have reported that the substantial time commitment required to deliver these home-based programs, compounded by travel time and the need for specialized training, represent significant barriers to widespread implementation [15, 16]."

2a-ii) Scientific background, rationale: What is known about the (type of) system

Scientific background, rationale: What is known about the (type of) system that is the object of the study (be sure to discuss the use of similar systems for other conditions/diagnoses, if appropiate), motivation for the study, i.e. what are the reasons for and what is the context for this specific study, from which stakeholder viewpoint is the study performed, potential impact of findings [2]. Briefly justify the choice of the comparator.

	1	2	3	4	5	
subitem not at all important	○	○	○	○	◉	essential

Clear selection

Does your paper address subitem 2a-ii? *

Copy and paste relevant sections from the manuscript (include quotes in quotation marks "like this" to indicate direct quotes from your manuscript), or elaborate on this item by providing additional information not in the ms, or briefly explain why the item is not applicable/relevant for your study

Introduction: "To enable delivery of structured health programs in homebased settings for older adults, we have developed "Buddy Link" proprietary software that allows personalized health programs to be broadcast via DVAs tailored to individual user needs [23]. Pilot studies have demonstrated the feasibility of DVA delivered 12-week exercise and nutrition intervention programs for cognitively healthy older adults [24-26]. Existing evidence on DVA use for people living with dementia is confined to exploratory qualitative designs [27] or technical evaluation [28] with small sample sizes, limiting generalization."

2b) In INTRODUCTION: Specific objectives or hypotheses

Does your paper address CONSORT subitem 2b? *

Copy and paste relevant sections from the manuscript (include quotes in quotation marks "like this" to indicate direct quotes from your manuscript), or elaborate on this item by providing additional information not in the ms, or briefly explain why the item is not applicable/relevant for your study

Introduction: "The primary aim of this pilot RCT was to determine if VOICE-COG was feasible for older adults living with dementia to inform a full-scale trial. Secondary aims were to assess system usability and potential efficacy on goal attainment, cognition, emotional wellbeing, daily activity functioning and care partner burden."

METHODS

3a) Description of trial design (such as parallel, factorial) including allocation ratio

Does your paper address CONSORT subitem 3a? *

Copy and paste relevant sections from the manuscript (include quotes in quotation marks "like this" to indicate direct quotes from your manuscript), or elaborate on this item by providing additional information not in the ms, or briefly explain why the item is not applicable/relevant for your study

Methods: Study Design: "Following completion of baseline assessments, participants were block randomized (1:1), stratified by gender, to either the intervention (VOICE-COG program) or control group."

3b) Important changes to methods after trial commencement (such as eligibility criteria), with reasons

Does your paper address CONSORT subitem 3b? *

Copy and paste relevant sections from the manuscript (include quotes in quotation marks "like this" to indicate direct quotes from your manuscript), or elaborate on this item by providing additional information not in the ms, or briefly explain why the item is not applicable/relevant for your study

There were no changes to eligibility criteria.

3b-i) Bug fixes, Downtimes, Content Changes

Bug fixes, Downtimes, Content Changes: ehealth systems are often dynamic systems. A description of changes to methods therefore also includes important changes made on the intervention or comparator during the trial (e.g., major bug fixes or changes in the functionality or content) (5-iii) and other "unexpected events" that may have influenced study design such as staff changes, system failures/downtimes, etc. [2].

	1	2	3	4	5	
subitem not at all important	○	○	○	○	○	essential

Does your paper address subitem 3b-i?

Copy and paste relevant sections from the manuscript (include quotes in quotation marks "like this" to indicate direct quotes from your manuscript), or elaborate on this item by providing additional information not in the ms, or briefly explain why the item is not applicable/relevant for your study

Results: The Kitchen Helper Program goal had to be withdrawn after 2 weeks due to technical issues with device response timing and voice command difficulties navigating recipes

4a) Eligibility criteria for participants

Does your paper address CONSORT subitem 4a? *

Copy and paste relevant sections from the manuscript (include quotes in quotation marks "like this" to indicate direct quotes from your manuscript), or elaborate on this item by providing additional information not in the ms, or briefly explain why the item is not applicable/relevant for your study

Methods: Participants: Eligibility criteria included older adults (aged 60 to 85 years) with a self-reported, recent (≤ 6 months) clinical diagnosis of dementia, or mild cognitive impairment (MCI), living anywhere in Australia with access to a home Wi-Fi network. Participants were required to be fluent in English, have a care partner with at least 50% close contact during the 12-week trial, and have access to a smartphone capable of making and receiving calls on an Australian network. Participants were excluded if they: (i) were deemed to have difficulty communicating with a DVA due to speech or hearing problems, (ii) were unwilling to be randomized, (iii) planned to be away for ≥ 4 weeks during the 12-week intervention period, or (iv) had any self-reported cognitive or physical impairment, that in the opinion of the research team would result in the participant having difficulty interacting with the DVA, or performing the VOICE COG program safely. This included a self-reported clinical diagnosis of schizophrenia, bipolar disorder, or other psychotic disorders.

4a-i) Computer / Internet literacy

Computer / Internet literacy is often an implicit "de facto" eligibility criterion - this should be explicitly clarified.

	1	2	3	4	5	
subitem not at all important	○	○	○	◉	○	essential

Clear selection

Does your paper address subitem 4a-i?

Copy and paste relevant sections from the manuscript (include quotes in quotation marks "like this" to indicate direct quotes from your manuscript), or elaborate on this item by providing additional information not in the ms, or briefly explain why the item is not applicable/relevant for your study

We did not collect data on computer literacy as described under Limitations.

4a-ii) Open vs. closed, web-based vs. face-to-face assessments:

Open vs. closed, web-based vs. face-to-face assessments: Mention how participants were recruited (online vs. offline), e.g., from an open access website or from a clinic, and clarify if this was a purely web-based trial, or there were face-to-face components (as part of the intervention or for assessment), i.e., to what degree got the study team to know the participant. In online-only trials, clarify if participants were quasi-anonymous and whether having multiple identities was possible or whether technical or logistical measures (e.g., cookies, email confirmation, phone calls) were used to detect/prevent these.

	1	2	3	4	5	
subitem not at all important	○	○	○	○	◉	essential

Clear selection

Does your paper address subitem 4a-ii? *

Copy and paste relevant sections from the manuscript (include quotes in quotation marks "like this" to indicate direct quotes from your manuscript), or elaborate on this item by providing additional information not in the ms, or briefly explain why the item is not applicable/relevant for your study

Methods: Participants: "Participants were recruited via two methods. First, through paid targeted Facebook advertisements where potential participants clicked an embedded hyperlink to read the Plain Language Statement and completed an Expression of Interest form via Qualtrics (Provo, UT, USA). Second, eligible volunteers matching the study's inclusion criteria from the Step Up for Dementia research database were contacted via phone call or email [34]. Individuals in this database have previously provided consent to be contacted for research. Interested participants were screened for eligibility via telephone" and then emailed a link to the Plain Language and Consent Form on Qualtrics to provide written or electronic informed consent."

4a-iii) Information giving during recruitment

Information given during recruitment. Specify how participants were briefed for recruitment and in the informed consent procedures (e.g., publish the informed consent documentation as appendix, see also item X26), as this information may have an effect on user self-selection, user expectation and may also bias results.

	1	2	3	4	5	
subitem not at all important	○	○	○	○	◉	essential

Clear selection

Does your paper address subitem 4a-iii?

Copy and paste relevant sections from the manuscript (include quotes in quotation marks "like this" to indicate direct quotes from your manuscript), or elaborate on this item by providing additional information not in the ms, or briefly explain why the item is not applicable/relevant for your study

Methods: Participants Interested participants were screened for eligibility via telephone and then "emailed a link to the Plain Language and Consent Form on Qualtrics to provide written or electronic informed consent."

4b) Settings and locations where the data were collected

Does your paper address CONSORT subitem 4b? *

Copy and paste relevant sections from the manuscript (include quotes in quotation marks "like this" to indicate direct quotes from your manuscript), or elaborate on this item by providing additional information not in the ms, or briefly explain why the item is not applicable/relevant for your study

Methods: "All outcome measures were collated remotely using self-assessed or care partner informed assessments via Qualtrics."

4b-i) Report if outcomes were (self-)assessed through online questionnaires

Clearly report if outcomes were (self-)assessed through online questionnaires (as common in web-based trials) or otherwise.

	1	2	3	4	5	
subitem not at all important	○	○	○	○	◉	essential

Clear selection

Does your paper address subitem 4b-i? *

Copy and paste relevant sections from the manuscript (include quotes in quotation marks "like this" to indicate direct quotes from your manuscript), or elaborate on this item by providing additional information not in the ms, or briefly explain why the item is not applicable/relevant for your study

Methods: i) Secondary outcomes: "All outcome measures were collated remotely using self-assessed or care partner informed assessments via Qualtrics. Demographic data was collated via an online survey at baseline." ii) Goal attainment scale: "The baseline goal attainment score was entered via the online Qualtrics goal setting form by the psychologist (-2: No function or -1: Some function) during the goal setting meeting. After 12 weeks participants were emailed their personal goals, and together with their care partner, reported if they had achieved their goal and to what level"

4b-ii) Report how institutional affiliations are displayed

Report how institutional affiliations are displayed to potential participants [on ehealth media], as affiliations with prestigious hospitals or universities may affect volunteer rates, use, and reactions with regards to an intervention.(Not a required item – describe only if this may bias results)

	1	2	3	4	5	
subitem not at all important	○	○	○	○	◉	essential

Clear selection

Does your paper address subitem 4b-ii?

Copy and paste relevant sections from the manuscript (include quotes in quotation marks "like this" to indicate direct quotes from your manuscript), or elaborate on this item by providing additional information not in the ms, or briefly explain why the item is not applicable/relevant for your study

Recruitment advertisements used the University's Facebook page. The Plain Language statement also referred to the institution.

5) The interventions for each group with sufficient details to allow replication, including how and when they were actually administered

5-i) Mention names, credential, affiliations of the developers, sponsors, and owners

Mention names, credential, affiliations of the developers, sponsors, and owners [6] (if authors/evaluators are owners or developer of the software, this needs to be declared in a “Conflict of interest” section or mentioned elsewhere in the manuscript).

	1	2	3	4	5	
subitem not at all important	○	○	○	○	◉	essential

Clear selection

Does your paper address subitem 5-i?

Copy and paste relevant sections from the manuscript (include quotes in quotation marks "like this" to indicate direct quotes from your manuscript), or elaborate on this item by providing additional information not in the ms, or briefly explain why the item is not applicable/relevant for your study

Conflicts of Interest: "EG is a director (unpaid position) of Great Australian Pty Ltd. Great Australian Pty Ltd provided in-kind technical support for software and had a nonfinancial interest in the development of a voice-based digital assistant software for telehealth. Great Australian had no input into this study’s design, analysis, or interpretation of the results, or the decision to publish"

5-ii) Describe the history/development process

Describe the history/development process of the application and previous formative evaluations (e.g., focus groups, usability testing), as these will have an impact on adoption/use rates and help with interpreting results.

	1	2	3	4	5	
subitem not at all important	○	○	○	◉	○	essential

Clear selection

Does your paper address subitem 5-ii?

Copy and paste relevant sections from the manuscript (include quotes in quotation marks "like this" to indicate direct quotes from your manuscript), or elaborate on this item by providing additional information not in the ms, or briefly explain why the item is not applicable/relevant for your study

Introduction: "we co-designed the VOICE-COG program involving older adults living with dementia, their care partners, and health professionals (Yiannis et al, manuscript under review)." "Guided by the IDEAS (Integrate, Design, Assess and Share) framework, VOICE-COG delivers cognitive compensation, stimulation, and reminiscence strategies to support people living with dementia/MCI in challenges encountered in daily activities."

5-iii) Revisions and updating

Revisions and updating. Clearly mention the date and/or version number of the application/intervention (and comparator, if applicable) evaluated, or describe whether the intervention underwent major changes during the evaluation process, or whether the development and/or content was "frozen" during the trial. Describe dynamic components such as news feeds or changing content which may have an impact on the replicability of the intervention (for unexpected events see item 3b).

	1	2	3	4	5	
subitem not at all important	○	○	○	◉	○	essential

Clear selection

Does your paper address subitem 5-iii?

Copy and paste relevant sections from the manuscript (include quotes in quotation marks "like this" to indicate direct quotes from your manuscript), or elaborate on this item by providing additional information not in the ms, or briefly explain why the item is not applicable/relevant for your study

Not applicable - no major changes

5-iv) Quality assurance methods

Provide information on quality assurance methods to ensure accuracy and quality of information provided [1], if applicable.

	1	2	3	4	5	
subitem not at all important	○	○	○	◉	○	essential

Clear selection

Does your paper address subitem 5-iv?

Copy and paste relevant sections from the manuscript (include quotes in quotation marks "like this" to indicate direct quotes from your manuscript), or elaborate on this item by providing additional information not in the ms, or briefly explain why the item is not applicable/relevant for your study

Individual personalised content underwent quality assurance checks for accuracy by a research assistant and project lead prior to broadcast.

5-v) Ensure replicability by publishing the source code, and/or providing screenshots/screen-capture video, and/or providing flowcharts of the algorithms used

Ensure replicability by publishing the source code, and/or providing screenshots/screen-capture video, and/or providing flowcharts of the algorithms used. Replicability (i.e., other researchers should in principle be able to replicate the study) is a hallmark of scientific reporting.

	1	2	3	4	5	
subitem not at all important	○	○	○	◉	○	essential

Clear selection

Does your paper address subitem 5-v?

Copy and paste relevant sections from the manuscript (include quotes in quotation marks "like this" to indicate direct quotes from your manuscript), or elaborate on this item by providing additional information not in the ms, or briefly explain why the item is not applicable/relevant for your study

Appendix 2 describes the intervention in further detail.

5-vi) Digital preservation

Digital preservation: Provide the URL of the application, but as the intervention is likely to change or disappear over the course of the years; also make sure the intervention is archived (Internet Archive, webcitation.org, and/or publishing the source code or screenshots/videos alongside the article). As pages behind login screens cannot be archived, consider creating demo pages which are accessible without login.

	1	2	3	4	5	
subitem not at all important	○	○	○	◉	○	essential

Clear selection

Does your paper address subitem 5-vi?

Copy and paste relevant sections from the manuscript (include quotes in quotation marks "like this" to indicate direct quotes from your manuscript), or elaborate on this item by providing additional information not in the ms, or briefly explain why the item is not applicable/relevant for your study

The intervention was not publicly available being delivered via a controlled platform with access limited to study participants only. Therefore digital preservation was not applicable.

5-vii) Access

Access: Describe how participants accessed the application, in what setting/context, if they had to pay (or were paid) or not, whether they had to be a member of specific group. If known, describe how participants obtained "access to the platform and Internet" [1]. To ensure access for editors/reviewers/readers, consider to provide a "backdoor" login account or demo mode for reviewers/readers to explore the application (also important for archiving purposes, see vi).

	1	2	3	4	5	
subitem not at all important	○	○	○	○	◉	essential

Clear selection

Does your paper address subitem 5-vii? *

Copy and paste relevant sections from the manuscript (include quotes in quotation marks "like this" to indicate direct quotes from your manuscript), or elaborate on this item by providing additional information not in the ms, or briefly explain why the item is not applicable/relevant for your study

Methods: Intervention: "Participants randomized to the intervention group, were mailed a pre-registered Amazon Alexa Echo Show 8 device ("Alexa") with instructions on how to connect the device to their home Wi-Fi.";
"Each participant's goal-related content was uploaded remotely to the "Buddy Link" software (Great Australian Pty Ltd) [23] by a research team member, who also configured "program broadcast routines" using deidentified individual Alexa accounts. A voice-initiated routine was added, "Alexa, Open Mind Buddy," for participants to access their program outside these times."

5-viii) Mode of delivery, features/functionalities/components of the intervention and comparator, and the theoretical framework

Describe mode of delivery, features/functionalities/components of the intervention and comparator, and the theoretical framework [6] used to design them (instructional strategy [1], behaviour change techniques, persuasive features, etc., see e.g., [7, 8] for terminology). This includes an in-depth description of the content (including where it is coming from and who developed it) [1],” whether [and how] it is tailored to individual circumstances and allows users to track their progress and receive feedback” [6]. This also includes a description of communication delivery channels and – if computer-mediated communication is a component – whether communication was synchronous or asynchronous [6]. It also includes information on presentation strategies [1], including page design principles, average amount of text on pages, presence of hyperlinks to other resources, etc. [1].

	1	2	3	4	5	
subitem not at all important	○	○	○	○	◉	essential

Clear selection

Does your paper address subitem 5-viii? *

Copy and paste relevant sections from the manuscript (include quotes in quotation marks "like this" to indicate direct quotes from your manuscript), or elaborate on this item by providing additional information not in the ms, or briefly explain why the item is not applicable/relevant for your study

Methods: Intervention: "Intervention group participants selected goals from the VOICE-COG program that were personalized and broadcast via a DVA in their homes. Participants randomized to the intervention group, were mailed a pre-registered Amazon Alexa Echo Show 8 device ("Alexa") with instructions on how to connect the device to their home Wi-Fi. Each participant and their care partner met with a psychologist (qualified to a minimum Doctorate in Clinical Psychology with experience in motivational interviewing) via Zoom (Zoom Video Communications Inc, San Jose, CA, USA), to collaboratively set two or three goals from the VOICE-COG program. The VOICE-COG program was co-designed with older adults living with dementia/MCI and health professionals, details of which are reported elsewhere (Yiannis et al, manuscript under review). In summary, seven goals were offered to support reablement. These included i) Activities and Appointments (attending regular weekly community activities and remembering appointments), ii) Routine Reminders (adherence to daily medication, hydration and nutrition), iii) Family and Friends (writing their Life Story and maintaining relationships), iv) Mood Monitor (providing coping activities such as music and mindfulness), v) Active Minds (providing cognitive stimulation through audiobooks, podcasts, news and short videos), vi) Household Manager (remembering when and how to do home chores) and vii) Kitchen Helper (support for cooking favorite recipes). Further descriptions of the VOICE-COG modules can be found in Appendix 2.
Personalized goal-related information and preferred program broadcast timings were entered into an online Qualtrics form by the psychologist during the goal-setting meeting. Each participant's goal-related content was uploaded remotely to the "Buddy Link" software (Great Australian Pty Ltd) [23] by a research team member, who also configured program broadcast routines using deidentified individual Alexa accounts. A voice-initiated routine was added, "Alexa, Open Mind Buddy," for participants to access their program outside these times."

5-ix) Describe use parameters

Describe use parameters (e.g., intended "doses" and optimal timing for use). Clarify what instructions or recommendations were given to the user, e.g., regarding timing, frequency, heaviness of use, if any, or was the intervention used ad libitum.

	1	2	3	4	5	
subitem not at all important	○	○	○	◉	○	essential

Clear selection

Does your paper address subitem 5-ix?

Copy and paste relevant sections from the manuscript (include quotes in quotation marks "like this" to indicate direct quotes from your manuscript), or elaborate on this item by providing additional information not in the ms, or briefly explain why the item is not applicable/relevant for your study

Results: Primary Feasibility Outcomes: "Participants nominated between 7 and 52 VOICE-COG broadcasts per week"

5-x) Clarify the level of human involvement

Clarify the level of human involvement (care providers or health professionals, also technical assistance) in the e-intervention or as co-intervention (detail number and expertise of professionals involved, if any, as well as "type of assistance offered, the timing and frequency of the support, how it is initiated, and the medium by which the assistance is delivered". It may be necessary to distinguish between the level of human involvement required for the trial, and the level of human involvement required for a routine application outside of a RCT setting (discuss under item 21 – generalizability).

	1	2	3	4	5	
subitem not at all important	○	○	○	●	○	essential

Clear selection

Does your paper address subitem 5-x?

Copy and paste relevant sections from the manuscript (include quotes in quotation marks "like this" to indicate direct quotes from your manuscript), or elaborate on this item by providing additional information not in the ms, or briefly explain why the item is not applicable/relevant for your study

Methods: Intervention: i) Each participant and their care partner "met with a psychologist (qualified to a minimum Doctorate in Clinical Psychology with experience in motivational interviewing) via Zoom" (Zoom Video Communications Inc, San Jose, CA, USA), to collaboratively set two or three goals from the VOICE-COG program.
ii) "Each participant's goal-related content was uploaded remotely to the "Buddy Link" software (Great Australian Pty Ltd) [23] by a research team member, who also configured program broadcast routines using deidentified individual Alexa accounts."

5-xi) Report any prompts/reminders used

Report any prompts/reminders used: Clarify if there were prompts (letters, emails, phone calls, SMS) to use the application, what triggered them, frequency etc. It may be necessary to distinguish between the level of prompts/reminders required for the trial, and the level of prompts/reminders for a routine application outside of a RCT setting (discuss under item 21 – generalizability).

	1	2	3	4	5	
subitem not at all important	○	○	○	○	◉	essential

Clear selection

Does your paper address subitem 5-xi? *

Copy and paste relevant sections from the manuscript (include quotes in quotation marks "like this" to indicate direct quotes from your manuscript), or elaborate on this item by providing additional information not in the ms, or briefly explain why the item is not applicable/relevant for your study

Methods: Intervention: Personalized goal-related information and "preferred program broadcast timings" were entered into an online Qualtrics form by the psychologist during the goal-setting meeting.
ii) Participants who elected to write their Life Story (Family & Friends goal) "received weekly emails" with a link to an online Qualtrics form for data collection.

5-xii) Describe any co-interventions (incl. training/support)

Describe any co-interventions (incl. training/support): Clearly state any interventions that are provided in addition to the targeted eHealth intervention, as ehealth intervention may not be designed as stand-alone intervention. This includes training sessions and support [1]. It may be necessary to distinguish between the level of training required for the trial, and the level of training for a routine application outside of a RCT setting (discuss under item 21 – generalizability.

	1	2	3	4	5	
subitem not at all important	○	○	○	○	◉	essential

Clear selection

Does your paper address subitem 5-xii? *

Copy and paste relevant sections from the manuscript (include quotes in quotation marks "like this" to indicate direct quotes from your manuscript), or elaborate on this item by providing additional information not in the ms, or briefly explain why the item is not applicable/relevant for your study

Methods: Intervention: "Following the meeting, the intervention group were emailed a personalized two-page instruction summary for the selected VOICE-COG goals."

6a) Completely defined pre-specified primary and secondary outcome measures, including how and when they were assessed

Does your paper address CONSORT subitem 6a? *

Copy and paste relevant sections from the manuscript (include quotes in quotation marks "like this" to indicate direct quotes from your manuscript), or elaborate on this item by providing additional information not in the ms, or briefly explain why the item is not applicable/relevant for your study

Methods: "Feasibility
Participant recruitment, retention, and adherence rates were used to examine feasibility of the VOICE-COG program to inform a future RCT. Recruitment rate (%) was defined as the total number of participants who provided consent at baseline divided by total number eligible. Retention rate (%) was specified as the number of participants who completed post-intervention 12-week data collection divided by the number of participants randomized. Adherence (%) was the number of weeks at least one positive voice response was recorded to their personalized VOICE-COG program goal via the DVA, as a percentage of possible weekly interactions over 12-weeks. Pre-defined targets of ≥70% retention and ≥66% adherence to the VOICE-COG program were established based on previous evidence for DVA health intervention studies [24-26].
Secondary Outcome Measures
All outcome measures were collated remotely using self-assessed or care partner informed assessments via Qualtrics. Demographic data was collated via an online survey at baseline.
Goal Attainment
DVA participants' goal attainment was measured using GAS-Light, a valid and reliable scoring system recommended for use with people living with dementia/MCI in reablement programs [36-38]. The baseline goal attainment score was entered via the online Qualtrics goal setting form by the psychologist (-2: No function or -1: Some function) during the goal setting meeting. After 12 weeks participants were emailed their personal goals, and together with their care partner, reported if they had achieved their goal and to what level: Yes (+2 A lot more; +1 A little more; 0 As expected) or No (-0.5 Partially achieved; -1 No change; -2 Got worse). Results were entered into the GAS-light spreadsheet [39] to calculate each participant's GAS-light T-score where T≥50 is interpreted as met or exceeded expectations.
System Usability
Perceived usability of the VOICE-COG skill was assessed by DVA participants using the System Usability Scale (SUS) [40] form administered online via Qualtrics. The SUS uses ten Likert-scale questions to determine subjective usability, providing a score from 0 to 100.
Participant Questionnaires
Secondary outcome measures included differences in the following participant questionnaires (measuring mood, memory and cognition) completed via online Qualtrics forms at baseline and 12-weeks. The Geriatric Depression Scale (GDS-short form) consists of 15-items rated "yes" or "no" to screen for depression in older adults and validated for use in older adults with mild to moderate dementia [41]. Scores of >5 suggest depression may be present; >10 is indicative of major depression. The Multifactorial Memory Questionnaire (MMQ) is memory evaluation tool with 57-items covering three sub-scales – memory satisfaction, ability, and strategy use [42]. Participants' subjective memory experiences from the previous 2 weeks are rated using a 5-point Likert scale from 0 to 4. If there were <4 missing responses for any scale, scores were calculated using the pro-rated formula; for ≥4 missing items, participant scores were excluded from analysis [42]. The Illness Cognition Questionnaire (ICQ) is an 18-item questionnaire using a 4-point Likert scale from 1 (disagree) to 4 (strongly agree) to measure the respondents' perception of their own illness with three subscales of acceptance, disease benefit and helplessness [43]. For participants with one missing item in a subscale, the missing item was imputed using the mean of the other completed items within that subscale (provided at least 4 of 6 items were present).
Care Partner Questionnaires
At baseline and 12 weeks, care partners completed the following surveys: Bayer Activities of Daily Living Scale (B-ADL) is a 25-item questionnaire, assessing difficulty the person living with dementia/MCI has in performing daily activities using a Likert scale of 0 (never') to 10 (always') [44]. The B-ADL score sums all items, excluding those rated 'not applicable' or 'unknown' divided by the number of items rated. The Everyday Cognition Scale (E-Cog)

includes 39-items across six domains (everyday memory, language, visuospatial abilities, planning, organization and divided attention) [45]. The Zarit Caregiver Burden 22-item questionnaire asked respondents to rate the level of burden caused with using a Likert scale of 1 (“none”) to 5 (“nearly always”) giving a score range of No Burden (22 to 46), mild burden (47 to 55) or severe burden (56-110) [46]."

6a-i) Online questionnaires: describe if they were validated for online use and apply CHERRIES items to describe how the questionnaires were designed/deployed

If outcomes were obtained through online questionnaires, describe if they were validated for online use and apply CHERRIES items to describe how the questionnaires were designed/deployed [9].

	1	2	3	4	5	
subitem not at all important	○	○	○	◉	○	essential

Clear selection

Does your paper address subitem 6a-i?

Copy and paste relevant sections from manuscript text

Questionnaires not validated for online use were mentioned under Limitations in the Discussion section.

6a-ii) Describe whether and how "use" (including intensity of use/dosage) was defined/measured/monitored

Describe whether and how "use" (including intensity of use/dosage) was defined/measured/monitored (logins, logfile analysis, etc.). Use/adoption metrics are important process outcomes that should be reported in any ehealth trial.

	1	2	3	4	5	
subitem not at all important	○	○	○	◉	○	essential

Clear selection

Does your paper address subitem 6a-ii?

Copy and paste relevant sections from manuscript text

Methods: Primary Outcome Measures: "Adherence (%) was the number of weeks at least one positive voice response was recorded to their personalized VOICE-COG program goal via the DVA, as a percentage of possible weekly interactions over 12-weeks. Pre-defined targets of ≥70% retention and ≥66% adherence to the VOICE-COG program were established" based on previous evidence for DVA health intervention studies

6a-iii) Describe whether, how, and when qualitative feedback from participants was obtained

Describe whether, how, and when qualitative feedback from participants was obtained (e.g., through emails, feedback forms, interviews, focus groups).

	1	2	3	4	5	
subitem not at all important	○	○	○	◉	○	essential

Clear selection

Does your paper address subitem 6a-iii?

Copy and paste relevant sections from manuscript text

Qualitative feedback was collected post-study to inform broader evaluation of the intervention but is outside the scope of the current analysis.

6b) Any changes to trial outcomes after the trial commenced, with reasons

Does your paper address CONSORT subitem 6b? *

Copy and paste relevant sections from the manuscript (include quotes in quotation marks "like this" to indicate direct quotes from your manuscript), or elaborate on this item by providing additional information not in the ms, or briefly explain why the item is not applicable/relevant for your study

Results: Goal Attainment: The Kitchen Helper Program goal had to be withdrawn after 2 weeks due to technical issues with device response timing and voice command difficulties navigating recipes.

7a) How sample size was determined

NPT: When applicable, details of whether and how the clustering by care provides or centers was addressed

7a-i) Describe whether and how expected attrition was taken into account when calculating the sample size

Describe whether and how expected attrition was taken into account when calculating the sample size.

	1	2	3	4	5	
subitem not at all important	○	○	○	●	○	essential

Clear selection

Does your paper address subitem 7a-i?

Copy and paste relevant sections from manuscript title (include quotes in quotation marks "like this" to indicate direct quotes from your manuscript), or elaborate on this item by providing additional information not in the ms, or briefly explain why the item is not applicable/relevant for your study

As a feasibility trial, attrition was not taken into consideration: Methods: Sample Size: "We recruited 30 participants (n=15 in each arm) consistent with sample size guidelines for pilot feasibility trials [47], and sufficient to provide preliminary estimates of medium effect sizes (d=0.5) to inform the design of a fully powered RCT"

7b) When applicable, explanation of any interim analyses and stopping guidelines

Does your paper address CONSORT subitem 7b? *

Copy and paste relevant sections from the manuscript (include quotes in quotation marks "like this" to indicate direct quotes from your manuscript), or elaborate on this item by providing additional information not in the ms, or briefly explain why the item is not applicable/relevant for your study

No interim analyses or stopping guidelines were planned or conducted.

8a) Method used to generate the random allocation sequence

NPT: When applicable, how care providers were allocated to each trial group

Does your paper address CONSORT subitem 8a? *

Copy and paste relevant sections from the manuscript (include quotes in quotation marks "like this" to indicate direct quotes from your manuscript), or elaborate on this item by providing additional information not in the ms, or briefly explain why the item is not applicable/relevant for your study

Method: Study Design and Randomization: "The random allocation sequence was generated in advance using a computer-based randomization method in Microsoft Excel"

8b) Type of randomisation; details of any restriction (such as blocking and block size)

Does your paper address CONSORT subitem 8b? *

Copy and paste relevant sections from the manuscript (include quotes in quotation marks "like this" to indicate direct quotes from your manuscript), or elaborate on this item by providing additional information not in the ms, or briefly explain why the item is not applicable/relevant for your study

Method: Study Design and Randomization: Participants were enrolled by the primary project lead. Following completion of baseline assessments, "participants were block randomized (1:1), stratified by gender, to either the intervention (VOICE-COG program) or control group."

9) Mechanism used to implement the random allocation sequence (such as sequentially numbered containers), describing any steps taken to conceal the sequence until interventions were assigned

Does your paper address CONSORT subitem 9? *

Copy and paste relevant sections from the manuscript (include quotes in quotation marks "like this" to indicate direct quotes from your manuscript), or elaborate on this item by providing additional information not in the ms, or briefly explain why the item is not applicable/relevant for your study

Method: Study Design and Randomization: The random allocation sequence was generated in advance using a computer-based randomization method in Microsoft Excel "by a research assistant who was independent of intervention delivery. The research assistant assigned participants according to the pre-generated sequence and notified the project lead of group allocation"

10) Who generated the random allocation sequence, who enrolled participants, and who assigned participants to interventions

Does your paper address CONSORT subitem 10? *

Copy and paste relevant sections from the manuscript (include quotes in quotation marks "like this" to indicate direct quotes from your manuscript), or elaborate on this item by providing additional information not in the ms, or briefly explain why the item is not applicable/relevant for your study

Method: Study Design and Randomization: Participants were enrolled by the primary project lead. Following completion of baseline assessments, participants were block randomized (1:1), stratified by gender, to either the intervention (VOICE-COG program) or control group. The random allocation sequence was generated in advance using a computer-based randomization method in Microsoft Excel by a research assistant who was independent of intervention delivery. The research assistant assigned participants according to the pre-generated sequence and notified the project lead of group allocation.

11a) If done, who was blinded after assignment to interventions (for example, participants, care providers, those assessing outcomes) and how

NPT: Whether or not administering co-interventions were blinded to group assignment

11a-i) Specify who was blinded, and who wasn't

Specify who was blinded, and who wasn't. Usually, in web-based trials it is not possible to blind the participants [1, 3] (this should be clearly acknowledged), but it may be possible to blind outcome assessors, those doing data analysis or those administering co-interventions (if any).

	1	2	3	4	5	
subitem not at all important	○	○	○	○	◉	essential

Clear selection

Does your paper address subitem 11a-i? *

Copy and paste relevant sections from the manuscript (include quotes in quotation marks "like this" to indicate direct quotes from your manuscript), or elaborate on this item by providing additional information not in the ms, or briefly explain why the item is not applicable/relevant for your study

This 12-week "non-blinded" pilot RCT

11a-ii) Discuss e.g., whether participants knew which intervention was the "intervention of interest" and which one was the "comparator"

Informed consent procedures (4a-ii) can create biases and certain expectations - discuss e.g., whether participants knew which intervention was the "intervention of interest" and which one was the "comparator".

	1	2	3	4	5	
subitem not at all important	○	○	○	○	◉	essential

Clear selection

Does your paper address subitem 11a-ii?

Copy and paste relevant sections from the manuscript (include quotes in quotation marks "like this" to indicate direct quotes from your manuscript), or elaborate on this item by providing additional information not in the ms, or briefly explain why the item is not applicable/relevant for your study

Participants were not blinded to group allocation due to the nature of the intervention.

11b) If relevant, description of the similarity of interventions

(this item is usually not relevant for ehealth trials as it refers to similarity of a placebo or sham intervention to a active medication/intervention)

Does your paper address CONSORT subitem 11b? *

Copy and paste relevant sections from the manuscript (include quotes in quotation marks "like this" to indicate direct quotes from your manuscript), or elaborate on this item by providing additional information not in the ms, or briefly explain why the item is not applicable/relevant for your study

Interventions were not similar in this study.

12a) Statistical methods used to compare groups for primary and secondary outcomes

NPT: When applicable, details of whether and how the clustering by care providers or centers was addressed

Does your paper address CONSORT subitem 12a? *

Copy and paste relevant sections from the manuscript (include quotes in quotation marks "like this" to indicate direct quotes from your manuscript), or elaborate on this item by providing additional information not in the ms, or briefly explain why the item is not applicable/relevant for your study

Methods: Data Analysis: Descriptive data for feasibility and secondary outcomes are presented as either mean and standard deviation (SD) or frequency (%). Differences in GAS-Light t-scores for the intervention group were compared using a paired sample t-test. SUS scores were entered into the web-based analysis toolkit [49] to provide a box plot for visual comparison with the adjective quartile SUS score [50]. Differences in participant and care partner questionnaires were compared between groups using ANCOVA adjusted for baseline values and standardized effect sizes calculated using Cohen d.

12a-i) Imputation techniques to deal with attrition / missing values

Imputation techniques to deal with attrition / missing values: Not all participants will use the intervention/comparator as intended and attrition is typically high in ehealth trials. Specify how participants who did not use the application or dropped out from the trial were treated in the statistical analysis (a complete case analysis is strongly discouraged, and simple imputation techniques such as LOCF may also be problematic [4]).

	1	2	3	4	5	
subitem not at all important	○	○	○	○	◉	essential

Clear selection

Does your paper address subitem 12a-i? *

Copy and paste relevant sections from the manuscript (include quotes in quotation marks "like this" to indicate direct quotes from your manuscript), or elaborate on this item by providing additional information not in the ms, or briefly explain why the item is not applicable/relevant for your study

Methods: Participant Questionnaires: Multifactorial Memory Questionnaire (MMQ) is memory evaluation tool with 57-items covering three sub-scales – memory satisfaction, ability, and strategy use [42]. Participants' subjective memory experiences from the previous 2 weeks are rated using a 5-point Likert scale from 0 to 4. "If there were <4 missing responses for any scale, scores were calculated using the pro-rated formula; for ≥ 4 missing items, participant scores were excluded from analysis [42]." The Illness Cognition Questionnaire (ICQ) is an 18-item questionnaire using a 4-point Likert scale from 1 (disagree) to 4 (strongly agree) to measure the respondents' perception of their own illness with three subscales of acceptance, disease benefit and helplessness [43]. "For participants with one missing item in a subscale, the missing item was imputed using the mean of the other completed items within that subscale (provided at least 4 of 6 items were present)." "Missing data within scales was replaced by the mean value of item responses for that sub-scale unless otherwise specified".

12b) Methods for additional analyses, such as subgroup analyses and adjusted analyses

Does your paper address CONSORT subitem 12b? *

Copy and paste relevant sections from the manuscript (include quotes in quotation marks "like this" to indicate direct quotes from your manuscript), or elaborate on this item by providing additional information not in the ms, or briefly explain why the item is not applicable/relevant for your study

Methods: Data Analysis: Shapiro-Wilk tests assessed assumptions of normality and homogeneity of variance, and non-normal data were transformed to meet normality assumptions of ANCOVA, where required

X26) REB/IRB Approval and Ethical Considerations [recommended as subheading under "Methods"] (not a CONSORT item)

X26-i) Comment on ethics committee approval

	1	2	3	4	5	
subitem not at all important	○	○	○	○	◉	essential

Clear selection

Does your paper address subitem X26-i?

Copy and paste relevant sections from the manuscript (include quotes in quotation marks "like this" to indicate direct quotes from your manuscript), or elaborate on this item by providing additional information not in the ms, or briefly explain why the item is not applicable/relevant for your study

Methods: Ethical Considerations: This study was approved by the XX Human Research Ethics Committee (HREC 2022-xxx) and registered with the ANZCTR (Australian New Zealand Clinical Trials Registry; xx).

x26-ii) Outline informed consent procedures

Outline informed consent procedures e.g., if consent was obtained offline or online (how? Checkbox, etc.?), and what information was provided (see 4a-ii). See [6] for some items to be included in informed consent documents.

	1	2	3	4	5	
subitem not at all important	○	○	○	◉	○	essential

Clear selection

Does your paper address subitem X26-ii?

Copy and paste relevant sections from the manuscript (include quotes in quotation marks "like this" to indicate direct quotes from your manuscript), or elaborate on this item by providing additional information not in the ms, or briefly explain why the item is not applicable/relevant for your study

Methods: Participants: Interested participants were screened for eligibility via telephone and then emailed a link to the Plain Language and Consent Form on Qualtrics "to provide written or electronic informed consent."

X26-iii) Safety and security procedures

Safety and security procedures, incl. privacy considerations, and any steps taken to reduce the likelihood or detection of harm (e.g., education and training, availability of a hotline)

	1	2	3	4	5	
subitem not at all important	○	○	○	◉	○	essential

Clear selection

Does your paper address subitem X26-iii?

Copy and paste relevant sections from the manuscript (include quotes in quotation marks "like this" to indicate direct quotes from your manuscript), or elaborate on this item by providing additional information not in the ms, or briefly explain why the item is not applicable/relevant for your study

Methods: Intervention: i) Each participant's goal-related content was uploaded remotely to the "Buddy Link" software (Great Australian Pty Ltd) [23] by a research team member, who also configured program broadcast routines using "deidentified individual Alexa accounts".
ii) Following the meeting, the intervention group were "emailed a personalized two-page instruction summary" for the selected VOICE-COG goals.
iii) Throughout the trial, all intervention participants received fortnightly emails "requesting notification of technical errors via completion of an online Qualtrics form".
iv) An "optional mid-trial check-in by a research team member was offered to participants to address any concerns" regarding their program.

RESULTS

13a) For each group, the numbers of participants who were randomly assigned, received intended treatment, and were analysed for the primary outcome
NPT: The number of care providers or centers performing the intervention in each group and the number of patients treated by each care provider in each center

Does your paper address CONSORT subitem 13a? *

Copy and paste relevant sections from the manuscript (include quotes in quotation marks "like this" to indicate direct quotes from your manuscript), or elaborate on this item by providing additional information not in the ms, or briefly explain why the item is not applicable/relevant for your study

Results: Participant Recruitment: "In total, n=145 expressions of interest were received with n=99 potential participants successfully contacted and screened between September 2023 and June 2024. The recruitment rate was 86%, i.e., of the 35 eligible participants, 30 people living with dementia/MCI and their care partners provided consent to participate. A total of N=30 participants completed baseline assessments and were randomly allocated to DVA group (n=15) or control (n=15)."

13b) For each group, losses and exclusions after randomisation, together with reasons

Does your paper address CONSORT subitem 13b? (NOTE: Preferably, this is shown in a CONSORT flow diagram) *

Copy and paste relevant sections from the manuscript (include quotes in quotation marks "like this" to indicate direct quotes from your manuscript), or elaborate on this item by providing additional information not in the ms, or briefly explain why the item is not applicable/relevant for your study

Results: Primary Feasibility Outcomes: "The overall retention rate was 97% (29/30). Retention was 100% for the control group and 93% for the intervention group with one participant lost to follow-up (reason unknown)."

13b-i) Attrition diagram

Strongly recommended: An attrition diagram (e.g., proportion of participants still logging in or using the intervention/comparator in each group plotted over time, similar to a survival curve) or other figures or tables demonstrating usage/dose/engagement.

	1	2	3	4	5	
subitem not at all important	○	○	○	○	◉	essential

Clear selection

Does your paper address subitem 13b-i?

Copy and paste relevant sections from the manuscript or cite the figure number if applicable (include quotes in quotation marks "like this" to indicate direct quotes from your manuscript), or elaborate on this item by providing additional information not in the ms, or briefly explain why the item is not applicable/relevant for your study

Figure 1: CONSORT Flow Diagram

14a) Dates defining the periods of recruitment and follow-up

Does your paper address CONSORT subitem 14a? *

Copy and paste relevant sections from the manuscript (include quotes in quotation marks "like this" to indicate direct quotes from your manuscript), or elaborate on this item by providing additional information not in the ms, or briefly explain why the item is not applicable/relevant for your study

Results: Participant Recruitment: In total, n=145 expressions of interest were received with n=99 potential participants successfully contacted and screened between "September 2023 and June 2024"

14a-i) Indicate if critical “secular events” fell into the study period

Indicate if critical “secular events” fell into the study period, e.g., significant changes in Internet resources available or “changes in computer hardware or Internet delivery resources”

	1	2	3	4	5	
subitem not at all important	○	○	○	◉	○	essential

Clear selection

Does your paper address subitem 14a-i?

Copy and paste relevant sections from the manuscript (include quotes in quotation marks "like this" to indicate direct quotes from your manuscript), or elaborate on this item by providing additional information not in the ms, or briefly explain why the item is not applicable/relevant for your study

No critical secular events occurred during this period.

14b) Why the trial ended or was stopped (early)

Does your paper address CONSORT subitem 14b? *

Copy and paste relevant sections from the manuscript (include quotes in quotation marks "like this" to indicate direct quotes from your manuscript), or elaborate on this item by providing additional information not in the ms, or briefly explain why the item is not applicable/relevant for your study

This trial did not end or stop early.

15) A table showing baseline demographic and clinical characteristics for each group

NPT: When applicable, a description of care providers (case volume, qualification, expertise, etc.) and centers (volume) in each group

Does your paper address CONSORT subitem 15? *

Copy and paste relevant sections from the manuscript (include quotes in quotation marks "like this" to indicate direct quotes from your manuscript), or elaborate on this item by providing additional information not in the ms, or briefly explain why the item is not applicable/relevant for your study

Table 1 details baseline demographic characteristics for each group

15-i) Report demographics associated with digital divide issues

In ehealth trials it is particularly important to report demographics associated with digital divide issues, such as age, education, gender, social-economic status, computer/Internet/ehealth literacy of the participants, if known.

	1	2	3	4	5	
subitem not at all important	○	○	○	○	◉	essential

Clear selection

Does your paper address subitem 15-i? *

Copy and paste relevant sections from the manuscript (include quotes in quotation marks "like this" to indicate direct quotes from your manuscript), or elaborate on this item by providing additional information not in the ms, or briefly explain why the item is not applicable/relevant for your study

Results: Participant demographics: "Participants' ages ranged from 60 to 85 years (mean 74.7, SD 5.2 years) and the majority (63%, 19/30) were women. Over half (60%, 18/30) were educated to university level and whilst most (80%, 24/30) participants were retired, 20% (2/30) were still employed."

16) For each group, number of participants (denominator) included in each analysis and whether the analysis was by original assigned groups

16-i) Report multiple “denominators” and provide definitions

Report multiple “denominators” and provide definitions: Report N’s (and effect sizes) “across a range of study participation [and use] thresholds” [1], e.g., N exposed, N consented, N used more than x times, N used more than y weeks, N participants “used” the intervention/comparator at specific pre-defined time points of interest (in absolute and relative numbers per group). Always clearly define “use” of the intervention.

	1	2	3	4	5	
subitem not at all important	○	○	○	○	◉	essential

Clear selection

Does your paper address subitem 16-i? *

Copy and paste relevant sections from the manuscript (include quotes in quotation marks "like this" to indicate direct quotes from your manuscript), or elaborate on this item by providing additional information not in the ms, or briefly explain why the item is not applicable/relevant for your study

Results: Primary Feasibility Outcomes: "Adherence to the VOICE-COG program ranged from 29 to 100%, with a mean of 70% (SD 17%)

16-ii) Primary analysis should be intent-to-treat

Primary analysis should be intent-to-treat, secondary analyses could include comparing only “users”, with the appropriate caveats that this is no longer a randomized sample (see 18-i).

	1	2	3	4	5	
subitem not at all important	○	○	○	○	◉	essential

Clear selection

Does your paper address subitem 16-ii?

Copy and paste relevant sections from the manuscript (include quotes in quotation marks "like this" to indicate direct quotes from your manuscript), or elaborate on this item by providing additional information not in the ms, or briefly explain why the item is not applicable/relevant for your study

Results: Primary Feasibility Outcomes: The overall retention rate was 97% (29/30). Retention was 100% for the control group and 93% for the intervention group with one participant lost to follow-up (reason unknown).

17a) For each primary and secondary outcome, results for each group, and the estimated effect size and its precision (such as 95% confidence interval)

Does your paper address CONSORT subitem 17a? *

Copy and paste relevant sections from the manuscript (include quotes in quotation marks "like this" to indicate direct quotes from your manuscript), or elaborate on this item by providing additional information not in the ms, or briefly explain why the item is not applicable/relevant for your study

Table 2 details secondary outcome results for each group with estimated effect sizes and 95% Confidence Intervals.

17a-i) Presentation of process outcomes such as metrics of use and intensity of use

In addition to primary/secondary (clinical) outcomes, the presentation of process outcomes such as metrics of use and intensity of use (dose, exposure) and their operational definitions is critical. This does not only refer to metrics of attrition (13-b) (often a binary variable), but also to more continuous exposure metrics such as “average session length”. These must be accompanied by a technical description how a metric like a “session” is defined (e.g., timeout after idle time) [1] (report under item 6a).

	1	2	3	4	5	
subitem not at all important	○	○	○	◉	○	essential

Clear selection

Does your paper address subitem 17a-i?

Copy and paste relevant sections from the manuscript (include quotes in quotation marks "like this" to indicate direct quotes from your manuscript), or elaborate on this item by providing additional information not in the ms, or briefly explain why the item is not applicable/relevant for your study

Figure 2 provides participant adherence and adherence to each VOICE-COG program goal.

17b) For binary outcomes, presentation of both absolute and relative effect sizes is recommended

Does your paper address CONSORT subitem 17b? *

Copy and paste relevant sections from the manuscript (include quotes in quotation marks "like this" to indicate direct quotes from your manuscript), or elaborate on this item by providing additional information not in the ms, or briefly explain why the item is not applicable/relevant for your study

Binary outcomes were not reported

18) Results of any other analyses performed, including subgroup analyses and adjusted analyses, distinguishing pre-specified from exploratory

Does your paper address CONSORT subitem 18? *

Copy and paste relevant sections from the manuscript (include quotes in quotation marks "like this" to indicate direct quotes from your manuscript), or elaborate on this item by providing additional information not in the ms, or briefly explain why the item is not applicable/relevant for your study

Your answer

18-i) Subgroup analysis of comparing only users

A subgroup analysis of comparing only users is not uncommon in ehealth trials, but if done, it must be stressed that this is a self-selected sample and no longer an unbiased sample from a randomized trial (see 16-iii).

	1	2	3	4	5	
subitem not at all important	○	○	○	◉	○	essential

Clear selection

Does your paper address subitem 18-i?

Copy and paste relevant sections from the manuscript (include quotes in quotation marks "like this" to indicate direct quotes from your manuscript), or elaborate on this item by providing additional information not in the ms, or briefly explain why the item is not applicable/relevant for your study

No subgroup analyses were performed.

19) All important harms or unintended effects in each group

(for specific guidance see CONSORT for harms)

Does your paper address CONSORT subitem 19? *

Copy and paste relevant sections from the manuscript (include quotes in quotation marks "like this" to indicate direct quotes from your manuscript), or elaborate on this item by providing additional information not in the ms, or briefly explain why the item is not applicable/relevant for your study

Results: Primary Feasibility Outcomes: Adherence to the VOICE-COG program ranged from 29 to 100%, with a mean of 70% (SD 17%), exceeding the target adherence of 66%, "with no adverse events."

19-i) Include privacy breaches, technical problems

Include privacy breaches, technical problems. This does not only include physical "harm" to participants, but also incidents such as perceived or real privacy breaches [1], technical problems, and other unexpected/unintended incidents. "Unintended effects" also includes unintended positive effects [2].

	1	2	3	4	5	
subitem not at all important	○	○	○	◉	○	essential

Clear selection

Does your paper address subitem 19-i?

Copy and paste relevant sections from the manuscript (include quotes in quotation marks "like this" to indicate direct quotes from your manuscript), or elaborate on this item by providing additional information not in the ms, or briefly explain why the item is not applicable/relevant for your study

Results: Goal Attainment: The Kitchen Helper Program goal had to be withdrawn after 2 weeks due to technical issues with device response timing and voice command difficulties navigating recipes

19-ii) Include qualitative feedback from participants or observations from staff/researchers

Include qualitative feedback from participants or observations from staff/researchers, if available, on strengths and shortcomings of the application, especially if they point to unintended/unexpected effects or uses. This includes (if available) reasons for why people did or did not use the application as intended by the developers.

	1	2	3	4	5	
subitem not at all important	○	○	◉	○	○	essential

Clear selection

Does your paper address subitem 19-ii?

Copy and paste relevant sections from the manuscript (include quotes in quotation marks "like this" to indicate direct quotes from your manuscript), or elaborate on this item by providing additional information not in the ms, or briefly explain why the item is not applicable/relevant for your study

Discussion (Paragraph 4): Finally, in alignment with the IDEAS framework, a subsequent qualitative analysis of user experiences will be conducted to inform further development of VOICE-COG for evaluation in large-scale RCT.

DISCUSSION

22) Interpretation consistent with results, balancing benefits and harms, and considering other relevant evidence

NPT: In addition, take into account the choice of the comparator, lack of or partial blinding, and unequal expertise of care providers or centers in each group

22-i) Restate study questions and summarize the answers suggested by the data, starting with primary outcomes and process outcomes (use)

Restate study questions and summarize the answers suggested by the data, starting with primary outcomes and process outcomes (use).

	1	2	3	4	5	
subitem not at all important	○	○	○	○	◉	essential

Clear selection

Does your paper address subitem 22-i? *

Copy and paste relevant sections from the manuscript (include quotes in quotation marks "like this" to indicate direct quotes from your manuscript), or elaborate on this item by providing additional information not in the ms, or briefly explain why the item is not applicable/relevant for your study

Discussion (paragraph 1) : "Findings from this pilot RCT demonstrate the feasibility of a co-designed cognitive reablement program broadcast via DVAs to support older adults living with dementia/MCI. The target sample was successfully recruited and a high retention rate achieved. Adherence, assessed as weekly interaction with program goals exceeded target levels (66%), and the mean SUS score indicated marginal acceptability. "

22-ii) Highlight unanswered new questions, suggest future research

Highlight unanswered new questions, suggest future research.

	1	2	3	4	5	
subitem not at all important	○	○	○	○	◉	essential

Clear selection

Does your paper address subitem 22-ii?

Copy and paste relevant sections from the manuscript (include quotes in quotation marks "like this" to indicate direct quotes from your manuscript), or elaborate on this item by providing additional information not in the ms, or briefly explain why the item is not applicable/relevant for your study

Discussion: Implications for Further Research: "Feasibility results from this study warrant a full-scale trial, to evaluate whether VOICE-COG, relative to usual care, can support enhanced cognition and functioning in people living with dementia/MCI living independently in the community over longer time frames. Effect sizes found in this study may be used to inform the sample size for a future trial. Recruitment via clinically facilitated partnerships, such as memory clinics, should be considered to reduce selection bias from web-based advertisements. This should include increasing accessibility of VOICE-COG to a more diverse population including minority ethnic groups or lower socioeconomic status, and those in rural areas. Digital literacy measures should be considered in conjunction with qualitative feedback to determine factors influencing adoption. Finally, the cost-effectiveness of this personalized goal-based cognitive reablement program compared to stand-alone DVA implementation should be evaluated to determine if they should be implemented at scale."

20) Trial limitations, addressing sources of potential bias, imprecision, and, if relevant, multiplicity of analyses

20-i) Typical limitations in ehealth trials

Typical limitations in ehealth trials: Participants in ehealth trials are rarely blinded. Ehealth trials often look at a multiplicity of outcomes, increasing risk for a Type I error. Discuss biases due to non-use of the intervention/usability issues, biases through informed consent procedures, unexpected events.

	1	2	3	4	5	
subitem not at all important	○	○	○	○	●	essential

Clear selection

Does your paper address subitem 20-i? *

Copy and paste relevant sections from the manuscript (include quotes in quotation marks "like this" to indicate direct quotes from your manuscript), or elaborate on this item by providing additional information not in the ms, or briefly explain why the item is not applicable/relevant for your study

Discussion: Limitations: "Goal attainment measures were self-reported and could have been positively influenced by a social desirability bias. Furthermore, control group participants did not set reablement goals, so we could not ascertain if goal attainment differences were associated with the intervention or the goal-setting process. Participants had not previously owned an Amazon Alexa Show 8 DVA, so SUS responses may have been influenced by the DVA's integrated features and functionality."

21) Generalisability (external validity, applicability) of the trial findings

NPT: External validity of the trial findings according to the intervention, comparators, patients, and care providers or centers involved in the trial

21-i) Generalizability to other populations

Generalizability to other populations: In particular, discuss generalizability to a general Internet population, outside of a RCT setting, and general patient population, including applicability of the study results for other organizations

	1	2	3	4	5	
subitem not at all important	○	○	○	○	◉	essential

Clear selection

Does your paper address subitem 21-i?

Copy and paste relevant sections from the manuscript (include quotes in quotation marks "like this" to indicate direct quotes from your manuscript), or elaborate on this item by providing additional information not in the ms, or briefly explain why the item is not applicable/relevant for your study

Discussion: Limitations: Recruitment methods in this study used web-based applications that required a level of digital literacy, which could limit generalization of these findings to all people living with dementia/MCI. This may have led to an unintended bias of a highly educated sample potentially limiting generalization to all people living with dementia/MCI.

21-ii) Discuss if there were elements in the RCT that would be different in a routine application setting

Discuss if there were elements in the RCT that would be different in a routine application setting (e.g., prompts/reminders, more human involvement, training sessions or other co-interventions) and what impact the omission of these elements could have on use, adoption, or outcomes if the intervention is applied outside of a RCT setting.

	1	2	3	4	5	
subitem not at all important	○	○	○	◉	○	essential

Clear selection

Does your paper address subitem 21-ii?

Copy and paste relevant sections from the manuscript (include quotes in quotation marks "like this" to indicate direct quotes from your manuscript), or elaborate on this item by providing additional information not in the ms, or briefly explain why the item is not applicable/relevant for your study

Discussion: Limitations: Finally, as a pilot feasibility trial of a minimum viable product, additional human support elements included researcher input during program configuration. Future trials should consider an integrated configuration interface, enabling direct transfer of data from web-based forms into the Buddylink software for real-world implementation.

OTHER INFORMATION

23) Registration number and name of trial registry

Does your paper address CONSORT subitem 23? *

Copy and paste relevant sections from the manuscript (include quotes in quotation marks "like this" to indicate direct quotes from your manuscript), or elaborate on this item by providing additional information not in the ms, or briefly explain why the item is not applicable/relevant for your study

Trial Registration: ACTRN12623000898651

24) Where the full trial protocol can be accessed, if available

Does your paper address CONSORT subitem 24? *

Cite a Multimedia Appendix, other reference, or copy and paste relevant sections from the manuscript (include quotes in quotation marks "like this" to indicate direct quotes from your manuscript), or elaborate on this item by providing additional information not in the ms, or briefly explain why the item is not applicable/relevant for your study

The full trial protocol can be accessed from Deakin University Human Research Ethics Committee (DUHREC 2022-043)

25) Sources of funding and other support (such as supply of drugs), role of funders

Does your paper address CONSORT subitem 25? *

Copy and paste relevant sections from the manuscript (include quotes in quotation marks "like this" to indicate direct quotes from your manuscript), or elaborate on this item by providing additional information not in the ms, or briefly explain why the item is not applicable/relevant for your study

Dementia Australia Research Foundation Project Grant

X27) Conflicts of Interest (not a CONSORT item)

X27-i) State the relation of the study team towards the system being evaluated

In addition to the usual declaration of interests (financial or otherwise), also state the relation of the study team towards the system being evaluated, i.e., state if the authors/evaluators are distinct from or identical with the developers/sponsors of the intervention.

	1	2	3	4	5	
subitem not at all important	○	○	○	◉	○	essential

Clear selection

Does your paper address subitem X27-i?

Copy and paste relevant sections from the manuscript (include quotes in quotation marks "like this" to indicate direct quotes from your manuscript), or elaborate on this item by providing additional information not in the ms, or briefly explain why the item is not applicable/relevant for your study

The study team were independent from the software developer/sponsor of the intervention. Conflicts of Interest: EG is a director (unpaid position) of Great Australian Pty Ltd. Great Australian Pty Ltd provided in-kind technical support for software and had a nonfinancial interest in the development of a voice-based digital assistant software for telehealth. Great Australian had no input into this study's design, analysis, or interpretation of the results, or the decision to publish.

About the CONSORT EHEALTH checklist

As a result of using this checklist, did you make changes in your manuscript? *

- [ ] yes, major changes
- [x] yes, minor changes
- [ ] no

What were the most important changes you made as a result of using this checklist?

Clarifying human involvement for the personalised intervention content and implications for a future trial.

How much time did you spend on going through the checklist INCLUDING making changes in your manuscript *

I would estimate four hours

As a result of using this checklist, do you think your manuscript has improved? *

- [x] yes
- [ ] no
- [ ] Other:

Would you like to become involved in the CONSORT EHEALTH group?

This would involve for example becoming involved in participating in a workshop and writing an "Explanation and Elaboration" document

- ○ yes
- ○ no
- ◉ Other: Not currently but potentially in future

Clear selection

Any other comments or questions on CONSORT EHEALTH

Could E-health consort training be available as a pre-requisite for anyone undertaking digital health interventions.

STOP - Save this form as PDF before you click submit

To generate a record that you filled in this form, we recommend to generate a PDF of this page (on a Mac, simply select "print" and then select "print as PDF") before you submit it.

When you submit your (revised) paper to JMIR, please upload the PDF as supplementary file.

Don't worry if some text in the textboxes is cut off, as we still have the complete information in our database. Thank you!

Final step: Click submit !

Click submit so we have your answers in our database!

Submit

Clear form

Never submit passwords through Google Forms.

This content is neither created nor endorsed by Google. - Terms of Service - Privacy Policy

Does this form look suspicious? Report

Google Forms
